# Time-Restricted Eating and Sleep, Mood, and Quality of Life in Adults With Overweight or Obesity: A Secondary Analysis of a Randomized Clinical Trial

**DOI:** 10.1001/jamanetworkopen.2025.17268

**Published:** 2025-06-25

**Authors:** Antonio Clavero-Jimeno, Manuel Dote-Montero, Jairo H. Migueles, Alba Camacho-Cardenosa, María Medrano, Víctor Manuel Alfaro-Magallanes, Maddi Osés, Almudena Carneiro-Barrera, Rafael de Cabo, Manuel Muñoz-Torres, Idoia Labayen, Jonatan R. Ruiz

**Affiliations:** 1 Department of Physical Education and Sports, Faculty of Sport Sciences, Sport and Health University Research Institute (iMUDS), University of Granada, Granada, Spain; 2 Obesity and Diabetes Clinical Research Section, Phoenix Epidemiology and Clinical Research Branch, National Institute of Diabetes and Digestive and Kidney Diseases, National Institutes of Health, Phoenix, Arizona; 3 Instituto de Investigación Biosanitaria, Ibs.Granada, Granada, Spain; 4 Institute for Sustainability & Food Chain Innovation, Department of Health Sciences, Public University of Navarre, Pamplona, Spain; 5 Navarra Institute for Health Research (IdiSNA), Pamplona, Spain; 6 Centro de Investigación Biomédica en Red Fisiopatología de la Obesidad y Nutrición (CIBERobn), Instituto de Salud Carlos III, Madrid, Spain; 7 LFE Research Group, Department of Health and Human Performance, Faculty of Physical Activity and Sport Sciences, Universidad Politécnica de Madrid, Madrid, Spain; 8 Department of Psychology, Universidad Loyola Andalucía, Seville, Spain; 9 Translational Gerontology Branch, National Institute on Aging, National Institutes of Health, Baltimore, Maryland; 10 Endocrinology and Nutrition Unit, University Hospital San Cecilio Clinic, Granada, Spain; 11 Department of Medicine, University of Granada, Granada, Spain; 12 CIBER on Frailty and Healthy Aging (CIBERFES), Carlos III Health Institute, Madrid, Spain

## Abstract

**Importance:**

It remains largely unknown whether the timing of the eating window during time-restricted eating (TRE) affects sleep, mood, and quality of life differently.

**Objective:**

To investigate whether 3 TRE schedules—an 8-hour eating window in the early part of the day, the late part of the day, or selected by the participant—combined with usual care (UC; a Mediterranean diet education program) were associated with changes in sleep, mood, and quality of life compared with UC alone in adults with overweight or obesity over a 12-week intervention.

**Design, Setting, and Participants:**

This was a prespecified secondary analysis of a parallel-group randomized clinical trial conducted from April 11, 2022, to March 6, 2023, in Granada (southern Spain) and Pamplona (northern Spain). Eligible participants were men and women aged 30 to 60 years with overweight or obesity. Data analyses for the current study were performed between March 14, 2024, and December 5, 2024.

**Intervention:**

Participants in the UC group maintained their usual eating window (≥12 hours daily) and received an educational program based on the Mediterranean diet. Participants in the early TRE group had an 8-hour eating window starting before 10 am and the late TRE group, after 1 pm; self-selected TRE participants chose their own 8-hour window. All TRE participants also received the UC educational program. Group allocation was 1:1:1:1.

**Main Outcomes and Measures:**

Changes in sleep were objectively assessed using accelerometry for 2 weeks before the intervention and during the final 2 weeks of the intervention. Changes in mood dimensions—depression, anxiety, and stress—and quality of life were assessed before and after the 12-week intervention using self-administered questionnaires.

**Results:**

A total of 197 participants (98 women [49.7%]; mean [SD] age, 46.1 [8.4] years; mean [SD] body mass index, 32.8 [3.2], calculated as weight in kilograms divided by height in meters squared) were randomized to UC (n = 49), early TRE (n = 49), late TRE (n = 52), and self-selected TRE (n = 47). No significant differences were observed between the early TRE and UC groups for sleep (eg, mean difference in total sleep time, 0.2 [95% CI, –0.2 to 0.6] hours), mood (eg, mean difference in Beck Depression Inventory Fast Screen score, 0.2 [95% CI, –1.0 to 1.3] points; mean difference in state anxiety score on the State-Trait Anxiety Inventory, –1.2 [95% CI, –6.4 to 4.1] points; mean difference in Perceived Stress Scale score, 2.1 [95% CI, –1.8 to 5.9] points), and quality of life (eg, mean difference in general health score on the Rand 36-Item Short Form Health Survey, 3.3 [95% CI, –4.4 to 10.9] points). Results were also nonsignificant for the late TRE and self-selected TRE groups compared with the UC group as well as between the TRE groups.

**Conclusions and Relevance:**

In this secondary analysis of a randomized clinical trial of 3 different TRE schedules, incorporating TRE into a UC intervention, regardless of the eating window timing, was not associated with significant changes in sleep, mood, or quality of life compared with UC alone in adults with overweight or obesity. The findings suggest TRE may be a viable nutritional weight management strategy without adverse effects on sleep, mood, or quality of life.

**Trial Registration:**

ClinicalTrials.gov Identifier: NCT05310721

## Key Points

**Question:**

Are early, late, or self-selected time-restricted eating (TRE) schedules combined with usual care (UC) associated with differences in sleep, mood, and quality of life compared with UC alone?

**Findings:**

In this secondary analysis of a randomized clinical trial of 197 adults with overweight or obesity randomized to UC alone or combined with early, late, or self-selected TRE, no significant differences were observed in sleep, mood, and quality of life between groups.

**Meaning:**

These findings suggest incorporating TRE into a UC intervention, regardless of eating window timing, may be a viable weight management strategy without adverse effects on sleep, mood, or quality of life.

## Teaser

This secondary analysis of a randomized clinical trial assesses whether time-restricted eating windows early or late in the day or at a self-selected time are associated with differences in sleep, mood, and quality-of-life measures compared with usual care among adults with overweight or obesity.

## Introduction

Obesity is often associated with insufficient sleep, increased mood disturbances, and a lower quality of life.^1,2,3^ Caloric restriction programs leading to body weight loss appear to improve sleep, mood, and quality of life in adults with excessive adiposity.^4,5,6^ However, long-term adherence to caloric restriction interventions remains limited,^7,8^ and weight regain after short-term loss is associated with poor sleep, mood disorder, and lower quality of life.^9^

Time-restricted eating (TRE) has emerged as a promising nutritional strategy for weight loss, allowing ad libitum food intake within a limited eating window of 4 to 10 hours per day, followed by fasting for the remaining 14 to 20 hours.^10^ TRE has been associated with unintentional reduction of energy intake,^11^ inducing subsequent weight loss and modest improvements in cardiometabolic health, which could lead to improvements in sleep, mood, and quality of life.^12^ Concerns remain that limiting eating to 4 to 10 hours per day may increase intake of energy-dense foods and caffeine to boost energy, potentially disrupting sleep, mood, and quality of life.^11,13^ However, it remains uncertain whether the timing of the eating window could differently impact sleep, mood, and quality of life.

Earlier eating windows may improve sleep by aligning meal timing with the body’s natural circadian rhythms.^14^ Eating earlier in the day reduces the overlap between meal times and the body’s melatonin production, which typically increases in the evening to signal the onset of sleep.^15^ This alignment may improve nighttime glycemic control by reducing melatonin-insulin interference.^16^ Early meals can also enhance satiety and reduce late-night eating, associated with disrupted sleep and postprandial hyperglycemia.^17,18^ These factors may contribute to better overall sleep efficiency.^16^ Preliminary evidence suggests that TRE does not adversely affect sleep quality in adults with overweight or obesity,^19,20,21,22,23^ yet the timing of the eating window may influence these findings, as studies have varied in their use of early TRE windows (eg, 7 am to 3 pm or 10 am to 6 pm),^19,21^ late TRE windows (eg, 12 pm to 8 pm),^24^ or self-selected TRE windows.^20,25,26^

The impact of the eating window timing on mood and quality of life remains unclear. Women are more susceptible to sleep disturbances, mood disorders, and reduced quality of life,^27,28,29,30^ underscoring the need to consider sex when assessing the impact of weight loss interventions on these outcomes.^31^ Previous trials had notable limitations, including small sample sizes, short durations (≤8 weeks), and/or underrepresentation of women.^20,24,32,33^ Therefore, our team conducted a 12-week randomized clinical trial (RCT)^34^ including both sexes and objectively assessed sleep using actigraphy, unlike other studies that relied on subjective measures.^12,23,33,35^

Our team’s trial showed that early, late, and self-selected TRE groups led to approximately 3 kg greater body weight loss than usual care (UC) over 12 weeks, with no significant differences among TRE groups and high adherence (85%-88%).^34^ In this study, we investigated whether the 3 different TRE schedules—early, late, and self-selected—combined with UC (a Mediterranean diet education program) compared with UC alone were associated with changes in sleep, mood, and quality of life among men and women with overweight or obesity. We hypothesized that early TRE could improve sleep by aligning meal timing with circadian rhythms,^16^ whereas late TRE may disrupt this alignment, limiting benefits to sleep, mood, and quality of life.

## Methods

### Study Design and Participants

This study was a prespecified secondary analysis of a parallel-group, multicenter RCT (NCT05310721) conducted in Granada (southern Spain) and Pamplona (northern Spain).^34^ Further details on the trial rationale, design, and methods can be found in Supplement 1. The trial and the current secondary analysis were registered and approved by the ethics committees of each center in Spain (Andalusian Health Service, Provincial Research Ethics Committee of Granada, and the Clinical Research Ethics Committee of Navarra) and followed the Consolidated Standards of Reporting Trials (CONSORT) reporting guideline for RCTs. Participants provided written informed consent.

Eligible participants were men and women aged 30 to 60 years with body mass index (calculated as weight in kilograms divided by height in meters squared) of 25.0 or greater and less than 40.0, stable weight (loss or gain within 3%) for at least 3 months, less than 150 minutes per week of moderate-to-vigorous physical activity, a habitual eating window of 12 or more hours per day, and at least 1 cardiometabolic risk factor, such as high-density lipoprotein cholesterol concentration less than 40 mg/dL for men or less than 50 mg/dL for women (to convert to mg/L, multiply by 0.1), low-density lipoprotein cholesterol concentration greater than 100 mg/dL (to convert to mg/L, multiply by 0.1), serum triglycerides concentration of 150 mg/dL or greater (to convert to mmol/L, multiply by 0.0113), blood pressure greater than 130/85 mm Hg, impaired glucose homeostasis, or current use of medication for any of these conditions. Exclusion criteria included cardiovascular events, diabetes, conditions contraindicating fasting, enrollment in weight-management programs, pregnancy or lactation, shift work, major sleep or eating disorders, or caregiving requiring frequent nighttime care. Full criteria are in the study protocol.^36^

### Study Recruitment, Enrollment, and Randomization

Potential participants were recruited, enrolled, and randomized at each center from April 11, 2022, to December 5, 2022, with the data collection concluding on March 6, 2023. Further details about participant recruitment and enrollment are available elsewhere.^36^ In brief, when participants met the inclusion criteria, baseline assessments were conducted. Then, participants were randomly assigned to a specific group: (1) UC, (2) early TRE, (3) late TRE, or (4) self-selected TRE. Randomization was conducted using both stratifications for each site and sex and permuted blocks with random block sizes of 4 and 8. Within each block, each quarter of randomizations was randomly selected to be to 1 of the 4 possible groups, using a parallel design with a 1:1:1:1 allocation ratio.

### Assessment of Sleep, Mood, and Quality of Life

Sleep habits were monitored using a triaxial accelerometer (ActiGraph GT3X+ [ActiGraph LLC]). Participants wore the device on their nondominant wrist during the 2 weeks prior to the beginning of the intervention (lead-in period) and during the last 2 weeks of the intervention. Participants recorded their bedtime and wake-up time daily using a custom mobile phone app (com.nnbi.app_extreme_granada [NNBi 2020 SL]). The GGIR package in R, version 3.1-6 (R Project for Statistical Computing)^37^ was used to process the raw accelerations (eTable 17 in Supplement 3 provides GGIR configuration details). We reported accelerometry-derived sleep outcomes such as sleep onset, sleep offset, sleep period (ie, time from sleep onset to sleep offset), total sleep time (ie, the amount of time classified as sleep within the sleep period), sleep efficiency after sleep onset (ie, [total sleep time / sleep period] × 100), number of awakenings, and time awake after sleep onset. We used sleep period instead of time in bed to calculate sleep efficiency, as time in bed was not directly available from accelerometry or reported data and participant sleep logs provided data on sleep period instead. Perceived sleep quality was assessed using the Pittsburgh Sleep Quality Index (PSQI) questionnaire at baseline and after the 12-week intervention, with total score range from 0 to 21 points and higher scores indicating worse sleep quality.^38^

Mood outcomes were assessed at baseline and after the 12-week intervention using self-reported questionnaires. Depression was assessed using the Beck Depression Inventory Fast Screen,^39^ with total score range from 0 to 21 points and higher scores reflecting more depressive symptoms. Anxiety was assessed using the State-Trait Anxiety Inventory,^40^ with total score range from 0 to 60 points for state anxiety and trait anxiety and higher scores reflecting a greater degree of anxiety. Perceived stress was assessed using the Perceived Stress Scale,^41^ with total score range from 0 to 56 points and a higher score indicating a greater degree of perceived stress.

Quality of life was assessed using the self-reported Rand 36-Item Short Form questionnaire (SF-36),^42^ with total score range from 0 to 100 points and higher scores indicating better quality of life. Further information about study assessments and end points is provided in eMethods 1 in Supplement 2.

### Study Intervention

All participants, including those in the UC group, logged their habitual eating window (first and last meal times) via the study’s mobile app during the 2-week lead-in period to confirm eligibility (eating window ≥12 hours). The UC group maintained their regular eating schedule (ie, eating window ≥12 hours) during the 12-week intervention and received an educational program comprising 1 in-person session every 2 weeks focused on weight management and cardiovascular health promotion, based on the Mediterranean dietary pattern^43^ and the World Health Organization’s physical activity recommendations.^44^ Participants in the early TRE group selected an 8-hour eating window starting no later than 10 am, while those in the late TRE group chose an 8-hour window starting no earlier than 1 pm. Participants in the self-selected TRE group established their preferred 8-hour eating window before the intervention. Participants in TRE groups were instructed to maintain the same eating window throughout the 12-week intervention and adhere to it every day of the week (ie, from Monday to Sunday). Caloric intake outside the eating window was prohibited for the TRE groups, although water, coffee, and tea without sugar or artificial sweeteners were allowed. Participants logged their first and last daily calorie intake via the study’s app during the 12-week intervention to monitor TRE adherence and that UC participants maintained their habitual daily eating window of 12 or more hours. Participants in TRE groups also received the same educational program as those in the UC group. Further details on the study intervention can be found elsewhere.^36^

### Statistical Analysis

Data for this secondary analysis were analyzed between March 14, 2024, and December 5, 2024. Sample size calculation is detailed elsewhere.^34^ Baseline characteristics were summarized as mean (SD) or frequency (percentage) by randomized groups unless otherwise stated. Intervention associations with sleep, mood, and quality-of-life outcomes at 3 months after the intervention were assessed based on repeated-measures linear mixed-effects multilevel models, including random cluster (site) effects.^45^ Individual measures of change were therefore modeled as the function of randomly assigned group, site, and assessment time and their interaction terms, considering sex as a covariate. Model-based estimations were performed with an intention-to-treat approach (including all participants as they were originally randomized) using the restricted maximum-likelihood method, the model assuming that missing values were missing at random. To account for multiple comparisons, we applied the Tukey correction within each outcome, adjusting for 6 pairwise comparisons among the 4 groups. All values presented in the tables are model-based estimates, including estimated mean differences with their Tukey-adjusted 95% CIs. The *P* values reported are Tukey-adjusted *P* values, and the threshold for significance was set at *P* < .05 (2-sided). Detailed information on the sensitivity analyses conducted is provided in eMethods 2 in Supplement 2. All statistical analyses were performed and figures created using R, version 4.4.2; linear mixed-effects models were performed using the lme4 package for R, version 1.1-36.

## Results

### Baseline Participant Characteristics

The screening process is detailed elsewhere and illustrated in eFigure 1 in Supplement 2. Table 1 displays descriptive data of the 197 study participants included in the present study and in the intention-to-treat analysis (mean [SD] age, 46.1 [8.4] years; mean [SD] body mass index, 32.8 [3.2]; 99 men [50.3%] and 98 women [49.7%]). Of note, 49 participants (25 men [51.0%], 24 women [49.0%]) were randomized to the UC group, 49 (25 men [51.0%], 24 women [49.0%]) to the early TRE group, 52 (27 men [51.9%], 25 women [48.1%]) to the late TRE group, and 47 (22 men [46.8%], 25 women [53.2%]) to the self-selected TRE group (Table 1 and eFigure 1 in Supplement 2). Overall, 14 participants (7.1%) were lost to the intervention end point (UC, 3 [21.4%]; early TRE, 2 [14.3%]; late TRE, 4 [28.6%]; self-selected TRE, 5 [35.7%]) due to various reasons, including work conflicts, lack of motivation, or unrelated health and personal issues. Sleep, mood, and quality-of-life end points at baseline and after the 12-week intervention in each group are displayed in eTable 1 in Supplement 2.

**Table 1.** Participant Baseline Characteristics According to Intervention Group

Characteristic	Participants (N = 197)^a^			
	UC (n = 49)	TRE		
		Early (n = 49)	Late (n = 52)	Self-selected (n = 47)
Age, y				
Mean (SD)	46.7 (6.0)	47.2 (6.2)	48.0 (6.9)	45.2 (5.8)
30-39	4 (8.2)	8 (16.3)	8 (15.4)	7 (14.9)
40-49	31 (63.3)	22 (44.9)	19 (36.5)	29 (61.7)
50-60	14 (28.6)	19 (38.8)	25 (48.1)	11 (23.4)
Sex				
Men	25 (51.0)	25 (51.0)	27 (51.9)	22 (46.8)
Women	24 (49.0)	24 (49.0)	25 (48.1)	25 (53.2)
Anthropometry and body composition				
Weight, mean (SD), kg	96.1 (14.4)	97.8 (15.3)	93.7 (15.4)	93.6 (13.8)
Height, mean (SD), cm	169.5 (9.1)	169.8 (9.4)	169.6 (9.6)	169.9 (8.6)
BMI, mean (SD)	33.4 (3.7)	33.8 (3.3)	32.4 (3.4)	32.4 (3.3)
Fat-free mass, mean (SD), kg	55.5 (11.0)	56.3 (11.8)	54.0 (11.5)	54.0 (10.7)
Fat mass				
Mean (SD), kg	39.7 (8.0)	40.4 (7.9)	39.0 (8.5)	38.7 (8.2)
Mean (SD), %	41.8 (6.7)	42.0 (6.3)	42.0 (7.1)	41.8 (7.1)
Medications^b^				
Sleep medication^c^	11 (22.4)	9 (18.4)	7 (13.5)	9 (19.1)
Antihypertensives	8 (16.3)	8 (16.3)	9 (17.3)	9 (19.1)
β-Blockers	1 (2.0)	0	1 (1.9)	0
Antidepressants	4 (8.2)	1 (2.0)	1 (1.9)	4 (8.5)
Anxiolytics	4 (8.2)	4 (8.2)	0	3 (6.4)
Statins	3 (6.1)	4 (8.2)	4 (7.7)	2 (4.3)
Sleep				
Onset, mean (SD), h:min	00:24 (00:54)	00:12 (00:48)	00:12 (00:48)	00:00 (00:48)
Offset, mean (SD), h:min	07:30 (00:48)	07:30 (01:00)	07:30 (00:48)	07:24 (00:48)
Period, mean (SD), h^d^	7.1 (0.9)	7.2 (1.0)	7.3 (0.7)	7.4 (0.7)
Total time, mean (SD), h^e^	6.2 (1.0)	6.3 (1.1)	6.4 (0.6)	6.4 (0.8)
Efficiency, mean (SD), %	86.8 (6.3)	86.4 (6.3)	87.6 (5.0)	86.4 (5.4)
Awakenings, mean (SD), No.	13.4 (3.5)	14.5 (3.7)	13.9 (3.1)	14.6 (3.1)
Wake after sleep onset, mean (SD), h	0.9 (0.4)	1.0 (0.4)	0.9 (0.4)	1.0 (0.4)
Sleep quality score, mean (SD)^f^	6.4 (3.0)	5.8 (2.7)	5.9 (2.8)	7.2 (3.1)
Mood				
Depression score, mean (SD)^g^	2.1 (2.4)	2.1 (2.2)	2.2 (2.5)	2.5 (2.6)
Minimal	39 (79.6)	40 (81.6)	42 (80.8)	35 (74.5)
Light	10 (20.4)	9 (18.4)	8 (15.4)	11 (23.4)
Moderate	0	0	2 (3.8)	1 (2.1)
Severe	0	0	0	0
State anxiety score, mean (SD)^h^	15.7 (11.1)	17.1 (11.3)	14.2 (9.1)	18.3 (9.9)
Low	37 (75.5)	31 (63.3)	39 (75.0)	33 (70.2)
Moderate	10 (20.4)	17 (34.7)	13 (25.0)	13 (27.7)
High	2 (4.1)	1 (2.0)	0	1 (2.1)
Trait anxiety score, mean (SD)^h^	17.5 (10.2)	19.1 (10.0)	17.5 (8.1)	21.1 (10.9)
Low	34 (69.4)	29 (59.2)	35 (67.3)	27 (57.4)
Moderate	13 (26.5)	20 (40.8)	16 (30.8)	17 (36.2)
High	2 (4.1)	0	1 (1.9)	3 (6.4)
Stress score, mean (SD)^i^	24.0 (8.5)	23.1 (8.6)	23.5 (8.1)	26.0 (6.9)
Quality-of-life scores, mean (SD)^j^				
Physical functioning	88.5 (11.7)	91.8 (6.8)	87.6 (10.9)	87.9 (13.6)
Role limitations				
Due to physical health	84.7 (32.6)	90.4 (23.6)	87.0 (25.0)	79.3 (35.5)
Due to emotional problems	90.5 (24.5)	83.7 (32.5)	94.2 (15.8)	74.5 (39.4)
Vitality	54.4 (18.9)	53.5 (19.3)	48.6 (16.0)	48.4 (14.6)
Social functioning	69.6 (22.1)	70.7 (23.8)	72.6 (23.6)	68.2 (23.9)
Bodily pain	79.8 (16.9)	82.1 (18.1)	78.0 (18.5)	69.3 (24.5)
General health	66.3 (16.4)	65.7 (17.1)	61.4 (16.2)	61.5 (17.6)

Abbreviations: BMI, body mass index (calculated as weight in kilograms divided by height in meters squared); TRE, time-restricted eating; UC, usual care.

^a^ Data are presented as number (percentage) of participants unless otherwise indicated.

^b^ Participants could take more than 1 medication.

^c^ Two participants (4.1%) in the UC group, 3 (6.1%) in the early TRE group, and 1 (2.1%) in the self-selected TRE group took prescribed sleep medication, while the remaining participants used over-the-counter options.

^d^ Time from sleep onset to sleep offset.

^e^ Amount of time classified as sleep within the sleep period.

^f^ Assessed using the Pittsburgh Sleep Quality Index (score range, 0-21 points, with higher scores indicating worse sleep quality).

^g^ Assessed using the Beck Depression Inventory Fast Screen (score range, 0-21 points, with higher scores reflecting more depressive symptoms). Depression severity is categorized based on the following scores: 0 to 3 (minimal), 4 to 8 (light), 9 to 12 (moderate), and 13 to 21 (severe).

^h^ Assessed using the State-Trait Anxiety Inventory (total score, 0-60 points for state anxiety and trait anxiety, with higher scores reflecting greater anxiety). State or trait anxiety severity is categorized based on the following scores: 0 to 20 (low), 21 to 40 (moderate), and 41 to 60 (high).

^i^ Assessed using the Perceived Stress Scale (score range, 0-56 points, with higher scores indicating more perceived stress).

^j^ Assessed using the Rand 36-Item Short Form Health Survey (score range, 0-100 points, with higher scores reflecting better quality of life).

### Sleep

**Figure 1.**
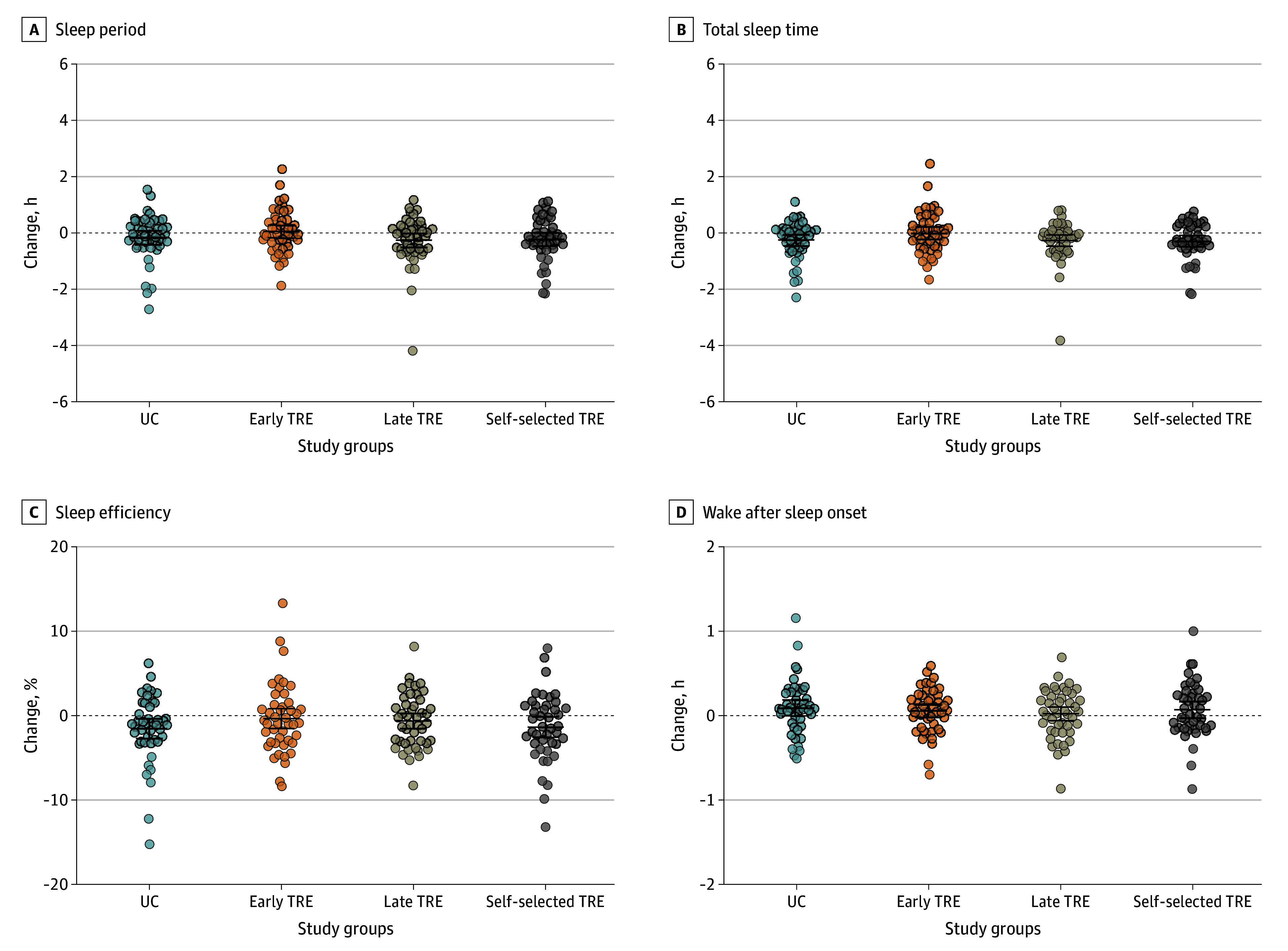
Changes in Sleep Outcomes Changes were measured by accelerometry over 14 days both at baseline and in the last 2 weeks of the 12-week intervention among the usual care (UC), early time-restricted eating (TRE), late TRE, and self-selected TRE groups. Changes were calculated as the difference between postintervention minus preintervention values. No statistically significant differences in changes in sleep outcomes were detected across all groups after the intervention. Circles represent individual participants’ measures; horizontal bars, raw means; whiskers, 95% CIs.

There were no statistically significant differences in changes in sleep onset, sleep offset, sleep period, total sleep time, sleep efficiency, number of awakenings, wake after sleep onset, and PSQI total score in the early TRE, late TRE, and self-selected TRE groups compared with the UC group (Figure 1 and Table 2) or between the TRE groups (Figure 1 and Table 3). Similar results were observed when daylight across the intervention period was included in the analyses (eTables 2-4 in Supplement 2). We repeated the analyses further adjusting for use of sleep medication, and the results remained unchanged. As a sensitivity analysis, we repeated the analyses after excluding participants who reported taking sleep medication at baseline, and the results remained consistent (eTables 5-7 in Supplement 2).There was no significant difference in the number of nights sleeping 7 hours or more in the early TRE group compared with the UC group (mean difference, 1.7 nights [95% CI, −0.2 to 3.5 nights]; *P* = .09) and the late TRE group (mean difference, 1.7 nights [95% CI, −0.1 to 3.6 nights]; *P* = .07); all other group comparisons showed no significant differences in this measure.

**Table 2.** Changes in Sleep, Mood, and Quality-of-Life End Points in the TRE Groups Compared With the UC Group After the 12-Week Intervention

End point	Difference, mean (95% CI)^a^		
	Early TRE vs UC	Late TRE vs UC	Self-selected TRE vs UC
Sleep			
Onset, h	−0.2 (−0.5 to 0.2)	0.0 (−0.4 to 0.4)	0.0 (−0.3 to 0.4)
Offset, h	0.0 (−0.4 to 0.4)	−0.2 (−0.6 to 0.3)	−0.1 (−0.5 to 0.4)
Period, h^b^	0.2 (−0.3 to 0.6)	−0.2 (−0.6 to 0.3)	−0.1 (−0.5 to 0.4)
Total time, h^c^	0.2 (−0.2 to 0.6)	−0.1 (−0.4 to 0.3)	0.0 (−0.4 to 0.4)
Efficiency, %	1.3 (−0.9 to 3.4)	1.0 (−1.2 to 3.1)	0.3 (−1.8 to 2.5)
Awakenings, No.	0.1 (−1.2 to 1.4)	−0.4 (−1.7 to 0.9)	−0.3 (−1.6 to 1.0)
Wake after sleep onset, h	−0.1 (−0.2 to 0.1)	−0.1 (−0.3 to 0.1)	0.0 (−0.2 to 0.1)
Sleep quality score^d^	0.4 (−1.1 to 1.9)	0.5 (−1.0 to 1.9)	−0.3 (−1.8 to 1.2)
Mood scores			
Depression^e^	0.2 (−1.0 to 1.3)	−0.1 (−1.2 to 1.0)	0.0 (−1.2 to 1.2)
State anxiety^f^	−1.2 (−6.4 to 4.1)	−1.8 (−6.9 to 3.3)	−0.6 (−6.0 to 4.7)
Trait anxiety^f^	0.4 (−3.4 to 4.1)	−0.9 (−4.5 to 2.8)	0.4 (−3.4 to 4.3)
Stress^g^	2.1 (−1.8 to 5.9)	0.3 (−3.5 to 4.0)	−0.1 (−4.0 to 3.8)
Quality-of-life scores^h^			
Physical functioning	−0.3 (−6.1 to 5.5)	3.1 (−2.6 to 8.7)	1.9 (−4.0 to 7.8)
Role limitations			
Due to physical health	−8.1 (−27.2 to 11.1)	3.8 (−14.8 to 22.5)	3.1 (−16.2 to 22.4)
Due to emotional problems	7.5 (−10.8 to 25.8)	7.5 (−10.3 to 25.3)	17.6 (−0.9 to 36.1)
Vitality	1.1 (−6.6 to 8.7)	6.1 (−1.4 to 13.5)	7.0 (−0.8 to 14.7)
Social functioning	−4.5 (−12.4 to 3.5)	−0.7 (−8.4 to 7.0)	−4.0 (−12.1 to 4.0)
Bodily pain	−2.7 (−15.1 to 9.7)	2.6 (−9.5 to 14.7)	10.5 (−2.1 to 23.0)
General health	3.3 (−4.4 to 10.9)	6.9 (−0.5 to 14.3)	2.8 (−4.9 to 10.5)

Abbreviations: TRE, time-restricted eating; UC, usual care.

^a^ Calculated by first computing the postintervention minus the preintervention values within each group; then, differences between the groups were computed as early TRE minus UC, late TRE minus UC, and self-selected TRE minus UC. Sample size: UC, n = 49; early TRE, n = 49; late TRE, n = 52; self-selected TRE, n = 47. No statistically significant differences were detected in changes in sleep, mood, and quality-of-life outcomes across all groups.

^b^ Time from sleep onset to sleep offset.

^c^ Amount of time classified as sleep within the sleep period.

^d^ Assessed using the Pittsburgh Sleep Quality Index (score range, 0-21 points, with higher scores indicating worse sleep quality).

^e^ Assessed using the Beck Depression Inventory Fast Screen (score range, 0-21 points, with higher scores reflecting more depressive symptoms).

^f^ Assessed using the State-Trait Anxiety Inventory (total score range, 0-60 points for state anxiety and trait anxiety, with higher scores reflecting greater anxiety).

^g^ Assessed using the Perceived Stress Scale (score range, 0-56 points, with higher scores indicating greater perceived stress).

^h^ Assessed using the Rand 36-Item Short Form Health Survey (score range, 0-100 points, with higher scores reflecting better quality of life).

**Table 3.** Changes in Sleep, Mood, and Quality-of-Life End Points in the TRE Groups Compared With Each Other After the 12-Week Intervention

End point	Difference, mean (95% CI)^a^		
	Early TRE vs late TRE	Early TRE vs self-selected TRE	Late TRE vs self-selected TRE
Sleep			
Onset, h	−0.2 (−0.7 to 0.4)	−0.1 (−0.7 to 0.4)	0.0 (−0.5 to 0.6)
Offset, h	0.2 (−0.4 to 0.9)	0.2 (−0.5 to 0.9)	0.0 (−0.7 to 0.7)
Period, h^b^	0.4 (−0.3 to 1.0)	0.3 (−0.3 to 1.0)	−0.1 (−0.7 to 0.6)
Total time, h^c^	0.3 (−0.3 to 0.9)	0.3 (−0.3 to 0.9)	0.0 (−0.6 to 0.6)
Efficiency, %	−0.2 (−3.2 to 2.8)	0.4 (−2.6 to 3.5)	0.6 (−2.4 to 3.7)
Awakenings, No.	0.3 (−1.4 to 2.0)	0.5 (−1.2 to 2.3)	0.2 (−1.5 to 1.9)
Wake after sleep onset, h	0.1 (−0.2 to 0.3)	0.1 (−0.2 to 0.3)	0.0 (−0.3 to 0.2)
Sleep quality score^d^	0.8 (−0.8 to 2.5)	0.9 (−0.8 to 2.7)	0.1 (−1.6 to 1.8)
Mood scores			
Depression^e^	0.3 (−0.8 to 1.4)	0.2 (−1.0 to 1.3)	−0.1 (−1.2 to 1.0)
State anxiety^f^	0.6 (−4.4 to 5.6)	−0.5 (−5.8 to 4.7)	−1.2 (−6.3 to 3.9)
Trait anxiety^f^	1.3 (−2.3 to 4.9)	−0.1 (−3.8 to 3.7)	−1.3 (−5.0 to 2.3)
Stress^g^	1.8 (−1.8 to 5.5)	2.2 (−1.6 to 6.1)	0.4 (−3.3 to 4.1)
Quality-of-life scores^h^			
Physical functioning	−3.4 (−9.0 to 2.2)	−2.2 (−8.0 to 3.6)	1.2 (−4.5 to 6.8)
Role limitations			
Due to physical health	−11.9 (−30.4 to 6.6)	−11.2 (−30.4 to 8.0)	0.7 (−18.0 to 19.4)
Due to emotional problems	0.0 (−17.7 to 17.7)	−10.1 (−28.4 to 8.3)	−10.1 (−28.0 to 7.8)
Vitality	−5.0 (−12.4 to 2.4)	−5.9 (−13.6 to 1.8)	−0.9 (−8.3 to 6.6)
Social functioning	−3.8 (−11.4 to 3.9)	−0.4 (−8.4 to 7.5)	3.4 (−4.3 to 11.0)
Bodily pain	−5.3 (−17.3 to 6.7)	−13.2 (−25.6 to −0.7)^i^	−7.9 (−20.0 to 4.2)
General health	−3.6 (−11.0 to 3.7)	0.5 (−7.2 to 8.1)	4.1 (−3.4 to 11.5)

Abbreviation: TRE, time-restricted eating.

^a^ Calculated by first computing the postintervention minus the preintervention values within each group; then, differences between the groups were computed as early TRE minus late TRE, early TRE minus self-selected TRE, and late TRE minus self-selected TRE. Sample size: early TRE, n = 49; late TRE, n = 52; self-selected TRE, n = 47.

^b^ Time from sleep onset to sleep offset.

^c^ Amount of time classified as sleep within the sleep period.

^d^ Assessed using the Pittsburgh Sleep Quality Index (score range, 0-21 points, with higher scores indicating worse sleep quality).

^e^ Assessed using the Beck Depression Inventory Fast Screen (score range, 0-21 points, with higher scores reflecting more depressive symptoms).

^f^ Assessed using the State-Trait Anxiety Inventory (total score, 0-60 points for state anxiety and trait anxiety, with higher scores reflecting greater anxiety).

^g^ Assessed using the Perceived Stress Scale (score range, 0-56 points, with higher scores indicating greater perceived stress).

^h^ Assessed using the Rand 36-Item Short Form Health Survey (score range, 0-100 points, with higher scores reflecting better quality of life).

^i^ Significant difference between TRE groups, as determined by post hoc Tukey correction for multiple comparisons (*P* < .05).

### Mood

**Figure 2.**
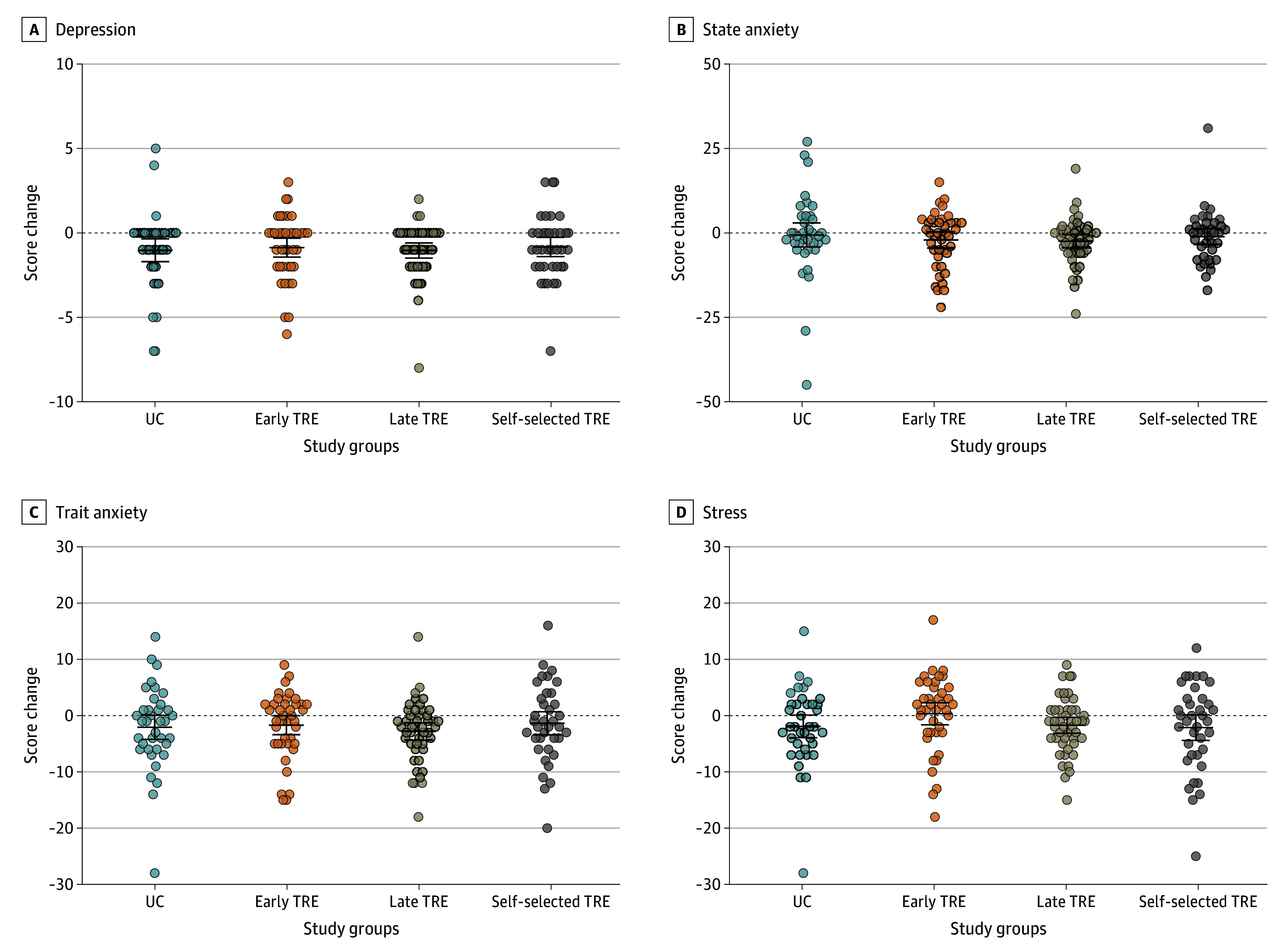
Changes in Mood Outcomes Changes were calculated as postintervention minus preintervention values. No statistically significant differences in changes in mood outcomes were detected across all groups after the intervention. Depression was assessed using the Beck Depression Inventory Fast Screen (score range, 0-21 points, with higher scores reflecting more depressive symptoms). State anxiety and trait anxiety were assessed using the State-Trait Anxiety Inventory (score range, 0-60 points, with higher scores reflecting greater anxiety). Stress was assessed using the Perceived Stress Scale (score range, 0-56 points, with higher scores indicating more stress symptoms). TRE indicates time-restricted eating; UC, usual care. Circles represent individual participants’ scores; horizontal bars, raw means; whiskers, 95% CIs.

No significant differences were observed between the early TRE and UC groups for mood (eg, mean difference in Beck Depression Inventory Fast Screen score, 0.2 [95% CI, –1.0 to 1.3] points; mean difference in state anxiety score on the State-Trait Anxiety Inventory, –1.2 [95% CI, –6.4 to 4.1] points; mean difference in Perceived Stress Scale score, 2.1 [95% CI, –1.8 to 5.9] points) (Figure 2 and Table 2). There also were no significant differences between the TRE groups (Figure 2 and Table 3).

### Quality of Life

No statistically significant differences were found in changes in overall health markers in the early TRE, late TRE, and self-selected TRE groups compared with the UC group (eg, mean difference in SF-36 general health score for early TRE vs UC, 3.3 [95% CI, −4.4 to 10.9] points; late TRE vs UC, 6.9 [95% CI, −0.5 to 14.3] points; self-selected TRE vs UC, 2.8 [95% CI, −4.9 to 10.5] points) (Table 2 and eFigure 2 in Supplement 2). However, bodily pain was significantly reduced in the early TRE group compared with the self-selected TRE group (mean difference in SF-36 score, −13.2 [95% CI, −25.6 to −0.7]; *P* = .03) (Table 3). No other statistically significant differences among TRE groups were observed (Table 3 and eFigure 2 in Supplement 2).

### Eating Window Times

As reported before,^34^ the eating window was significantly shorter in the early TRE group (median, 7.7 [IQR, 7.4-7.8] hours; *P* < .001), the late TRE group (median, 7.4 [IQR, 7.0-7.8] hours; *P* < .001), and the self-selected TRE group (median, 7.6 [IQR, 7.3-7.9] hours; *P* < .001) compared with the UC group (median, 13.4 [IQR, 12.6-14.0] hours). Median eating window times were 8:30 am (IQR, 7:35-9:30 am) to 10 pm (IQR, 9:30-10:25 pm) for the UC group, 9:45 am (IQR, 9:25-10:10 am) to 5:30 pm (IQR, 5:00-5:55 pm) for the early TRE group, 2:20 pm (IQR, 1:30-3:00 pm) to 9:30 pm (IQR, 9:00-10:20 pm) for the late TRE group, and 12:20 pm (IQR, 11:00 am to 1:40 pm) to 8 pm (IQR, 6:40-9:10 pm) for the self-selected TRE group.

### Association of the Intervention With Sleep, Mood, and Quality of Life by Sex

Results remained similar when repeating all analyses by sex. Data are shown in eTables 8 to 16 and eFigures 3 to 5 in Supplement 2.

## Discussion

Findings from the present secondary analysis suggest that incorporating TRE into the UC intervention, regardless of the timing of the eating window, did not lead to additional changes in sleep, mood, or quality of life compared with UC alone (eating schedule ≥12 hours) in men and women with overweight or obesity. Although women are commonly more prone to sleep disturbances, depression, anxiety, stress, and lower quality of life than men,^27,28,29,30^ the TRE intervention did not appear to influence these variables across any of the TRE groups in our study. We found that TRE, irrespective of the eating window timing, was not associated with adverse effects on sleep, mood, or quality of life in adults with overweight or obesity, suggesting it may be a safe weight-management nutritional strategy. Further research is needed to explore the broader health effects of different TRE schedules, particularly in populations with specific sleep disturbances (eg, shift workers) or mood disorders (eg, depression, anxiety, and stress) as well as in individuals with different chronotypes and those with chronic diseases, where metabolic and circadian factors may play a distinct role.

Our results align with previous research on the effect of different TRE schedules on sleep^19,21,25,46^ and concur with a recently published 6-month TRE trial in adults with type 2 diabetes.^35^ In brief, that RCT found that mid-to-late TRE (ie, 12 pm to 8 pm) did not lead to statistically significant differences in sleep duration or quality, as assessed using the PSQI, compared with both a caloric restriction group (25% energy restriction daily) and a control group instructed to maintain usual eating and daily routines.^35^ Similarly, an exploratory RCT found no significant differences in sleep quality changes, as assessed by the PSQI, between early TRE (eating window from 8 am to 4 pm), late TRE (12 pm to 8 pm), either early or late TRE combined with caloric restriction, and a caloric restriction–only group (eating window from 8 am to 8 pm) over an 8-week intervention in adults with overweight or obesity.^24^ A 4-week pilot trial conducted in our laboratory compared the effects of early, late, and self-selected TRE on sleep quality, as assessed by the PSQI, and found no significant differences across TRE groups in a sample of 22 adults with overweight or obesity.^47^ While it could be hypothesized that earlier eating windows may improve sleep by advancing sleep timing^48^ and better alignment of meal timing with circadian rhythms (thereby reducing melatonin-insulin interference, enhancing nighttime glucose regulation, and promoting satiety),^16,49^ the current evidence is inconclusive. In this study, there was no significant difference in the number of nights sleeping 7 hours or more, which is a marker of improved sleep health,^50^ in the early TRE group compared with the UC group and the late TRE group; however, while the differences between groups were not statistically significant, the observed pattern may warrant further investigation in future studies exploring the potential effects of early TRE on sleep health. Recent systematic reviews have either found no consistent association of early or late TRE with sleep or reported a lack of consensus, due to the limited number of studies investigating the mechanisms by which TRE may influence sleep and the significant variability in study designs.^17,22^ Although our team observed that the early TRE group exhibited reductions of approximately 6 mg/dL in fasting glucose and 9 mg/dL in nocturnal glucose levels compared with the UC, late TRE, and self-selected TRE groups,^34^ melatonin-insulin interference was not assessed, which may further elucidate the role of insulin and glycemic regulation in sleep outcomes. Of note is that participants in the trial did not receive sleep hygiene counseling and were allowed to consume noncaloric caffeinated beverages during the fasting windows, which could have influenced results. Therefore, future TRE trials incorporating behavioral interventions to improve sleep are warranted.

Regarding mood, we observed that a 12-week TRE intervention, irrespective of the eating window timing, was not associated with significant changes in depression, anxiety, or stress compared with UC in men and women with overweight or obesity. Our findings align with current literature, indicating that TRE seems to be a valid nutritional strategy for obesity management without apparent adverse effects on mood in adults with obesity.^21,51,52,53^ Similarly, the 12-week TRE intervention, regardless of the eating window timing, was not associated with additional changes in quality of life compared with UC in men and women with overweight or obesity. A longer 12-month RCT comparing an 8-hour TRE window (ie, 12 pm to 8 pm) with a caloric restriction group (25% energy restriction daily) and a control group that was instructed to maintain their usual eating and daily habits found no significant differences between groups in quality of life.^52^ Thus, it can be suggested that a TRE intervention may not provide superior benefits to or detrimental effects on quality of life compared with other nutrition-based obesity management strategies, such as caloric restriction or Mediterranean dietary pattern–based education programs. Although the TRE groups achieved greater body weight loss than the UC group,^34^ the amount lost may have been insufficient to elicit significant changes in sleep, mood, and quality of life. Additionally, the duration of the intervention might have been too short to detect such differences.^4^ Further, longer trials are required to confirm these findings.

### Limitations

This secondary analysis of an RCT has several limitations. First, the power calculation of sample size was based on visceral adipose tissue changes,^34^ the main outcome of the overall project. Thus, the current study may not be well powered to detect small but significant changes in sleep, mood, or quality of life. Second, study duration may limit the translation of the present findings to detect significant differences among the intervention groups. Third, participants could consume caffeinated beverages during the fasting windows, which was not controlled during the study period and may have influenced sleep. Fourth, chronotype was not accounted for in the analyses and may have influenced our results. However, as participants reported similar bedtimes and wake-up times across groups before and during the 12-week intervention, the potential impact of chronotype on our findings may have been minimized. Participants had only mildly impaired sleep quality at baseline; therefore, whether early, late, or self-selected TRE could benefit individuals with more severe sleep disturbances remains unknown. In addition, we have no information about the napping behavior during the intervention, as participants did not keep napping diaries and were instructed only to register bedtime and wake-up time in the mobile phone application. Nevertheless, self-reported nap data are often subject to recall bias and inconsistencies.

## Conclusions

In this secondary analysis of an RCT of 3 different TRE schedules, the findings suggest that incorporating TRE into a UC intervention, regardless of the timing of the eating window, did not lead to significant changes in sleep, mood, or quality of life compared with UC alone (Mediterranean diet education program) in men and women with overweight or obesity. Notably, even though women tend to experience more sleep disturbances, depression, anxiety, stress, and lower quality of life than men,^27,28,29,30^ the TRE intervention was not associated with adverse effects on these health-related outcomes in women. Therefore, TRE appeared to be a well-tolerated nutritional strategy for managing body weight without apparent adverse effects on overall sleep health and psychological well-being in both men and women regardless of the fasting-eating window implemented. Further trials should incorporate polysomnography to assess whether different TRE schedules may influence additional objective sleep parameters, such as sleep architecture (ie, time spent in different sleep stages) and sleep continuity (ie, sleep depth and fragmentation).

## Notes

Supplement 1.
Trial Protocol

Supplement 2. **eMethods 1.** Study Assessments and End Points **eMethods 2.** Sensitivity Analyses **eTable 1.** Sleep, Mood, and Quality-of-Life End Points at Baseline and After the 12-Week Intervention in Each Intervention Group **eTable 2.** Changes in Sleep End Points in the Time-Restricted Eating Groups Compared With the Usual Care Group After the 12-Week Intervention Considering the Daylight Time in the Analysis **eTable 3.** Changes in Sleep End Points in the Time-Restricted Eating Groups Compared With Each Other After the 12-Week Intervention Considering the Daylight Time in the Analysis **eTable 4.** Sleep End Points at Baseline and After the 12-Week Intervention in Each Intervention Group Considering the Daylight Time in the Analysis **eTable 5.** Changes in Sleep End Points in the Time-Restricted Eating Groups Compared With the Usual Care Group Following the 12-Week Intervention, After Excluding Participants Who Reported Taking Sleep Medication at Baseline **eTable 6.** Changes in Sleep End Points in the Time-Restricted Eating Groups Compared With Each Other Following the 12-Week Intervention, After Excluding Participants Who Reported Taking Sleep Medication at Baseline **eTable 7.** Sleep End Points at Baseline and Following the 12-Week Intervention in Each Intervention Group, After Excluding Participants Who Reported Taking Sleep Medication at Baseline **eFigure 1.** Study Flowchart **eFigure 2.** Changes in General Health Across All Groups After the 12-Week Intervention **eTable 8.** Baseline Characteristics of All Randomized Participants by Sex and Intervention Group **eTable 9.** Changes in Sleep, Mood, and Quality-of-Life End Points in the Time-Restricted Eating Groups Compared With the Usual Care Group After the 12-Week Intervention, Divided by Sex **eTable 10.** Changes in Sleep, Mood, and Quality-of-Life End Points in the Time-Restricted Eating Groups Compared With Each Other After the 12-Week Intervention, Divided by Sex **eFigure 3.** Changes in Sleep Outcomes Across All Groups After the 12-Week Intervention, Divided by Sex **eFigure 4.** Changes in Mood Outcomes Across All Groups After the 12-Week Intervention, Divided by Sex **eFigure 5.** Changes in General Health Across All Groups After the 12-Week Intervention, Divided by Sex **eTable 11.** Sleep, Mood, and Quality-of-Life End Points at Baseline and After the 12-Week Intervention in Each Intervention Group in Men **eTable 12.** Sleep, Mood, and Quality-of-Life End Points at Baseline and After the 12-Week Intervention in Each Intervention Group in Women **eTable 13.** Changes in Sleep End Points in the Time-Restricted Eating Groups Compared With the Usual Care Group After the 12-Week Intervention Considering the Daylight Time in the Analysis, Divided by Sex **eTable 14.** Changes in Sleep End Points in the Time-Restricted Eating Groups Compared With Each Other After the 12-Week Intervention Considering the Daylight Time in the Analysis, Divided by Sex **eTable 15.** Sleep End Points at Baseline and After the 12-Week Intervention Considering the Daylight Time in the Analysis in Men **eTable 16.** Sleep End Points at Baseline and After the 12-Week Intervention Considering the Daylight Time in the Analysis in Women
eReferences

Supplement 3. **eTable 17.** GGIR Configuration for Accelerometry Data Processing

Supplement 4.
Data Sharing Statement
