# Supplementary material for: Time-Restricted Eating and Sleep, Mood, and Quality of Life in Adults With Overweight or Obesity: A Secondary Analysis of a Randomized Clinical Trial
Source: JAMA Netw Open. 2025 Jun 25;8(6):e2517268. doi: 10.1001/jamanetworkopen.2025.17268 (PMC12199060; doi:10.1001/jamanetworkopen.2025.17268)
Supplement: Supplement 1. — Trial Protocol [file jamanetwopen-e2517268-s001.pdf]

Effects of early, late and self-selected time-restricted eating on visceral adipose tissue and cardiometabolic health in men and women with overweight/obesity: A multicenter randomized controlled trial

Trial Protocol and Statistical Analysis Plan

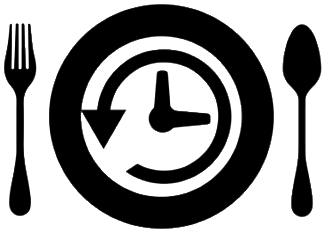

Contact details:

- Jonatan Ruiz Ruiz, Department of Physical and Sports Education, Faculty of Sports Science, University of Granada, Carretera de Alfacar s/n, 18071 Granada, Spain. E-mail: ruizj@ugr.es
- Idoia Labayen, Department of Health Sciences, Public University of Navarra, Campus de Arrosadia, Pamplona, Spain. Email: idoia.labayen@unavarra.es

INDEX

1. BACKGROUND AND RATIONALE 3
2. AIMS 5
3. METHODS/DESIGN 5
3.1 Study design 5
3.3 Recruitment and screening 8
3.4 Lead-in period 8
3.5 Measurements 8
3.6 Primary outcome: Visceral adipose tissue 9
3.7 Secondary outcomes 9
3.8 Time-restricted eating intervention 13
3.9 Usual-care intervention 14
3.10 Assessment of adherence and adverse events 14
3.11 Participants retention 14
3.12 Feasibility 15
3.13 Data management 16
4. STATISCAL ANALYSIS PLAN 16
4.1 Randomization and blinding 16
4.2 Sample size 17
4.3 Statistical analysis 18
5. ETHICAL ISSUES 18
5.1 Institutional Ethics Committee Approval 18
5.2 Patient Consent 19
5.3 Confidentiality and Privacy 19
5. REFERENCES 20
6. SUMMARY OF CHANGES TO STUDY PROTOCOL 25

1. BACKGROUND AND RATIONALE

Obesity has nearly tripled since 1975 and currently there are more than 1.9 billion adults with overweight or obesity worldwide according to the World Health Organization [1]. The obesity epidemic is a major contributor to the global burden of disability and chronic disease such as diabetes, cardiovascular disease, certain cancers, kidney disease, and obstructive sleep apnea [1, 2]. Consequentially, obesity results in a significant economic impact on health care systems [1, 3].

Energy restricted diets reduce body weight and improve cardiometabolic health. However, these approaches are still not a standard public health strategy owing to their limited long-term adherence, even in highly motivated patients. Over the past decade, intermittent fasting has emerged as a promising dietary strategy for the treatment of obesity and its comorbidities [4-6]. In simple terms, intermittent fasting consists of alternating consistent fasting and eating periods [4-6]. Intermittent fasting is an umbrella term for several protocols of fasting regimens [5, 6]. Among the various types of intermittent fasting, time-restricted eating (TRE) has recently received increasing attention from researchers since it seems to be a safe and feasible fasting regimen for most people [5, 6]. TRE consists of restricting the daily energy intake to a pre-determined eating window (generally ≤10 hours) and fasting for the rest of the day (14 hours or more) [5, 6]. During the eating window, individuals are not required to count calories or monitor food intake in any way [5, 6].

Human trial findings indicate that TRE leads to a reduction in body weight by 1–4% in the short term (i.e., ≤3 months) in individuals with overweight/obesity relative to controls with no energy and meal timing restrictions [5, 6]. This weight loss results from unintentional reductions in energy intake (10-30% or ~300-500 kcal/day) that occurs when participants confine their eating windows to 4-10 hours/day [5, 6]. Nonetheless, the impact of TRE on cardiometabolic risk parameters remains uncertain, and while some studies have demonstrated improvements in blood pressure, insulin resistance, lipid profile and markers of oxidative stress, others have shown no benefit on these parameters [5-7]. Moreover, there are still important questions regarding the effects of TRE that need to be addressed.

One such question is its impact on ectopic fat deposition [5], specifically visceral adipose tissue (VAT) [8, 9], which is known as a risk factor for cardiometabolic morbidity and mortality [10]. It is worth noting that there are sex differences in fat distribution, with women tending to accumulate fat subcutaneously in the gluteofemoral region, while men have a higher propensity for visceral fat accumulation [10]. However, despite the evidence indicating sex disparities in fat accumulation [10] and obesity-related comorbidities [11], and a growing emphasis on recognizing sex as a biological variable in research [12], there remains a scarcity of evidence regarding the effects of TRE interventions in men and women separately. This lack of evidence is primarily due to studies not being adequately powered to conduct separate analysis for each sex, leading to the presentation of results combined for both sexes.

Another crucial inquiry surrounding TRE pertains to the impact of eating window timing on its efficacy [5]. Most studies of TRE have used an arbitrary clock time to characterize the timing of food intake (e.g., 12:00-20:00). However, this approach does not consider meal timing in relation to the internal circadian time. The body is biologically primed for food intake during the mid-morning, when melatonin levels are decreased, and cortisol leves, insulin sensitivity, beta cell responsiveness, and thermic effect of food are at their peak [13]. Therefore, it has been hypothesized that earlier eating windows during TRE may induce superior metabolic benefits than later eating windows. Indeed, it has been shown that early TRE (6h eating window, with last meal before 15:00) enhanced cardiometabolic health compared with controls (12h eating window) after 5 weeks even in the absence of weight loss [14]. Recently, a meta-analysis has shown that early TRE was more effective in improving insulin resistance compared to late TRE [15]. Additionally, only early TRE demonstrated significant benefits in fasting blood glucose and diastolic blood pressure compared to controls (unrestricted eating time) [15]. Nevertheless, no significant differences between early and late TRE were found for body weight loss, fasting blood glucose, blood pressure, and lipid profile [15]. Furthermore, previous studies present important limitations, such as short duration (≤8 weeks), lack of randomization, small sample size to analyze and understand the effects in men and women separately, and the absence of a self-selected TRE group [16-19].

Taking an individual's schedule and personal preference into consideration, and allowing participants to choose their own TRE window, may be important factors to improve adherence, acceptability, and resulting efficacy. Therefore, it is still unclear whether there are differences in the effectiveness of early and late TRE on body weight and cardiometabolic risk factors, or if the effectiveness is solely dependent on the duration of the eating window, regardless of the timing.

2. AIMS

The overall aim of the present study is to investigate the efficacy and feasibility of three different 8h TRE schedules (i.e., early, late, and self-selected) compared to each other and to a usual-care (UC) intervention over 12 weeks on VAT, body composition and cardiometabolic health in adults with overweight/obesity.

3. METHODS/DESIGN

3.1 Study design

The experimental protocol was approved by the Servicio Andaluz de Salud (Comité Ético de Investigación Provincial de Granada) and the Comité Ético de Investigación Clínica de Navarra (PI_2021/119). Participants will provide written informed consent prior to study participation (see Online Supporting Information). The present study is registered at the US National Institutes of Health (ClinicalTrials.gov), identifier: NCT05310721. Adults with overweight/obesity will be recruited in Granada (southern of Spain) and Pamplona (northern of Spain). This study will therefore be a multicenter randomized controlled trial, which will certainly increase the generalizability and external validity of results by providing a broader basis for generalizations across institutions. The study design is illustrated in **Figure 1**.

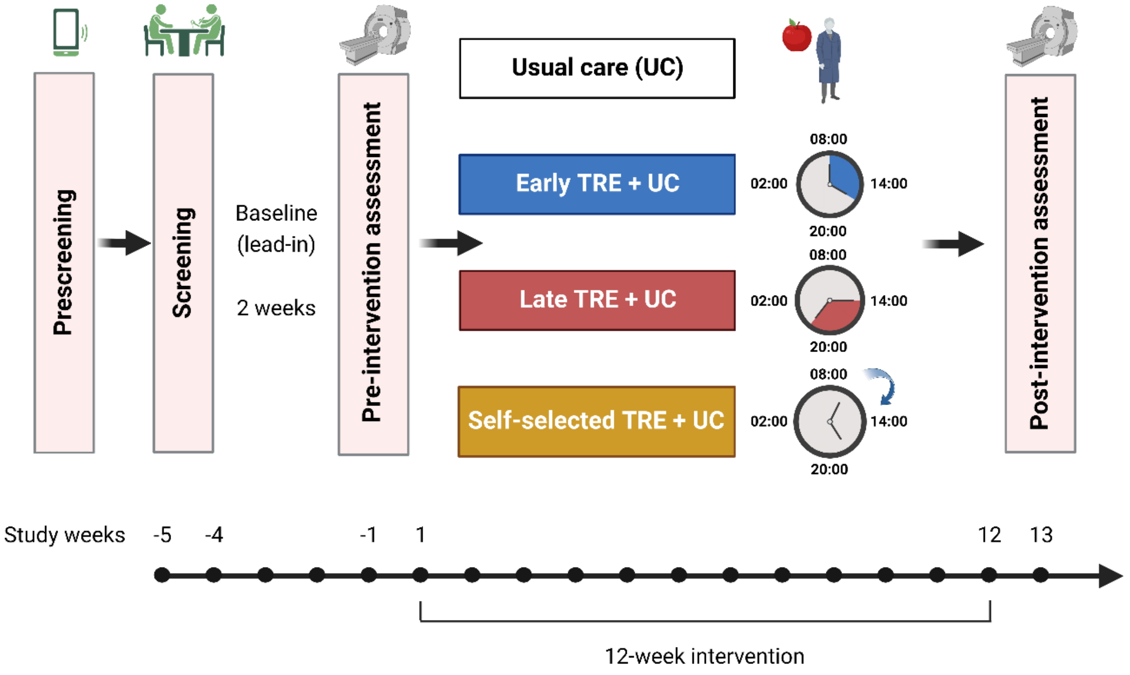

Figure 1. Study design. Prescreening of potential participants will be performed by phone calls, and potential participant will be scheduled for a screening visit. The randomized controlled trial includes a 2-week baseline (lead-in period) and 12-week dietary intervention. TRE, time-restricted eating.

3.2 Participants and eligibility criteria

The study population will be men and women (50%) with overweight/obesity and with at least one impaired cardiometabolic risk factor. The inclusion and exclusion criteria are listed in **Table 1**. Eligibility of study participants will be based on the results of screening medical history, vital signs, and clinical laboratory tests (Screening phase, **Figure 1**). The nature of any conditions present at the time of the physical examination and any pre-existing conditions will be documented.

Table 1. Eligibility criteria of the study.

Inclusion criteria	Exclusion criteria
Aged 30-60 years.<br><br>Body mass index ≥25.0 and <40 kg/m^2 and abdominal obesity (waist circumference ≥95 cm in men and ≥82 cm in women).<br><br>Weight stability (within 3% of screening weight) for >3 months prior to study entry.<br><br>Sedentary lifestyle (<150 min/week of moderate-vigorous intensity exercise) for >3 months prior to study entry.<br><br>Habitual eating window ≥12 hours.<br><br>At least one of the following metabolic impairments:<br><br>• High-density lipoprotein (HDL) cholesterol concentration <50 mg/dL for females and <40 mg/dL for males.<br>• Low-density lipoprotein (LDL) cholesterol levels >100 mg/dL (or on medication to treat elevated LDL cholesterol levels).<br>• Serum triglycerides concentration ≥150 mg/dL or on medication to treat elevated triglycerides.<br>• Systolic blood pressure >130 mm Hg and/or diastolic blood pressure >85 mm Hg or already being treated with anti-hypertension medications.<br>• Impaired glucose tolerance defined as at least one of the following:<br>o Fasting plasma glucose (FPG) ≥100 mg/dL and ≤125 mg/dL.<br>o Haemoglobin A1c (HbA1c) between ≥5.7% and <6.5%.<br>o Insulin resistance as determined by the Homeostatic Model Assessment of Insulin Resistance (HOMA-IR) >1.8.	• History of a major adverse cardiovascular event (acute myocardial infarction, ischemic or haemorrhagic stroke, peripheral arterial ischemia), kidney failure, chronic liver disease, or HIV / AIDS. Active endocrinological disease (Cushing's syndrome, Acromegaly, Adrenocortical insufficiency, GH deficiency), Innate errors of metabolism, Myopathies, Epilepsy. Patients who have undergone bariatric surgery surgical techniques or used for the treatment of other pathologies (Example: "Roux Y").<br>• Rheumatoid arthritis, Parkinson's disease, active cancer treatment in the past year, type 1 or 2 diabetes mellitus, or another medical condition where fasting is contraindicated.<br>• Use of medications that may affect the results of the study; for example, drugs for glycaemic control (e.g., antidiabetic, steroids, beta-blockers, antibiotics, prebiotics, probiotics and symbiotics).<br>• Metal or electrical prosthesis.<br>• Foreign bodies in the eyes.<br>• Diagnosis of major sleep or eating disorders.<br>• Active tobacco or illicit drug use or a history of alcohol abuse treatment (this is moderate or severe alcoholism).<br>• Participating in a weight loss or a weight-management program.<br>• Pregnancy and lactation or planned pregnancy (within the study period).<br>• Caregiver for a dependent requiring frequent nocturnal care/sleep interruption.<br>• Shift workers with nocturnal hours.<br>• Frequent travel over time zones during the study period.<br>• Fear of needles and claustrophobia to magnetic resonance imaging (MRI).<br>• Being unable to understand and to accept the instructions or the study objectives and protocol.<br>• Not having or being able to use a smartphone with Apple iOS or Android OS.<br>• Are deemed unsuitable by the investigator for any other reason.

3.3 Recruitment and screening

In Granada, potential participants will be recruited through advertisements on newspapers and the Endocrinology and Nutrition service of the Hospital Universitario Clínico San Cecilio and Virgen de las Nieves. In Pamplona, potential participants will be recruited through advertisements on newspapers and in the Endocrinology and Nutrition service of the University Hospital of Navarra. A pre-screening telephone interview will be performed to determine the eligibility of potential participants and check their interest in the study (**Figure 1**). Potential participants who will be eligible based on the pre-screening will receive written information about the study and will be scheduled for a screening visit (**Figure 1**). At the screening visit, potential participants will provide oral and written informed consent; in addition, body weight, anthropometry, and blood pressure will be measured. Afterwards, the endocrinologists, by telephone interview and consulting the patient medical records, will assess the medical history and check inclusion and exclusion criteria (**Table 1**). Subsequently, potential participants will be scheduled to have fasting blood sampling to confirm that they present at least one of the metabolic impairments (see inclusion criteria, **Table 1**).

3.4 Lead-in period

There will be a 2-week lead-in period (before baseline measurements and group allocation; **Figure 1**) in which potential participants will continue with their habitual nutritional and physical activity habits. Potential participants will record daily their eating time and sleep and any adverse events using a mobile phone app (EXTREME: com.nnbi.app_extreme, NNBi2020 S.L., Navarra, Spain). This data will be used to confirm that their eating window is ≥12 hours (see inclusion criteria, **Table 1**).

3.5 Measurements

All measurements will be conducted both at baseline and 12 weeks post-intervention (± 3 days) by experienced and trained personnel. Standardization of dietary intake, hydration, and physical activity will be implemented prior to the measurements of ectopic fat depots, body composition, and cardiometabolic risk factors. Specifically, participants will be instructed to observe a fasting period of 10 to 12 hours before their appointment, during which they will be advised to consume only water and abstain from solid food or other beverages.

It will be emphasized that they should have a complete meal at least 10 hours before the test. Furthermore, participants will be advised not to consume alcohol, drugs or diuretics within 24 hours before the test, and to avoid stimulants like caffeine or theine for 12 hours prior to the test. Lastly, participants will be instructed to refrain from engaging in moderate exercise or physical activity for 24 hours, and from vigorous exercise for 48 hours, prior to the test. For a comprehensive summary of the study's outcomes, refer to Table 2.

3.6 Primary outcome: Visceral adipose tissue

VAT (primary outcome) will be quantified by magnetic resonance imaging (Siemens 3T Magnetom Vida in both Granada and Pamplona).

3.7 Secondary outcomes

Body weight, anthropometry, and body composition

Body weight and height will be measured using a stadiometer and scale (Seca model 799, Electronic Column Scale, Hamburg, Germany in both Granada and Pamplona) without shoes and with light clothing. Neck, waist, and hip circumferences will be measured following the ISAK procedures [20]. Bone mineral density, fat mass, and fat-free mass will be assessed using a dual-energy X-ray absorptiometry scan (QDR Discovery Wi Hologic, Inc., Bedford, MA, USA in Granada and Horizon Wi Hologic, Inc., Bedford, MA, USA in Pamplona).

Ectopic fat depots

Abdominal subcutaneous and intermuscular adipose tissue, as well as hepatic fat fraction will be quantified by magnetic resonance imaging (Siemens 3T Magnetom Vida). Semiautomatic software for tissue segmentation will be used to calculate visceral, subcutaneous, and intermuscular abdominal adipose tissue variables in all 3D abdominal volume: volume, cross-sectional area at selected levels, and mean/median fat fraction. These image markers will be derived from a standard 6 echo Dixon series. At L3 and L5 levels, we will obtain these image markers: cross-sectional area, muscular tissue, and intramuscular fat fraction. We will also quantify with a 6 echoes Dixon series, the fat fraction in all lumbar vertebral bodies, from a mid-sagittal image.

The segmentation of these areas will be manually edited. Lastly, we will measure image markers from mid-thigh. Again, using a 6 echoes Dixon series, we will obtain cross-sectional area, muscular tissue, intermuscular adipose tissue, fat fraction, subcutaneous adipose tissue, and bone marrow fat fraction. The segmentation for all these structures will be done with a semiautomatic proprietary algorithm.

Cardiometabolic risk factors

Fasting blood samples will be collected to analyze:

- Glucose (AU5800 automated analyzer, Beckman Coulter Inc., CA, USA), insulin (UniCel DxI 800 access immunoassay system, Beckman Coulter Inc., CA, USA), and HbA1c (HA-8180V® analyzer, A Menarini Diagnostics, Firenze, Italy). Simple insulin resistance surrogates such as the HOMA-IR and the quantitative insulin-sensitivity check index (QUICKI) will be calculated.
- Lipid profile, including total cholesterol, high-density lipoprotein cholesterol, triglycerides, apolipoprotein A1 and B, will be measured using a AU5800 automated analyzer, Beckman Coulter Inc., CA, USA. Low-density lipoprotein cholesterol will be calculated using a previously validated equation [21].
- Bone metabolism profile, including Vitamin D (measured using a UniCel DxI 800 access immunoassay system, Beckman Coulter Inc., CA, USA), alkaline phosphatase (measured by Liaison, Diasorin, Saluggia VC, Italy), and calcium (measured using a AU5800 automated analyzer, Beckman Coulter Inc., CA, USA).
- Liver and kidney function markers, including alanine transaminase, gamma-glutamyl transferase, bilirubin, creatinine (measured using a AU5800 automated analyzer, Beckman Coulter Inc., CA, USA), and estimated glomerular filtration rate.
- Steroid hormones, including estradiol, progesterone, and testosterone (measured using a UniCel DxI 800 access immunoassay system, Beckman Coulter Inc., CA, USA).
- Thyroid hormones, including thyrotropin, thyroxine, and triiodothyronine (measured using a UniCel DxI 800 access immunoassay system, Beckman Coulter Inc., CA, USA).

- Blood count and biochemistry markers, including iron, ferritin, and folic acid (measured using a AU5800 automated analyzer, Beckman Coulter Inc., CA, USA).

Blood samples will be also stored at -80°C for future analysis. The systolic and diastolic blood pressure will be measured with an automatic monitor (M3-Comfort, Omron Healthcare Europe B.V. Hoofddorp, The Netherlands, in both Granada and Pamplona) following the 2021 European Society of Hypertension practice guidelines [22].

Glycemic control

Participants will wear a continuous glucose monitoring (CGM) device (FreeStyle LibrePro, Abbott, in Granada and FreeStyle 2, Abbott, in Pamplona) during two weeks before the intervention (lead-in period) and during the last two weeks of the intervention. From the CGM data several variables of glycemic control will be calculated according to the last international consensus statement [23]. Data from the CGM will be processed using an R code located in https://github.com/DIVA-soft/CGManalyzer. Concurrently, sleep physical activity levels will be objectively measured using accelerometers.

Sleep and physical activity assessment

Sleep, chronotype, and physical activity will be subjectively assessed using validated questionnaires including the Pittsburgh Sleep Quality Index (PSQI), Munich Chronotype Questionnaire (MCTQ), Horne y Östberg (MEQ-SA), and the International physical activity questionnaire (IPAQ). Sleep and physical activity levels will be also objectively assessed using accelerometers. Specifically, participants will wear a triaxial accelerometer (ActiGraph GT3X+, Pensacola, FL, USA, in both Granada and Pamplona) on the non-dominant wrist during two weeks before the intervention (lead-in period) and during the last two weeks of the intervention. The data will be processed in the GGIR R package (version 2.10-3). Accelerometer and CGM data will be time-matched using the experimental tool that can be accessed in https://github.com/DIVA-soft/GGIRmatcher.

Gut microbiota

Faecal samples will be collected at baseline and during the last two weeks of the intervention to extract genomic DNA and thereafter, to perform the metagenomics analysis (16S rRNA gene amplicon sequencing – shotgun methodology will be considered pending the final budget) to obtain a complete description of gut microbioma diversity and composition. Besides, a faecal metabolomic fingerprint analysis will be carried out in order to establish the metabolic profile in the different groups of patients by liquid chromatography method coupled to mass spectrometry (LC-MS).

Psychosocial assessment

Participants will complete validated questionnaires regarding several psychosocial dimensions: Beck Depression Inventory Fast Screen (BDI-FS), Perceived Stress Scale (PSS), and State-Trait Anxiety Inventory (STAI).

Eating behaviour assessment

Eating behaviour will be assessed using validated questionnaires: Food Craving Inventory (FCI) and the Adult Eating Behavior Questionnaire (AEBQ).

Dietary habits assessment

Participants will complete a validated questionnaire to assess their adherence to the Mediterranean dietary pattern [24]. Moreover, three non-consecutive 24h dietary recalls (two of working days and one of non-working day) will be recorded in a face-to-face or telephone interview by qualified and trained research dietitians.

Quality of life

Quality of life will be evaluated using the EuroQol 5 dimensions 5 levels (EQ-5D-5L), Rand Short Form 36 (SF-36), an adverse events questionnaire and menstrual questionnaire.

Table 2. Overview of the study's outcomes.

Outcomes	Baseline	12-week post intervention
Primary outcome		
Visceral adipose tissue (i.e., MRI)	✔	✔
Secondary outcomes		
Body weight and anthropometry	✔	✔
Body composition (i.e., DXA)	✔	✔
Ectopic fat depots (i.e., MRI)	✔	✔
Cardiometabolic risk factors	✔	✔
Interstitial glycemic traits (i.e., CGM)	✔	✔ *
Gut microbiota	✔	✔ *
Sleep and physical activity (i.e., ACC)	✔	✔ *
Psychosocial assessment	✔	✔
Eating behaviour assessment	✔	✔
Dietary habits	✔	✔
Quality of life	✔	✔

* Assessment will be conducted during the last two weeks of the intervention. Abbreviations: MRI, magnetic resonance imaging; DXA, dual-energy X-ray absorptiometry; CGM, continuous-glucose monitoring; ACC, accelerometry.

3.8 Time-restricted eating intervention

Participants will be randomly assigned to one of the following four groups:

- UC: Participants in the UC group will continue with their dietary eating time schedule and will receive, as well as the participants in the TRE groups, an educational program for weight management and cardiovascular health promotion based on Mediterranean dietary pattern [25] and physical activity recommendations from the World Health Organization [26].
- Early TRE: Participants will select an early 8h eating window (i.e., starting not later than 10:00) before the intervention, and will maintain the same 8h eating window during the 12-week intervention.
- Late TRE: Participants will select a late 8h eating window (i.e., starting not before 13:00) before the intervention, and will maintain the same 8h eating window during the 12-week intervention.
- Self-selected TRE: Participants will select their preferred 8h eating window before the intervention and will maintain the same 8h eating window during the 12-week intervention.

No calorie-containing food or beverage intake will be allowed outside the eating window for the three TRE groups. Only water, coffee, and tea without sugar or artificial sweeteners will be allowed outside the eating window for the TRE groups. Participants in the TRE groups will be instructed to perform the TRE intervention every day of the week (i.e., seven days).

3.9 Usual-care intervention

Intervention meetings for all groups will take place every two weeks by experienced nutritionists. It will be voluntary, but attendance to the meetings will be recorded. There will be 6 topics to be addressed in each intervention meeting: (i) healthy lifestyle based on Mediterranean dietary pattern and physical activity recommendations; (ii) organization and planning of food intake; (iii) control of hunger and satiety; (iv) nutritional labelling; (v) nutritional myths; and (vi) healthy snacks. After each meeting, experienced research nutritionists will answer the doubts/questions of the participants.

3.10 Assessment of adherence and adverse events

Every day during the 12-week intervention, all participants will record their time of sleep, eating, and any potential adverse event using a mobile phone app (EXTREME: com.nnbi.app_extreme, NNBi2020 S.L., Navarra, Spain). Participants will record the exact time of initiating their first meal and concluding their last meal over the 12-week intervention period. If the records indicate that participants eat within their allocated window of 8h (±30 minutes) that day will be labelled as "adherent".

The Study Coordinators will record the frequency of adverse events and report them to the Principal Investigators weekly. If a serious adverse event or unanticipated problem is reported, the Study Coordinators will immediately notify the Principal Investigators and Medical Monitor, who will determine if it is necessary to inform the ethics committee and determine the appropriate course of action to address the event.

3.11 Participants retention

Every effort will be made by the Principal Investigators and study team to ensure participants complete each study visit and the study overall. We will use the following strategies to help to maximize retention and minimize loss to follow-up:

- Following a proactive plan for retention, building participant relations and participant satisfaction. Including asking participants how they are feeling during the intervention meetings.
- Giving participants and their families the opportunity to ask questions and express concerns pertaining to their condition throughout the study.
- Enhancing participant's understanding of the study's objectives and the protocol by reminding the participant of the study aim during study visits or having question and answer sessions after each visit, if needed.
- Assessing each participant's drop-out potential and intervening as needed to keep participants interested in continuing to participate.

3.12 Feasibility

We will assess the feasibility of the intervention through various measures, including:

- Adherence monitoring: Participants will use a custom mobile phone app (EXTREME: com.nnbi.app_extreme, NNBi2020 S.L., Navarra, Spain). developed specifically for this study to track and record their daily eating and sleep times. This data will provide insights into their adherence to the prescribed eating window.
- Acceptability: During the biweekly educational sessions, we will gather feedback from participants who are undergoing the TRE intervention. This feedback will help us to understand their experiences and perceptions of the intervention, providing valuable insights into the acceptance and integrity of the intervention in their everyday lives. Participants will also have the opportunity to report any adverse events related to the intervention.
- Adverse effects and health-related questionnaires: Participants will have the ability to report any adverse effects they experience on a daily basis through the mobile phone app (EXTREME: com.nnbi.app_extreme, NNBi2020 S.L., Navarra, Spain). Additionally, they will complete validated questionnaires that assess gastrointestinal and autonomic symptoms, well-being, eating behaviour, sleep quality, stress levels, mood, anxiety, and depression. These questionnaires will provide valuable information on any potential adverse effects and overall health-related outcomes.

3.13 Data management

The majority of data will be recorded directly into REDCap, which is a secure web-based platform for building and managing online research-related databases and surveys. Any data not recorded in REDCap will be stored securely on university computers under strict access control to ensure confidentiality and data integrity. To ensure data quality and integrity, we will perform regular data quality control checks that may identify potential data anomalies, such as missing data or forms, out-of-range or erroneous data, inconsistent and illogical dates over time, data inconsistency across forms and visits, and incomplete fields on completed forms without a reason for missing data provided. Any identified issues will be reviewed and resolved by the research team promptly.

4. STATISCAL ANALYSIS PLAN

4.1 Randomization and blinding

The method used for randomization will be the stratified permuted block randomization. A total of ~208 patients will be randomized using both stratification and permuted blocks with random block sizes. Randomization will be stratified at each site (Granada and Pamplona) based on sex (men-women); with a total of two strata for each site. For this randomization scheme, a randomization list will be generated prior to the start of the trial; one randomization list being generated for each site and strata. A sequence of block sizes will be randomly generated where allowable block sizes will be 4 and 8. Within each block, each quarter of assignments will be randomly selected to be to one of the four possible groups (UC, early TRE, late TRE or self-selected TRE) using a parallel design (1:1:1:1 allocation ratio). As each participant is randomized into the trial, the participant will receive the next sequential assignment on the randomization list specific to his/her site and strata. The use of a random block size ensures that the next randomization assignment cannot be guessed. Because this will be a multicenter trial with two sites (Granada and Pamplona), randomization within each site will ensure that a site discontinuing participation in the trial or enrolling poorly would not affect the overall balance of the treatment groups. Stratifying by sex ensures that intervention groups are balanced on this important characteristic.

Personnel in charge of the evaluations of the primary outcome (VAT) and other ectopic MRI derived fat depots, fasting blood samples and statistical analysis will be blinded to the group assignment, whereas personnel in charge of the other measures as well as the intervention will be not blinded to the group assignment (open label).

4.2 Sample size

A recent systematic review and meta-analysis indicate that a VAT reduction of 6.1% is considered a clinically meaningful change even in the absence of weight loss [27]; previous exercise and nutrition intervention trials also showing changes in VAT ranging from 10 to 28% [28, 29]. The sample size calculations for VAT assume the study will be able to detect a mean difference of 10% in each intervention group from baseline to intervention endpoint, relative to the UC group. Therefore, assuming a standard deviation of 7% in VAT [28], the enrolment of 21 participants per arm will provide a statistical power of 90% at an alpha level of 0.008 (controlling for multiple group comparisons) to detect a minimum effect size of 10% in VAT. Considering subgroup analyses by sex and a maximum dropout rate of ~20%, we will recruit ~52 participants for each trial group; the total sample size being of ~208 participants (~104 in each study site).

To ensure a balanced sex distribution and maintain sufficient sample numbers despite projected dropouts, several strategies will be implemented:

- Recruitment process: We will employ a specific recruitment process that aims to enroll an equal number of men and women. For every woman recruited, we will request them to invite one man to participate in the study. This approach has proven successful in our previous intervention studies and has helped maintaining a balanced sex distribution [30-32].
- Sample size calculations: Our sample size calculations have taken into account subgroup analyses by sex. We have conservatively estimated a maximum dropout rate of approximately 20%. By considering this dropout rate, we have ensured that our study is adequately powered to detect the specified effect size even if there are differential dropout rates between men and women. For example, if men have a dropout rate of 5% and women have a dropout rate of

15%, our study will still have sufficient power to analyze the data separately in men and women.

- Expected dropout rate: While we have conservatively estimated the maximum dropout rate, we anticipate that the actual dropout rate will be relatively lower and similar between both sexes. This expectation is based on our previous studies and the measures we have in place to promote participant engagement and adherence [30-32].

4.3 Statistical analysis

Intervention effects on primary and secondary outcomes at 3 months after the intervention will be assessed based on repeated-measures linear mixed-effects multilevel models, which will include random cluster (site) effects [33]. Individual measures of change will therefore be modelled as the function of randomly assigned group, site, assessment time, and their interaction terms. Model-based estimations will be performed with an intention-to-treat approach (primary analyses) using the restricted maximum-likelihood method; the model assuming that missing values are missing-at-random. Analyses and estimations will also be performed with a per-protocol approach and an attrition propensity will be calculated using a logistic model predicting attrition with baseline values of site, allocation group, age, sex and body mass index. All the analysis will be conducted in men and women separately.

It should also be noted that the intervention effect assessments will not only be based on statistical and practical significance (as usually done), but also on a practical benefit approach emphasizing and reporting unadjusted values that are intuitive to human judgment and readily replicable considering the design and methodology of this project.

5. ETHICAL ISSUES

5.1 Institutional Ethics Committee Approval

This study conforms to the last revised Ethical Principles for Medical Research Involving Human Subjects comprised in the Declaration of Helsinki. The experimental protocol was approved by the Servicio Andaluz de Salud (Comité Ético de Investigación Provincial de Granada) and the Comité Ético de Investigación Clínica de Navarra (PI_2021/119).

5.2 Patient Consent

All participants will receive accurate information on the study assessments and intervention, and written informed consent from each participant will be obtained prior to any data collection. Signed copies of the Consent Form and a Patient Information Sheet will be given to participants. Those participants unable or not willing to give informed consent will not be eligible for enrollment in this study.

The Patient Information Sheet will also clearly state that participants have the right to withdraw from the study at any time without prejudice or explanation.

5.3 Confidentiality and Privacy

The confidentiality and privacy of all participants will be cautiously respected and ensured throughout the conduct of the study. All data will only be identified by a unique identification number/code given to each participant at the beginning of the trial; all data treatment and analyses being correspondingly blinded.

5. REFERENCES

1. WHO (2020) Obesity and overweight. https://www.who.int/news-room/fact-sheets/detail/obesity-and-overweight.

2. Bray GA, Heisel WE, Afshin A, Jensen MD, Dietz WH, Long M, et al. (2018) The Science of Obesity Management: An Endocrine Society Scientific Statement. Endocr Rev. 39(2):79-132. 10.1210/er.2017-00253

3. Finkelstein EA, Trogdon JG, Cohen JW, Dietz W (2009) Annual medical spending attributable to obesity: payer-and service-specific estimates. Health Aff (Millwood). 28(5):w822-31. 10.1377/hlthaff.28.5.w822

4. de Cabo R, Mattson MP (2019) Effects of Intermittent Fasting on Health, Aging, and Disease. N Engl J Med. 381(26):2541-51. 10.1056/NEJMra1905136

5. Dote-Montero M, Sanchez-Delgado G, Ravussin E (2022) Effects of Intermittent Fasting on Cardiometabolic Health: An Energy Metabolism Perspective. Nutrients. 14(3). 10.3390/nu14030489

6. Varady KA, Cienfuegos S, Ezpeleta M, Gabel K (2022) Clinical application of intermittent fasting for weight loss: progress and future directions. Nat Rev Endocrinol. 18(5):309-21. 10.1038/s41574-022-00638-x

7. Liu L, Chen W, Wu D, Hu F (2022) Metabolic Efficacy of Time-Restricted Eating in Adults: A Systematic Review and Meta-Analysis of Randomized Controlled Trials. J Clin Endocrinol Metab. 10.1210/clinem/dgac570

8. Liu D, Huang Y, Huang C, Yang S, Wei X, Zhang P, et al. (2022) Calorie Restriction with or without Time-Restricted Eating in Weight Loss. N Engl J Med. 386(16):1495-504. 10.1056/NEJMoa2114833

9. Wei X, Lin B, Huang Y, Yang S, Huang C, Shi L, et al. (2023) Effects of Time-Restricted Eating on Nonalcoholic Fatty Liver Disease: The TREATY-FLD Randomized Clinical Trial. JAMA Netw Open. 6(3):e233513. 10.1001/jamanetworkopen.2023.3513

10. Neeland IJ, Ross R, Després JP, Matsuzawa Y, Yamashita S, Shai I, et al. (2019) Visceral and ectopic fat, atherosclerosis, and cardiometabolic disease: a position statement. Lancet Diabetes Endocrinol. 7(9):715-25. 10.1016/s2213-8587(19)30084-1

11. Cooper AJ, Gupta SR, Moustafa AF, Chao AM (2021) Sex/Gender Differences in Obesity Prevalence, Comorbidities, and Treatment. Curr Obes Rep. 10(4):458-66. 10.1007/s13679-021-00453-x

12. Bhargava A, Arnold AP, Bangasser DA, Denton KM, Gupta A, Hilliard Krause LM, et al. (2021) Considering Sex as a Biological Variable in Basic and Clinical Studies: An Endocrine Society Scientific Statement. Endocr Rev. 42(3):219-58. 10.1210/endrev/bnaa034

13. Poggiogalle E, Jamshed H, Peterson CM (2018) Circadian regulation of glucose, lipid, and energy metabolism in humans. Metabolism. 84:11-27. 10.1016/j.metabol.2017.11.017

14. Sutton EF, Beyl R, Early KS, Cefalu WT, Ravussin E, Peterson CM (2018) Early Time-Restricted Feeding Improves Insulin Sensitivity, Blood Pressure, and Oxidative Stress Even without Weight Loss in Men with Prediabetes. Cell Metab. 27(6):1212-21.e3. 10.1016/j.cmet.2018.04.010

15. Liu J, Yi P, Liu F (2023) The Effect of Early Time-Restricted Eating vs. Later Time-Restricted Eating on Weight Loss and Metabolic Health: A Network Meta-Analysis of Randomized Controlled Trials. J Clin Endocrinol Metab. 10.1210/clinem/dgad036

16. Queiroz JDN, Macedo RCO, Dos Santos GC, Munhoz SV, Machado CLF, de Menezes RL, et al. (2022) Cardiometabolic effects of early v. delayed time-restricted eating plus energetic restriction in adults with overweight and obesity: an exploratory randomised clinical trial. Br J Nutr.1-13. 10.1017/s0007114522001581

17. Zhang LM, Liu Z, Wang JQ, Li RQ, Ren JY, Gao X, et al. (2022) Randomized controlled trial for time-restricted eating in overweight and obese young adults. iScience. 25(9):104870. 10.1016/j.isci.2022.104870

18. Xie Z, Sun Y, Ye Y, Hu D, Zhang H, He Z, et al. (2022) Randomized controlled trial for time-restricted eating in healthy volunteers without obesity. Nat Commun. 13(1):1003. 10.1038/s41467-022-28662-5

19. He M, Wang J, Liang Q, Li M, Guo H, Wang Y, et al. (2022) Time-restricted eating with or without low-carbohydrate diet reduces visceral fat and improves

metabolic syndrome: A randomized trial. Cell Rep Med. 3(10):100777. 10.1016/j.xcrm.2022.100777

20. Silva VSd, Vieira MFS (2020) International Society for the Advancement of Kinanthropometry (ISAK) Global: international accreditation scheme of the competent anthropometrist. Revista Brasileira de Cineantropometria & Desempenho Humano. 22.

21. Friedewald WT, Levy RI, Fredrickson DS (1972) Estimation of the concentration of low-density lipoprotein cholesterol in plasma, without use of the preparative ultracentrifuge. Clin Chem. 18(6):499-502.

22. Stergiou GS, Palatini P, Parati G, O'Brien E, Januszewicz A, Lurbe E, et al. (2021) 2021 European Society of Hypertension practice guidelines for office and out-of-office blood pressure measurement. J Hypertens. 39(7):1293-302. 10.1097/hjh.0000000000002843

23. Battelino T, Alexander CM, Amiel SA, Arreaza-Rubin G, Beck RW, Bergenstal RM, et al. (2023) Continuous glucose monitoring and metrics for clinical trials: an international consensus statement. Lancet Diabetes Endocrinol. 11(1):42-57. 10.1016/s2213-8587(22)00319-9

24. Martínez-González MA, García-Arellano A, Toledo E, Salas-Salvadó J, Buil-Cosiales P, Corella D, et al. (2012) A 14-item Mediterranean diet assessment tool and obesity indexes among high-risk subjects: the PREDIMED trial. PLoS One. 7(8):e43134. 10.1371/journal.pone.0043134

25. Estruch R, Ros E, Salas-Salvadó J, Covas MI, Corella D, Arós F, et al. (2018) Primary Prevention of Cardiovascular Disease with a Mediterranean Diet Supplemented with Extra-Virgin Olive Oil or Nuts. N Engl J Med. 378(25):e34. 10.1056/NEJMoa1800389

26. Bull FC, Al-Ansari SS, Biddle S, Borodulin K, Buman MP, Cardon G, et al. (2020) World Health Organization 2020 guidelines on physical activity and sedentary behaviour. Br J Sports Med. 54(24):1451-62. 10.1136/bjsports-2020-102955

27. Verheggen RJ, Maessen MF, Green DJ, Hermus AR, Hopman MT, Thijssen DH (2016) A systematic review and meta-analysis on the effects of exercise training versus hypocaloric diet: distinct effects on body weight and visceral adipose tissue. Obes Rev. 17(8):664-90. 10.1111/obr.12406

28. Ross R, Dagnone D, Jones PJ, Smith H, Paddags A, Hudson R, et al. (2000) Reduction in obesity and related comorbid conditions after diet-induced weight loss or exercise-induced weight loss in men. A randomized, controlled trial. Ann Intern Med. 133(2):92-103. 10.7326/0003-4819-133-2-200007180-00008

29. Ross R, Janssen I, Dawson J, Kungl AM, Kuk JL, Wong SL, et al. (2004) Exercise-induced reduction in obesity and insulin resistance in women: a randomized controlled trial. Obes Res. 12(5):789-98. 10.1038/oby.2004.95

30. Amaro-Gahete FJ, De-la OA, Jurado-Fasoli L, Dote-Montero M, Gutiérrez Á, Ruiz JR, et al. (2019) Changes in Physical Fitness After 12 Weeks of Structured Concurrent Exercise Training, High Intensity Interval Training, or Whole-Body Electromyostimulation Training in Sedentary Middle-Aged Adults: A Randomized Controlled Trial. Front Physiol. 10:451. 10.3389/fphys.2019.00451

31. Labayen I, Medrano M, Arenaza L, Maíz E, Osés M, Martínez-Vizcaíno V, et al. (2020) Effects of Exercise in Addition to a Family-Based Lifestyle Intervention Program on Hepatic Fat in Children With Overweight. Diabetes Care. 43(2):306-13. 10.2337/dc19-0351

32. Martinez-Tellez B, Sanchez-Delgado G, Acosta FM, Alcantara JMA, Amaro-Gahete FJ, Martinez-Avila WD, et al. (2022) No evidence of brown adipose tissue activation after 24 weeks of supervised exercise training in young sedentary adults in the ACTIBATE randomized controlled trial. Nat Commun. 13(1):5259. 10.1038/s41467-022-32502-x

33. Bates D, Mächler M, Bolker B, Walker S (2015) Fitting Linear Mixed-Effects Models Using lme4. Journal of Statistical Software. 67(1):1 - 48. 10.18637/jss.v067.i01

34. Mattson MP, Allison DB, Fontana L, Harvie M, Longo VD, Malaisse WJ, et al. (2014) Meal frequency and timing in health and disease. Proc Natl Acad Sci U S A. 111(47):16647-53. 10.1073/pnas.1413965111

35. Lopez-Minguez J, Gómez-Abellán P, Garaulet M (2019) Timing of Breakfast, Lunch, and Dinner. Effects on Obesity and Metabolic Risk. Nutrients. 11(11). 10.3390/nu11112624

36. Mantovani A, Csermely A, Petracca G, Beatrice G, Corey KE, Simon TG, et al. (2021) Non-alcoholic fatty liver disease and risk of fatal and non-fatal cardiovascular

events: an updated systematic review and meta-analysis. Lancet Gastroenterol Hepatol. 6(11):903-13. 10.1016/s2468-1253(21)00308-3

37. Mantovani A, Petracca G, Beatrice G, Tilg H, Byrne CD, Targher G (2021) Non-alcoholic fatty liver disease and risk of incident diabetes mellitus: an updated meta-analysis of 501 022 adult individuals. Gut. 70(5):962-9. 10.1136/gutjnl-2020-322572

38. Mantovani A, Petracca G, Csermely A, Beatrice G, Bonapace S, Rossi A, et al. (2022) Non-alcoholic fatty liver disease and risk of new-onset heart failure: an updated meta-analysis of about 11 million individuals. Gut. 10.1136/gutjnl-2022-327672

39. Correa-de-Araujo R, Addison O, Miljkovic I, Goodpaster BH, Bergman BC, Clark RV, et al. (2020) Myosteatosis in the Context of Skeletal Muscle Function Deficit: An Interdisciplinary Workshop at the National Institute on Aging. Front Physiol. 11:963. 10.3389/fphys.2020.00963

40. Cadenas-Sanchez C, Idoate F, Villanueva A, Cabeza R, Labayen I (2021) Intermuscular abdominal fat fraction and metabolic dysfunction-associated fatty liver disease: Does the link already exist in childhood? J Hepatol. 75(6):1511-3. 10.1016/j.jhep.2021.05.011

41. Nachit M, Kwanten WJ, Thissen JP, Op De Beeck B, Van Gaal L, Vonghia L, et al. (2021) Muscle fat content is strongly associated with NASH: A longitudinal study in patients with morbid obesity. J Hepatol. 75(2):292-301. 10.1016/j.jhep.2021.02.037

6. SUMMARY OF CHANGES TO STUDY PROTOCOL

Date	Amendment
22/10/2021	Institutional Ethics Committee approvals: Comité Ético de Investigación Clínica de Navarra (PI_2021/119). Section 3.1
26/01/2022	EXTREME: com.nnbi.app_extreme, NNBi2020 S L., Navarra, Spain
2/02/2022	Institutional Ethics Committee approvals: Servicio Andaluz de Salud (Comité Ético de Investigación Provincial de Granada). Section 3.1
05/04/2022	First posted at ClinicalTrials.gov
22/06/2023	Development of an R script for analyzing continuous glucose monitoring R available at https://github.com/DIVA-soft/CGManalyzer. Section 3.7
22/06/2023	Development of an R script for analyzing data from accelerometers and continuous glucose monitors that have been time-aligned using the experimental tool available at https://github.com/DIVA-soft/GGIRmatcher. Section 3.7
22/06/2023	Data analysis of accelerometer-derived outcomes using an R script with the version 2.10-3. Section 3.7
