# Supplementary material for: Time-Restricted Eating and Sleep, Mood, and Quality of Life in Adults With Overweight or Obesity: A Secondary Analysis of a Randomized Clinical Trial
Source: JAMA Netw Open. 2025 Jun 25;8(6):e2517268. doi: 10.1001/jamanetworkopen.2025.17268 (PMC12199060; doi:10.1001/jamanetworkopen.2025.17268)
Supplement: Supplement 2. — eMethods 1. Study Assessments and End Points eMethods 2. Sensitivity Analyses eTable 1. Sleep, Mood, and Quality-of-Life End Points at Baseline and After the 12-Week Intervention in Each Intervention Group eTable 2. Changes in Sleep End Points in the Time-Restricted Eating Groups Compared With the Usual Care Group After the 12-Week Intervention Considering the Daylight Time in the Analysis eTable 3. Changes in Sleep End Points in the Time-Restricted Eating Groups Compared With Each Other After the 12-Week Intervention Considering the Daylight Time in the Analysis eTable 4. Sleep End Points at Baseline and After the 12-Week Intervention in Each Intervention Group Considering the Daylight Time in the Analysis eTable 5. Changes in Sleep End Points in the Time-Restricted Eating Groups Compared With the Usual Care Group Following the 12-Week Intervention, After Excluding Participants Who Reported Taking Sleep Medication at Baseline eTable 6. Changes in Sleep End Points in the Time-Restricted Eating Groups Compared With Each Other Following the 12-Week Intervention, After Excluding Participants Who Reported Taking Sleep Medication at Baseline eTable 7. Sleep End Points at Baseline and Following the 12-Week Intervention in Each Intervention Group, After Excluding Participants Who Reported Taking Sleep Medication at Baseline eFigure 1. Study Flowchart eFigure 2. Changes in General Health Across All Groups After the 12-Week Intervention eTable 8. Baseline Characteristics of All Randomized Participants by Sex and Intervention Group eTable 9. Changes in Sleep, Mood, and Quality-of-Life End Points in the Time-Restricted Eating Groups Compared With the Usual Care Group After the 12-Week Intervention, Divided by Sex eTable 10. Changes in Sleep, Mood, and Quality-of-Life End Points in the Time-Restricted Eating Groups Compared With Each Other After the 12-Week Intervention, Divided by Sex eFigure 3. Changes in Sleep Outcomes Across All Groups After the 12-Week Intervention, Divide [file jamanetwopen-e2517268-s002.pdf]

Supplementary Online Content

Clavero-Jimeno A, Dote-Montero M, Migueles JH, et al. Time-restricted eating and sleep, mood, and quality of life in adults with overweight or obesity: a secondary analysis of a randomized clinical trial. *JAMA Netw Open*. 2025;8(6):e2517268. doi:10.1001/jamanetworkopen.2025.17268

eMethods 1. Study Assessments and End Points

eMethods 2. Sensitivity Analyses

eTable 1. Sleep, Mood, and Quality-of-Life End Points at Baseline and After the 12-Week Intervention in Each Intervention Group

eTable 2. Changes in Sleep End Points in the Time-Restricted Eating Groups Compared With the Usual Care Group After the 12-Week Intervention Considering the Daylight Time in the Analysis

eTable 3. Changes in Sleep End Points in the Time-Restricted Eating Groups Compared With Each Other After the 12-Week Intervention Considering the Daylight Time in the Analysis

eTable 4. Sleep End Points at Baseline and After the 12-Week Intervention in Each Intervention Group Considering the Daylight Time in the Analysis

eTable 5. Changes in Sleep End Points in the Time-Restricted Eating Groups Compared With the Usual Care Group Following the 12-Week Intervention, After Excluding Participants Who Reported Taking Sleep Medication at Baseline

eTable 6. Changes in Sleep End Points in the Time-Restricted Eating Groups Compared With Each Other Following the 12-Week Intervention, After Excluding Participants Who Reported Taking Sleep Medication at Baseline

eTable 7. Sleep End Points at Baseline and Following the 12-Week Intervention in Each Intervention Group, After Excluding Participants Who Reported Taking Sleep Medication at Baseline

eFigure 1. Study Flow Chart

eFigure 2. Changes in General Health Across All Groups After the 12-Week Intervention

eTable 8. Baseline Characteristics of All Randomized Participants by Sex and Intervention Group

eTable 9. Changes in Sleep, Mood, and Quality-of-Life End Points in the Time-Restricted Eating Groups Compared With the Usual Care Group After the 12-Week Intervention, Divided by Sex

eTable 10. Changes in Sleep, Mood, and Quality-of-Life End Points in the Time-Restricted Eating Groups Compared With Each Other After the 12-Week Intervention, Divided by Sex

eFigure 3. Changes in Sleep Outcomes Across All Groups After the 12-Week Intervention, Divided by Sex

eFigure 4. Changes in Mood Outcomes Across All Groups After the 12-Week Intervention, Divided by Sex

© 2025 Clavero-Jimeno A et al. *JAMA Network Open*.

eFigure 5. Changes in General Health Across All Groups After the 12-Week Intervention, Divided by Sex

eTable 11. Sleep, Mood, and Quality-of-Life End Points at Baseline and After the 12-Week Intervention in Each Intervention Group in Men

eTable 12. Sleep, Mood, and Quality-of-Life End Points at Baseline and After the 12-Week Intervention in Each Intervention Group in Women

eTable 13. Changes in Sleep End Points in the Time-Restricted Eating Groups Compared With the Usual Care Group After the 12-Week Intervention Considering the Daylight Time in the Analysis, Divided by Sex

eTable 14. Changes in Sleep End Points in the Time-Restricted Eating Groups Compared With Each Other After the 12-Week Intervention Considering the Daylight Time in the Analysis, Divided by Sex

eTable 15. Sleep End Points at Baseline and After the 12-Week Intervention Considering the Daylight Time in the Analysis in Men

eTable 16. Sleep End Points at Baseline and After the 12-Week Intervention Considering the Daylight Time in the Analysis in Women

eReferences

This supplementary material has been provided by the authors to give readers additional information about their work.

© 2025 Clavero-Jimeno A et al. *JAMA Network Open*.

eMethods 1. Study Assessments and End Points

Assessment of Antropometry and Body Composition

Body weight and height were measured following the International Society for the Advancement of Kinanthropometry procedures,[1] using a stadiometer and a scale (Seca model 799, Electronic Column Scale, Hamburg, Germany). Body mass index was calculated as weight in kilograms divided by height in meters squared. Fat-free mass and fat mass percentage were assessed using a dual-energy X-ray absorptiometry scan, i.e., the Hologic QDR-4500 W in Granada and the Hologic Horizon-WI (Hologic Inc., Bedford, Massachusetts, USA) in Pamplona. All assessments were conducted in the morning after a 10 to 12-hour fasting period.

Assessment of Sleep, Mood, and Quality of Life

Sleep habits were monitored using a triaxial accelerometer (ActiGraph GT3X+, ActiGraph LLC, Pensacola, Florida, USA, in Granada and Pamplona). The accelerometers collected raw accelerations at a frequency of 90 Hz for 14 consecutive days at each time point. The GGIR R package (version 3.1-6)[2] was used to process the raw accelerations, including auto-calibration, detection and imputation of non-wear and invalid time recorded, and detection of the sleep period time and sleep-related outcomes. Sleep and awake periods were identified using an automated algorithm based on the variability of the arm posture and guided by the participants' reported sleep times,[3,4] specifically the HDCZA algorithm in the GGIR R package.[2]

Perceived sleep quality was assessed using the Pittsburgh Sleep Quality Index (PSQI) questionnaire at baseline and after the 12-week intervention. The PSQI is a valid and reliable measure of sleep quality over a 1-month period.[5] The PSQI total score was calculated by summing the scores of the 7 subscales (subjective sleep quality, sleep latency, sleep time, habitual sleep efficiency, sleep disturbances, use of sleeping medications, and daytime dysfunction). The score range for the PSQI subscales is 0 to 3, and the total score range is from 0 to 21 points, with higher scores indicating worse sleep quality.

Mood outcomes were assessed at baseline and after the 12-week intervention using self-reported questionnaires. Depression was assessed using the Beck Depression Inventory Fast Screen (BDI-FS).[6] The BDI-FS is a reliable depression screening tool for medical patients, focusing exclusively on cognitive-affective symptoms of depression. The BDI-FS consists of a 7-item Likert-based scale including sadness, pessimism, past failure, loss of pleasure, self-dislike, self-criticalness, and suicidal thoughts. The score range for each BDI-FS item is 0 to 3, and the total score range is from 0 to 21 points, with higher scores reflecting more depressive symptomatology.

Anxiety was assessed using the State-Trait Anxiety Inventory (STAI).[7] The STAI is a 40-item Likert that evaluates state anxiety (items 1-20), which assesses the level of anxiety experienced at the time of assessment, and the trait anxiety (items 21-40), which assesses the general, longstanding tendency to experience anxiety. Each item is scored on a 4-point Likert scale ranging from “not at all” to “very much so” (0–3). The total score range for the STAI goes from 0 to 60 points for each subscale, with higher scores reflecting a greater degree of anxiety.

Perceived stress was assessed using the Perceived Stress Scale (PSS).[8] The PSS is designed to assess how individuals perceive their lives as stressful. The items specifically measure the degree to which one's life is viewed as unpredictable, uncontrollable, and overwhelming. The questions were examined on a 5-point Likert-type scale, ranging “never” to “very often” (0–4). Scores were obtained by reverse scoring the

© 2025 Clavero-Jimeno A et al. *JAMA Network Open*.

positively stated items (4, 5, 6, 7, 9, 10, and 13). The total score range goes from 0 to 56 by summing the scores of all 14 items, with a higher score indicating greater degree of perceived stress.

Quality of life was assessed using the self-reported Rand 36-Item Short Form (SF-36) questionnaire.[9] The SF-36 is composed by eight subscales: four that measure mental aspects of quality of life (role limitations due to emotional problems, vitality, social functioning, mental health) and four that measure physical aspects of quality of life (physical functioning, role limitations due to physical problems, bodily pain, general health). The total score range for the SF-36 goes from 0 to 100 points, with higher scores indicating better quality of life.

© 2025 Clavero-Jimeno A et al. *JAMA Network Open*.

eMethods 2. Sensitivity Analyses

As daylight hours may affect sleep over the year,[10] and given that the assessments were conducted in different seasons, it is important to consider this factor in the analyses. We derived a standardized score with a minimum value of -1 at the Winter Solstice, indicating the shortest daylight time of the year, and a maximum value of +1 at the Summer Solstice, indicating the longest daylight time of the year. Therefore, we repeated the analyses considering this factor to ensure the consistency of the results. Then, we also conducted sensitivity analyses adjusting for baseline sleep medication use, defined as participants reporting taking sleep medication at least once a week. We repeated the analysis excluding those participants that reported sleep medication use to examine its influence on the results. In addition, we conducted sensitivity and post hoc analyses by performing the same repeated-measures linear mixed-effects multilevel models, but stratifying the analysis by sex (i.e., separately for men and women). All statistical analyses and figures were performed in R version 4.4.2 (https://cran.r-project.org/, The R Project for Statistical Computing, Vienna, Austria); linear mixed-effects models were performed using the lme4 package for R, version 1.1-36.

© 2025 Clavero-Jimeno A et al. *JAMA Network Open*.

eTable 1. Sleep, Mood, and Quality-of-Life End Points at Baseline and After the 12-Week Intervention in Each Intervention Group

End point	UC (n = 49)	Early TRE (n = 49)	Late TRE (n = 52)	Self-selected TRE (n = 47)
	Mean (95% CI)[a]	Mean (95% CI)[a]	Mean (95% CI)[a]	Mean (95% CI)[a]
**Sleep**				
Onset				
Preintervention, h:min	00:24 (00:06, 00:36)	00:12 (23:54, 00:30)	00:12 (23:54, 00:24)	00:00 (23:48, 00:12)
Postintervention, h:min	00:24 (00:06, 00:36)	00:06 (23:54, 00:18)	00:12 (23:54, 00:24)	00:00 (23:48, 00:12)
Change, h	0.0 (-0.2, 0.2)	-0.1 (-0.3, 0.1)	0.0 (-0.2, 0.2)	0.0 (-0.1, 0.2)
Offset				
Preintervention, h:min	07:30 (07:18, 07:42)	07:30 (07:12, 07:42)	07:30 (07:12, 07:42)	07:24 (07:12, 07:36)
Postintervention, h:min	07:24 (07:18, 07:36)	07:24 (07:06, 07:36)	07:12 (06:54, 07:24)	07:12 (07:00, 07:30)
Change, h	-0.1 (-0.2, 0.1)	-0.1 (-0.3, 0.1)	-0.3 (-0.5, 0.0) *	-0.2 (-0.4, 0.0)
Period, h[b]				
Preintervention	7.1 (6.9, 7.4)	7.2 (7.0, 7.5)	7.3 (7.0, 7.5)	7.4 (7.2, 7.7)
Postintervention	7.0 (6.8, 7.4)	7.3 (7.0, 7.5)	7.0 (6.8, 7.3)	7.2 (7.0, 7.5)
Change	-0.1 (-0.3, 0.1)	0.0 (-0.2, 0.3)	-0.2 (-0.5, 0.0) *	-0.2 (-0.5, 0.0)
Total time, h[c]				
Preintervention	6.2 (5.9, 6.4)	6.3 (6.1, 6.5)	6.4 (6.1, 6.6)	6.4 (6.2, 6.7)
Postintervention	6.0 (5.7, 6.2)	6.3 (6.1, 6.5)	6.1 (5.8, 6.3)	6.2 (5.9, 6.4)
Change	-0.2 (-0.4, 0.0) *	0.0 (-0.2, 0.2)	-0.3 (-0.5, -0.1) *	-0.3 (-0.5, -0.1) *
Efficiency, %				
Preintervention	86.8 (85.2, 88.4)	86.4 (84.4, 87.6)	87.5 (85.9, 89.0)	86.4 (84.7, 88.0)
Postintervention	85.9 (83.8, 86.8)	86.0 (84.4, 87.6)	86.8 (85.2, 88.5)	85.1 (83.3, 86.8)
Change	-1.6 (-2.8, -0.5) *	-0.4 (-1.5, -0.8)	-0.7 (-1.8, -0.4)	-1.3 (-2.6, 0.0) *
Awakenings, No.				
Preintervention	13.4 (12.5, 14.3)	14.4 (13.5, 14.5)	13.9 (13.0, 14.8)	14.7 (13.7, 15.7)
Postintervention	12.8 (11.8, 13.7)	14.0 (13.0, 14.9)	12.9 (12.0, 13.8)	13.8 (12.8, 14.7)
Change	-0.6 (-1.3, 0.1)	-0.5 (-1.2, 0.2)	-1.0 (-1.7, -0.3) *	-0.9 (-1.6, -0.3) *
Wake after sleep onset, h				
Preintervention	0.9 (0.8, 1.0)	1.0 (0.9, 1.1)	0.9 (0.8, 1.0)	1.0 (0.9, 1.1)
Postintervention	1.0 (0.9, 1.1)	1.0 (0.9, 1.1)	0.9 (0.8, 1.1)	1.1 (0.9, 1.2)
Change	0.1 (0.0, 0.2) *	0.1 (0.0, 0.1)	0.0 (-0.1, 0.1)	0.1 (0.0, 0.1)
Sleep quality score[d]				
Preintervention	6.4 (5.6, 7.2)	5.9 (5.1, 6.7)	5.9 (5.2, 6.7)	7.1 (6.3, 7.8)
Postintervention	5.7 (4.9, 6.5)	5.6 (4.8, 6.5)	5.4 (4.6, 6.1)	6.2 (5.3, 7.0)
Change	-0.7 (-1.4, -0.1)	-0.3 (-1.7, 0.5)	-0.4 (-0.9, -0.5)	-0.9 (-1.8, -0.2) *
**Mood scores**				
Depression[e]				
Preintervention	2.1 (1.5, 2.7)	2.1 (1.4, 2.7)	2.2 (1.6, 2.8)	2.5 (1.9, 3.2)
Postintervention	1.2 (0.5, 1.9)	1.3 (0.6, 2.0)	1.2 (0.5, 1.8)	1.6 (0.9, 2.3)
Change	-0.9 (-1.6, -0.3) *	-0.8 (-1.4, -0.1) *	-1.1 (-1.6, -0.5) *	-0.9 (-1.6, -0.3) *
State anxiety[f]				
Preintervention	15.7 (12.9, 18.4)	17.1 (14.4, 19.8)	14.3 (11.6, 16.9)	18.2 (15.4, 21.0)
Postintervention	14.8 (11.7, 17.8)	15.0 (12.1, 17.9)	11.5 (8.8, 14.3)	16.6 (13.6, 19.7)
Change	-0.9 (-3.8, 1.9)	-2.1 (-4.9, 0.7)	-2.7 (-5.3, -0.1) *	-1.6 (-4.4, 1.3)
Trait anxiety[f]				
Preintervention	17.6 (14.9, 20.2)	19.2 (16.5, 21.8)	17.5 (15.0, 20.1)	20.9 (18.2, 23.6)
Postintervention	15.6 (12.7, 18.4)	17.6 (14.8, 20.3)	14.7 (12.0, 17.3)	19.4 (16.5, 22.2)
Change	-2.0 (-4.1, 0.1)	-1.6 (-3.6, 0.4)	-2.9 (-4.7, -1.0) *	-1.5 (-3.6, 0.5)

© 2025 Clavero-Jimeno A et al. *JAMA Network Open*.

eTable 1. Sleep, Mood, and Quality-of-Life End Points at Baseline and After the 12-Week Intervention in Each Intervention Group (continued)

End point	UC (n = 49)	Early TRE (n = 49)	Late TRE (n = 52)	Self-selected TRE (n = 47)
	**Mean (95% CI)[a]**	**Mean (95% CI)[a]**	**Mean (95% CI)[a]**	**Mean (95% CI)[a]**
Stress[g]				
Preintervention	24.0 (21.7, 26.3)	23.2 (20.9, 25.5)	23.5 (21.3, 25.8)	25.9 (23.6, 28.3)
Postintervention	21.8 (19.4, 24.3)	23.1 (20.6, 25.5)	21.6 (19.3, 23.9)	23.6 (21.1, 26.2)
Change	-2.2 (-4.3, -0.1) *	-0.1 (-2.1, 1.9)	-1.9 (-3.8, 0.0) *	-2.3 (-4.4, -0.2) *
**Quality-of-life scores[h]**				
Physical functioning				
Preintervention	88.4 (85.5, 91.4)	91.8 (88.8, 94.8)	87.5 (84.7, 90.4)	88.0 (85.0, 91.0)
Postintervention	91.1 (87.8, 94.3)	94.1 (90.9, 97.2)	93.2 (90.2, 96.2)	92.5 (89.3, 95.8)
Change	2.6 (-0.5, 5.8)	2.3 (-0.8, 5.4)	5.7 (2.8, 8.6) *	4.5 (1.4, 7.7) *
Role limitations				
Due to physical health				
Preintervention	84.6 (76.4, 92.8)	90.4 (82.1, 98.8)	86.8 (78.9, 94.8)	79.5 (71.1, 87.9)
Postintervention	86.5 (77.2, 95.9)	84.3 (75.3, 93.3)	92.6 (84.0, 101.2)	84.6 (75.2, 93.9)
Change	1.9 (-8.4, 12.3)	-6.1 (-16.3, 4.1)	5.8 (-3.9, 15.4)	5.0 (-5.4, 15.5)
Due to emotional problems				
Preintervention	90.4 (82.5, 98.3)	82.9 (74.9, 91.0)	94.1 (86.4, 101.7)	74.7 (66.7, 82.8)
Postintervention	85.2 (76.3, 94.2)	85.3 (76.7, 94.0)	96.4 (88.2, 104.7)	87.2 (78.2, 96.1)
Change	-5.2 (-15.1, 4.8)	2.4 (-7.4, 12.1)	2.3 (-6.9, 11.6)	12.4 (2.4, 22.4) *
Vitality				
Preintervention	54.3 (49.3, 59.4)	53.6 (48.5, 58.6)	48.8 (43.9, 53.7)	48.5 (43.4, 53.7)
Postintervention	55.9 (50.5, 61.3)	56.2 (50.9, 61.4)	56.4 (51.4, 61.5)	57.1 (51.6, 62.5)
Change	1.5 (-2.6, 5.7)	2.6 (-1.5, 6.7)	7.6 (3.8, 11.5) *	8.5 (4.4, 12.7) *
Social functioning				
Preintervention	69.6 (62.9, 76.2)	70.9 (64.2, 77.6)	72.5 (66.1, 79.0)	68.4 (61.6, 75.2)
Postintervention	70.9 (63.9, 77.8)	67.8 (60.9, 74.6)	73.1 (66.5, 79.7)	65.6 (58.6, 72.6)
Change	1.3 (-3.0, 5.6)	-3.2 (-7.4, 1.1)	0.6 (-3.4, 4.5)	-2.8 (-7.1, 1.5)
Bodily pain				
Preintervention	79.8 (74.0, 85.6)	82.2 (76.3, 88.1)	77.9 (72.3, 83.5)	69.5 (63.5, 75.4)
Postintervention	78.3 (71.8, 84.9)	78.0 (71.7, 84.3)	79.1 (73.0, 85.1)	78.5 (72.0, 85.0)
Change	-1.4 (-8.2, 5.3)	-4.1 (-10.8, 2.5)	1.2 (-5.1, 7.4)	9.0 (2.3, 15.8) *
General health				
Preintervention	66.3 (61.7, 70.9)	65.5 (60.8, 70.2)	61.4 (56.9, 65.9)	61.6 (56.8, 66.3)
Postintervention	67.9 (62.9, 72.9)	70.3 (65.4, 75.3)	69.9 (65.1, 74.6)	66.0 (60.9, 71.1)
Change	1.6 (-2.5, 5.8)	4.9 (0.8, 9.0) *	8.5 (4.7, 12.3) *	4.4 (0.3, 8.6) *

Abbreviations: CI, confidence interval; TRE, time-restricted eating; UC, usual care.
[a] Asterisks (*) indicate statistically significant within-group differences as determined by 2-sided t-test (*P* < .05). No adjustments were made for multiple comparisons within groups. Changes were calculated as postintervention minus preintervention values.
[b] Time from sleep onset to sleep offset.
[c] Amount of time classified as sleep within the sleep period.
[d] Assessed using the Pittsburgh Sleep Quality Index (score range, 0-21 points, with higher scores indicating worse sleep quality).
[e] Assessed using the Beck Depression Inventory Fast Screen (score range, 0-21 points, with higher scores reflecting more depressive symptoms).
[f] Assessed using the State-Trait Anxiety Inventory (total score range, 0-60 points for state anxiety and trait anxiety, with higher scores reflecting greater anxiety).
[g] Assessed using the Perceived Stress Scale (score range, 0-56 points, with higher scores indicating greater perceived stress).
[h] Assessed using the Rand 36-Item Short Form Health Survey (score range, 0-100 points, with higher scores reflecting better quality of life).

© 2025 Clavero-Jimeno A et al. *JAMA Network Open*.

eTable 2. Changes in Sleep End Points in the Time-Restricted Eating Groups Compared With the Usual Care Group after the 12-Week Intervention Considering the Daylight Time in the Analysis

End point	Early TRE vs. UC	Late TRE vs. UC	Self-selected TRE vs. UC
	Difference, mean (95% CI)[a]	Difference, mean (95% CI)[a]	Difference, mean (95% CI)[a]
**Sleep**			
Onset, h	-0.2 (-0.5, 0.2)	0.0 (-0.5, 0.4)	0.0 (-0.4, 0.4)
Offset, h	0.0 (-0.4, 0.4)	-0.2 (-0.6, 0.3)	-0.1 (-0.5, 0.4)
Period, h[b]	0.1 (-0.3, 0.6)	-0.2 (-0.6, 0.3)	-0.1 (-0.5, 0.4)
Total time, h[c]	0.2 (-0.2, 0.6)	-0.1 (-0.4, 0.3)	0.0 (-0.4, 0.4)
Efficiency, %	1.3 (-0.9, 3.4)	1.0 (-1.1, 3.1)	0.3 (-1.8, 2.5)
Awakenings, No.	0.1 (-1.2, 1.4)	-0.4 (-1.7, 0.9)	-0.3 (-1.7, 1.0)
Wake after sleep onset, h	-0.1 (-0.2, 0.1)	-0.1 (-0.3, 0.1)	0.0 (-0.2, 0.1)
Sleep quality score[d]	0.5 (-1.0, 1.9)	0.6 (-0.9, 2.0)	-0.3 (-1.7, 1.2)

Abbreviations: CI, confidence interval; TRE, time-restricted eating; UC, usual care.

[a] Data are presented as estimated mean differences and 95% CI between groups and were calculated by first computing the postintervention minus the preintervention values within each group; then, differences between the groups were computed as early TRE minus UC, late TRE minus UC, and self-selected TRE minus UC. We derived a standardized score which had its minimum value at the Winter Solstice (i.e., -1), indicating the lowest daylight time in the year; and its maximum value at the Summer Solstice (i.e., +1), indicating the longest daylight time in the year. Sample size: UC, n = 49; early TRE, n = 49; late TRE, n = 52; self–selected TRE, n = 47. No statistically significant differences were detected in changes in sleep outcomes in the TRE groups compared to the UC group considering the daylight time in the analysis.

[b] Time from sleep onset to sleep offset.

[c] Amount of time classified as sleep within the sleep period.

[d] Assessed using the Pittsburgh Sleep Quality Index (score range, 0-21 points, with higher scores indicating worse sleep quality).

© 2025 Clavero-Jimeno A et al. *JAMA Network Open*.

eTable 3. Changes in Sleep End Points in the Time-Restricted Eating Groups Compared With Each Other After the 12-Week Intervention Considering the Daylight Time in the Analysis

End point	Early TRE vs. late TRE	Early TRE vs. self–selected TRE	Late TRE vs. self–selected TRE
	**Difference, mean (95% CI)[a]**	**Difference, mean (95% CI)[a]**	**Difference, mean (95% CI)[a]**
**Sleep**			
Onset, h	-0.2 (-0.5, 0.2)	-0.2 (-0.5, 0.2)	0.0 (-0.4, 0.3)
Offset, h	0.2 (-0.3, 0.6)	0.1 (-0.4, 0.5)	-0.1 (-0.5, 0.3)
Period, h[b]	0.3 (-0.1, 0.8)	0.2 (-0.2, 0.7)	-0.1 (-0.5, 0.3)
Total time, h[c]	0.3 (-0.1, 0.7)	0.2 (-0.2, 0.6)	0.0 (-0.4, 0.3)
Efficiency, %	0.3 (-1.8, 2.4)	1.0 (-1.2, 3.1)	0.7 (-1.5, 2.8)
Awakenings, No.	0.5 (-0.8, 1.8)	0.4 (-0.9, 1.8)	-0.1 (-1.4, 1.3)
Wake after sleep onset, h	0.0 (-0.1, 0.2)	0.0 (-0.2, 0.2)	-0.1 (-0.2, 0.1)
Sleep quality score[d]	-0.1 (-1.5, 1.3)	0.7 (-0.7, 2.2)	0.9 (-0.6, 2.3)

Abbreviations: CI, confidence interval; TRE, time-restricted eating.
[a] Data are presented as estimated mean differences and 95% CI between groups and were calculated by first computing the postintervention minus the preintervention values within each group; then, differences between the groups were computed as early TRE minus late TRE, early TRE minus self-selected TRE, and late TRE minus self-selected TRE. We derived a standardized score which had its minimum value at the Winter Solstice (i.e., -1), indicating the lowest daylight time in the year; and its maximum value at the Summer Solstice (i.e., +1), indicating the longest daylight time in the year. Sample size: early TRE, n = 49; late TRE, n = 52; self–selected TRE, n = 47. No statistically significant differences were detected in changes in sleep outcomes between the TRE groups considering the daylight time in the analysis.
[b] Time from sleep onset to sleep offset.
[c] Amount of time classified as sleep within the sleep period.
[d] Assessed using the Pittsburgh Sleep Quality Index (score range, 0-21 points, with higher scores indicating worse sleep quality).

© 2025 Clavero-Jimeno A et al. *JAMA Network Open*.

eTable 4. Sleep End Points at Baseline and After the 12-Week Intervention in Each Intervention Group Considering the Daylight Time in the Analysis

End point	UC (n = 49)	Early TRE (n = 49)	Late TRE (n = 52)	Self-selected TRE (n = 47)
	Mean (95% CI)[a]	Mean (95% CI)[a]	Mean (95% CI)[a]	Mean (95% CI)[a]
**Sleep**				
Onset				
Preintervention, h:min	00:24 (00:06, 00:36)	00:12 (00:00, 00:30)	00:12 (23:54, 00:24)	00:00 (23:42, 00:12)
Postintervention, h:min	00:24 (00:06, 00:36)	00:06 (23:54, 00:18)	00:12 (23:54, 00:24)	00:00 (23:48, 00:12)
Change, h	0.0 (-0.1, 0.2)	-0.1 (-0.3, 0.1)	0.0 (-0.2, 0.2)	0.0 (-0.1, 0.2)
Offset				
Preintervention, h:min	07:30 (07:18, 07:42)	07:24 (07:12, 07:42)	07:24 (07:06, 07:42)	07:24 (07:06, 07:36)
Postintervention, h:min	07:24 (07:12, 07:42)	07:24 (07:06, 07:42)	07:12 (06:54, 07:30)	07:18 (07:00, 07:30)
Change, h	0.0 (-0.2, 0.0)	0.0 (-0.3, 0.1)	-0.2 (-0.4, 0.0)	-0.1 (-0.3, -0.2)
Period, h[b]				
Preintervention	7.1 (6.8, 7.3)	7.2 (6.9, 7.4)	7.2 (7.0, 7.5)	7.4 (7.1, 7.6)
Postintervention	7.1 (6.8, 7.3)	7.3 (7.0, 7.6)	7.0 (6.8, 7.3)	7.3 (7.0, 7.5)
Change	0.0 (-0.3, 0.2)	0.1 (-0.1, 0.4)	-0.2 (-0.4, 0.0)	-0.1 (-0.3, 0.2)
Total time, h[c]				
Preintervention	6.2 (5.9, 6.4)	6.2 (5.9, 6.5)	6.3 (6.0, 6.6)	6.4 (6.1, 6.6)
Postintervention	6.0 (5.7, 6.2)	6.3 (6.0, 6.5)	6.1 (5.8, 5.3)	6.2 (5.9, 6.4)
Change	-0.2 (-0.4, 0.1)	0.1 (-0.16, 0.27)	-0.2 (-0.4, 0.0) *	0.2 (-0.4, 0.0)
Efficiency, %				
Preintervention	86.9 (85.2, 88.5)	86.4 (84.8, 87.9)	87.5 (85.9, 89.2)	86.4 (84.7, 88.1)
Postintervention	85.1 (83.4, 86.9)	85.9 (84.3, 87.6)	86.8 (85.1, 88.4)	85.0 (83.3, 86.7)
Change	-1.7 (-2.9, -0.5) *	-0.5 (-1.6, 0.7)	-0.7 (-1.9, 0.4)	-1.4 (-2.6, -0.2) *
Awakenings, No.				
Preintervention	13.4 (12.5, 14.4)	14.5 (13.5, 15.4)	14.0 (13.0, 14.9)	14.7 (13.7, 15.7)
Postintervention	12.7 (11.8, 13.6)	13.9 (12.7, 14.9)	12.9 (11.9, 13.8)	13.7 (12.7, 14.7)
Change	-0.7 (-1.4, 0.1)	-0.6 (-1.3, 0.2)	-1.1 (-1.8, -0.3) *	-1.0 (-1.8, -0.2) *
Wake after sleep onset, h				
Preintervention	0.9 (0.8, 1.04)	1.0 (0.8, 1.1)	0.9 (0.8, 1.0)	1.0 (0.9, 1.1)
Postintervention	1.0 (0.9, 1.2)	1.0 (0.9, 1.1)	0.9 (0.8, 1.1)	1.1 (1.0, 1.2)
Change	0.1 (0.0, 0.2) *	0.1 (0.0, 0.2)	0.0 (-0.1, 0.1)	0.1 (0.0, 0.2)
Sleep quality score[d]				
Preintervention	6.4 (5.6, 7.2)	5.9 (5.2, 6.6)	6.0 (5.2, 6.8)	7.2 (6.4, 8.0)
Postintervention	5.6 (4.9, 6.4)	5.6 (4.7, 6.4)	5.7 (4.9, 5.5)	6.1 (5.2, 6.9)
Change	-0.9 (-1.7, -0.1) *	-0.4 (-1.2, 0.4)	-0.3 (-1.1, 0.5)	-1.2 (-2.0, -0.4) *

Abbreviations: CI, confidence interval; TRE, time-restricted eating; UC, usual care.
[a] Asterisks (*) indicate statistically significant within-group differences as determined by 2-sided t-test ($P < .05$). No adjustments were made for multiple comparisons within groups. Changes were calculated as postintervention minus preintervention values. We derived a standardized score which had its minimum value at the Winter Solstice (i.e., -1), indicating the lowest daylight time in the year; and its maximum value at the Summer Solstice (i.e., +1), indicating the longest daylight time in the year.
[b] Time from sleep onset to sleep offset.
[c] Amount of time classified as sleep within the sleep period.
[d] Assessed using the Pittsburgh Sleep Quality Index (score range, 0-21 points, with higher scores indicating worse sleep quality).

© 2025 Clavero-Jimeno A et al. *JAMA Network Open*.

eTable 5. Changes in Sleep End Points in the Time-Restricted Eating Groups Compared With the Usual Care Group Following the 12-Week Intervention, After Excluding Participants Who Reported Taking Sleep Medication at Baseline

**End point**	**Early TRE vs. UC**	**Late TRE vs. UC**	**Self-selected TRE vs. UC**
	**Difference, mean (95% CI)[a]**	**Difference, mean (95% CI)[a]**	**Difference, mean (95% CI)[a]**
**Sleep**			
Onset, h	-0.3 (-0.7, 0.1)	-0.1 (-0.5, 0.3)	-0.1 (-0.5, 0.3)
Offset, h	-0.2 (-0.6, 0.3)	-0.2 (-0.6, 0.3)	-0.3 (-0.7, 0.3)
Period, h[b]	0.1 (-0.4, 0.6)	-0.1 (-0.5, 0.4)	-0.2 (-0.7, 0.4)
Total time, h[c]	0.2 (-0.2, 0.7)	0.0 (-0.4, 0.4)	0.0 (-0.5, 0.4)
Efficiency, %	2.0 (-0.4, 4.5)	1.0 (-1.4, 3.4)	1.2 (-1.2, 3.4)
Awakenings, No.	0.1 (-1.4, 1.6)	-0.1 (-1.6, 1.4)	-0.5 (-2.0, 1.4)
Wake after sleep onset, h	-0.1 (-0.3, 0.1)	-0.1 (-0.3, 0.1)	-0.1 (-0.3, 0.1)
Sleep quality score[d]	0.7 (-0.9, 2.2)	0.6 (-0.9, 2.1)	0.0 (-1.6, 2.1)

Abbreviations: CI, confidence interval; TRE, time-restricted eating; UC, usual care.

[a] Data are presented as estimated mean differences and 95% CI between groups and were calculated by first computing the postintervention minus the preintervention values within each group; then, differences between the groups were computed as early TRE minus UC, late TRE minus UC, and self-selected TRE minus UC. Sample size: UC, n = 38; early TRE, n = 40; late TRE, n = 45; self–selected TRE, n = 38. No statistically significant differences were detected in changes in sleep outcomes in the TRE groups compared to the UC group after excluding those participants who reported taking sleep medication at baseline.

[b] Time from sleep onset to sleep offset.

[c] Amount of time classified as sleep within the sleep period.

[d] Assessed using the Pittsburgh Sleep Quality Index (score range, 0-21 points, with higher scores indicating worse sleep quality).

© 2025 Clavero-Jimeno A et al. *JAMA Network Open*.

eTable 6. Changes in Sleep End Points in the Time-Restricted Eating Groups Compared With Each Other After the 12-Week Intervention, After Excluding Participants Who Reported Taking Sleep Medication at Baseline

End point	Early TRE vs. late TRE	Early TRE vs. self–selected TRE	Late TRE vs. self–selected TRE
	Difference, mean (95% CI)[a]	Difference, mean (95% CI)[a]	Difference, mean (95% CI)[a]
**Sleep**			
Onset, h	-0.2 (-0.6, 0.2)	-0.2 (-0.6, 0.2)	0.0 (-0.4, 0.4)
Offset, h	0.0 (-0.4, 0.4)	0.1 (-0.4, 0.6)	0.1 (-0.3, 0.6)
Period, h[b]	0.2 (-0.3, 0.6)	0.3 (-0.2, 0.7)	0.1 (-0.4, 0.6)
Total time, h[c]	0.2 (-0.2, 0.6)	0.3 (-0.2, 0.7)	0.1 (-0.3, 0.5)
Efficiency, %	1.1 (-1.3, 3.4)	0.8 (-1.6, 3.2)	-0.3 (-2.6, 2.1)
Awakenings, No.	0.2 (-1.2, 1.7)	0.6 (-0.9, 2.1)	0.4 (-1.1, 1.9)
Wake after sleep onset, h	0.0 (-0.2, 0.1)	0.0 (-0.2, 0.2)	0.1 (-0.1, 0.2)
Sleep quality score[d]	0.1 (-1.3, 1.6)	0.7 (-0.8, 2.2)	0.6 (-0.9, 2.0)

Abbreviations: CI, confidence interval; TRE, time-restricted eating.
[a] Data are presented as estimated mean differences and 95% CI between groups and were calculated by first computing the postintervention minus the preintervention values within each group; then, differences between the groups were computed as early TRE minus late TRE, early TRE minus self-selected TRE, and late TRE minus self-selected TRE. Sample size: early TRE, n = 40; late TRE, n = 45; self-selected TRE, n = 38. No statistically significant differences were detected in changes in sleep outcomes between the TRE groups after excluding those participants who reported taking sleep medication at baseline.
[b] Time from sleep onset to sleep offset.
[c] Amount of time classified as sleep within the sleep period.
[d] Assessed using the Pittsburgh Sleep Quality Index (score range, 0-21 points, with higher scores indicating worse sleep quality).

© 2025 Clavero-Jimeno A et al. *JAMA Network Open*.

eTable 7. Sleep End Points at Baseline and Following the 12-Week Intervention in Each Intervention Group, After Excluding Participants Who Reported Taking Sleep Medication at Baseline

End point	UC (n = 38)	Early TRE (n = 40)	Late TRE (n = 45)	Self-selected TRE (n = 38)
	Mean (95% CI)[a]	Mean (95% CI)[a]	Mean (95% CI)[a]	Mean (95% CI)[a]
**Sleep**				
Onset				
Preintervention, h:min	00:24 (00:06, 00:24)	00:12 (00:00, 00:30)	00:06 (23:54, 00:18)	00:06 (23:48, 00:18)
Postintervention, h:min	00:30 (00:12, 00:48)	00:06 (23:48, 00:24)	00:06 (23:54, 00:24)	00:06 (23:48, 00:18)
Change, h	0.1 (-0.1, 0.3)	-0.2 (-0.4, 0.1)	0.0 (-0.2, 0.2)	0.0 (-0.2, 0.2)
Offset				
Preintervention, h:min	07:30 (07:12, 07:48)	07:30 (07:12, 07:42)	07:24 (07:12, 07:36)	07:30 (07:12, 07:42)
Postintervention, h:min	07:30 (07:12, 07:48)	07:18 (07:06, 07:36)	07:12 (07:00, 31.5)	07:12 (07:54, 07:30)
Change, h	0.0 (-0.3, 0.2)	-0.1 (-0.4, 0.1)	-0.1 (-0.4, 0.1)	-0.3 (-0.5, 0.0) *
Period, h[b]				
Preintervention	7.1 (6.9, 7.4)	7.2 (7.0, 7.5)	7.3 (7.1, 7.5)	7.4 (7.2, 7.7)
Postintervention	7.0 (6.8, 7.3)	7.2 (7.0, 7.5)	7.1 (6.9, 7.4)	7.1 (6.9, 7.4)
Change	-0.1 (-0.4, 0.2)	0.0 (-0.3, 0.2)	-0.2 (-0.4, 0.1)	-0.3 (-0.5, 0.0) *
Total time, h[c]				
Preintervention	6.2 (5.9, 6.5)	6.2 (5.9, 6.5)	6.4 (6.2, 6.7)	6.4 (6.2, 6.7)
Postintervention	5.9 (5.7, 6.2)	6.2 (5.9, 6.5)	6.2 (5.9, 6.5)	6.2 (5.9, 6.4)
Change	-0.2 (-0.5, 0.0) *	0.0 (-0.2, 0.2)	-0.2 (-0.4, 0.0)	-0.3 (-0.5, -0.1) *
Efficiency, %				
Preintervention	86.6 (84.7, 88.5)	85.5 (83.7, 87.3)	87.8 (86.0, 89.5)	86.5 (84.6, 88.3)
Postintervention	84.7 (82.8, 86.6)	85.6 (83.8, 87.5)	86.8 (85.1, 88.6)	85.8 (83.9, 87.7)
Change	-1.9 (-3.2, -0.6) *	0.1 (-1.2, 1.4)	-0.9 (-2.2, 0.3)	-0.7 (-2.0, 0.6)
Awakenings, No.				
Preintervention	13.7 (12.7, 14.7)	14.9 (13.9, 15.9)	13.9 (13.0, 14.8)	14.6 (13.6, 15.6)
Postintervention	12.9 (11.9, 14.0)	14.3 (13.3, 15.3)	13.1 (12.1, 14.0)	13.4 (12.4, 14.4)
Change	-0.7 (-1.5, 0.1)	-0.6 (-1.4, 0.2)	-0.8 (-1.6, -0.1) *	-1.2 (-2.0, -0.4) *
Wake after sleep onset, h				
Preintervention	0.9 (0.8, 1.1)	1.0 (0.9, 1.2)	0.9 (0.8, 1.0)	1.0 (0.9, 1.1)
Postintervention	1.1 (0.9, 1.2)	1.0 (0.9, 1.2)	0.9 (0.8, 1.1)	1.0 (0.9, 1.1)
Change	0.1 (0.0, 0.2) *	0.0 (-0.1, 0.1)	0.1 (-0.1, 0.1)	0.0 (-0.1, 0.1)
Sleep quality score[d]				
Preintervention	5.8 (4.9, 6.6)	5.6 (4.7, 6.4)	5.6 (4.8, 6.4)	6.5 (5.7, 7.4)
Postintervention	5.1 (4.2, 6.0)	5.6 (4.7, 6.4)	5.5 (4.7, 6.3)	5.8 (4.9, 6.7)
Change	-0.7 (-1.5, 0.1)	0.0 (-0.8, 0.8)	-0.1 (-0.9, 0.6)	-0.7 (-1.5, 0.1)

Abbreviations: CI, confidence interval; TRE, time-restricted eating; UC, usual care.
[a] Asterisks (*) indicate statistically significant within-group differences as determined by 2-sided t-test ($P < .05$). No adjustments were made for multiple comparisons within groups. Changes were calculated as postintervention minus preintervention values.
[b] Time from sleep onset to sleep offset.
[c] Amount of time classified as sleep within the sleep period.
[d] Assessed using the Pittsburgh Sleep Quality Index (score range, 0-21 points, with higher scores indicating worse sleep quality).

© 2025 Clavero-Jimeno A et al. *JAMA Network Open*.

eFigure 1. Study Flow Chart

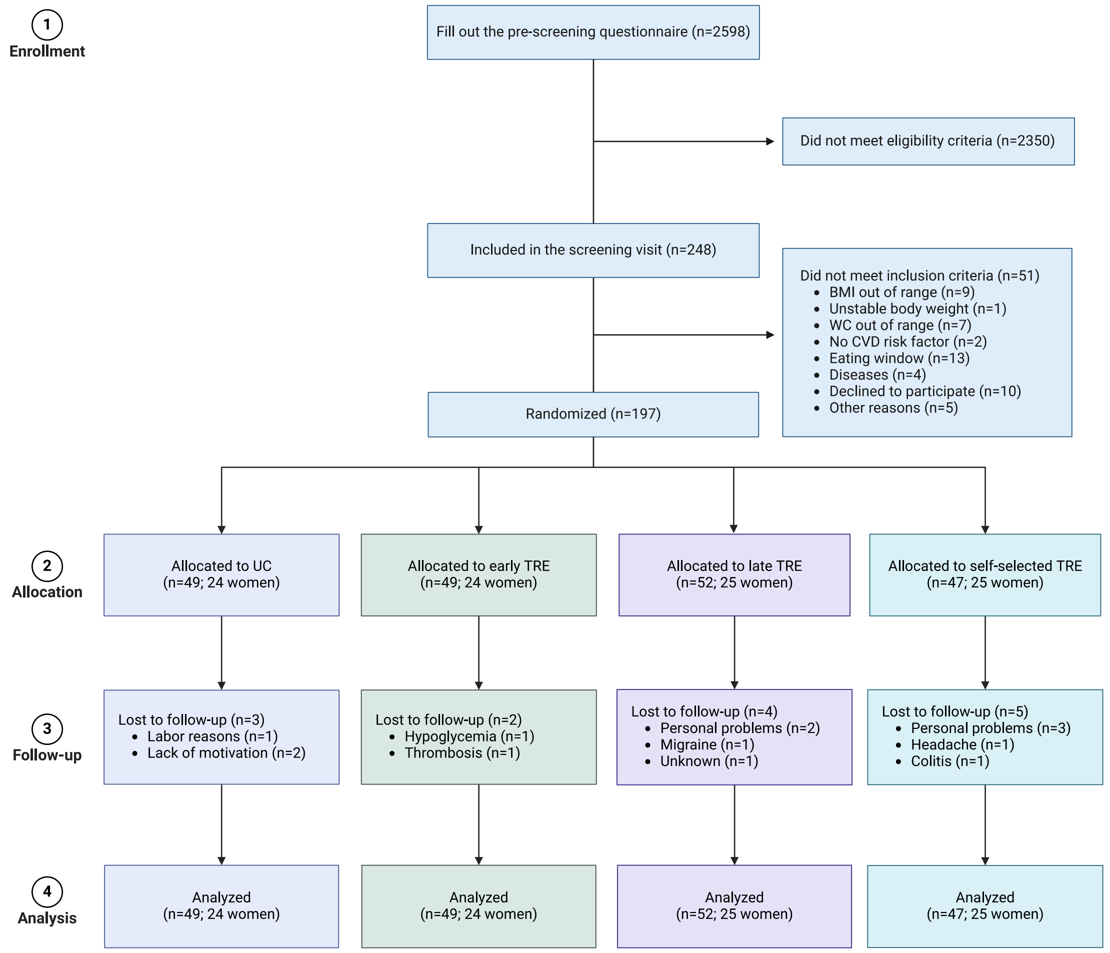

Usual care (UC), early time-restricted eating (TRE), late TRE, or self-selected TRE groups. BMI, body mass index; CVD, cardiovascular disease; WC, waist circumference. Created in BioRender. Ruiz, J. (2024) BioRender.com/t37h141

© 2025 Clavero-Jimeno A et al. *JAMA Network Open*.

eFigure 2. Changes in General Health Across All Groups After the 12-Week Intervention

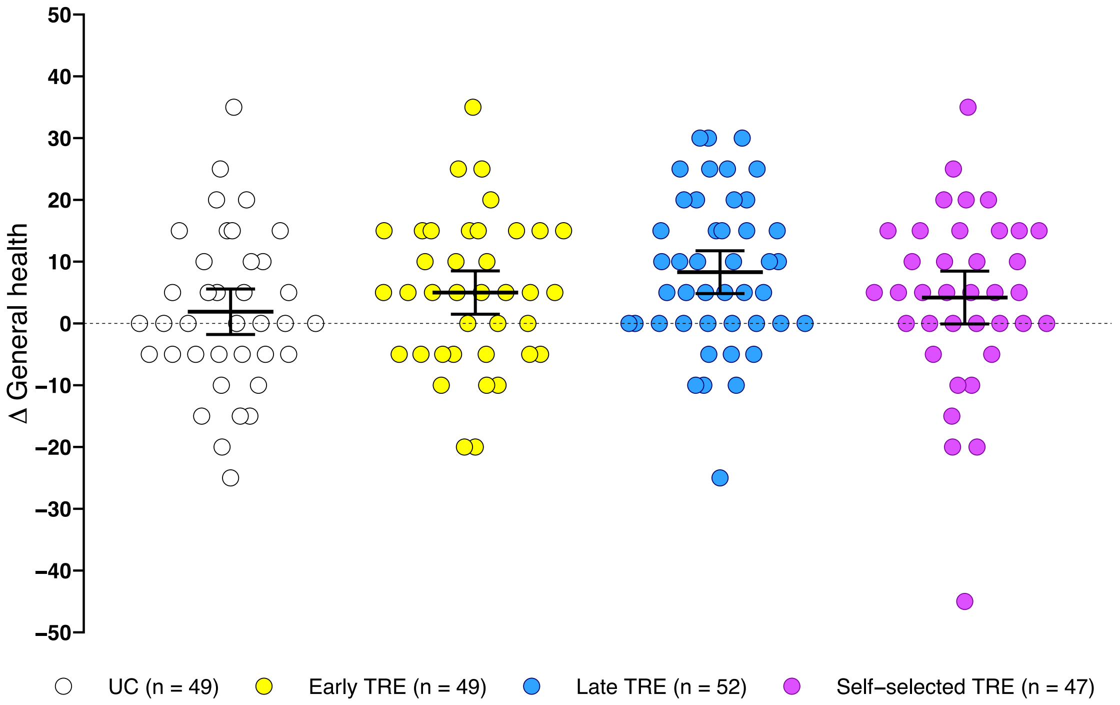

Changes were calculated as postintervention minus preintervention values. No statistically significant differences in changes in general health were detected across all groups after the intervention. General health was assessed using the Rand 36-Item Short Form Health Survey (score range, 0-100 points, with higher scores reflecting better quality of life). TRE indicates time-restricted eating; UC, usual care. Circles represent individual participants' scores; horizontal bars, raw means; whiskers, 95% CIs.

© 2025 Clavero-Jimeno A et al. *JAMA Network Open*.

eTable 8. Baseline Characteristics of All Randomized Participants by Sex and Intervention Group

**Men**[a]	**UC (n = 25)**	**Early TRE (n = 25)**	**Late TRE (n = 27)**	**Self-selected TRE (n = 22)**
Age, y				
Mean (SD)	47.7 (6.4)	47.8 (7.5)	47.8 (6.6)	45.7 (5.3)
30–39	2 (8.0)	5 (20.0)	4 (14.8)	3 (13.6)
40–49	15 (60.0)	7 (28.0)	11 (40.7)	13 (59.1)
50–60	8 (32.0)	13 (52.0)	12 (44.4)	6 (27.3)
**Anthropometry and body composition**				
Weight, mean (SD), kg	101.8 (14.9)	106.3 (13.6)	101.6 (14.8)	99.8 (12.5)
Height, mean (SD), cm	176.6 (5.8)	176.5 (7.1)	176.3 (6.8)	176.7 (6.2)
BMI, mean (SD)	32.5 (3.2)	34.0 (2.9)	32.6 (3.6)	31.9 (3.0)
Fat-free mass, mean (SD), kg	63.2 (8.3)	65.8 (7.6)	63.2 (7.2)	63.3 (6.5)
Fat mass				
Mean (SD), kg	37.4 (7.5)	39.5 (8.0)	37.6 (9.4)	35.3 (7.1)
Mean (SD), %	37.0 (3.9)	37.3 (4.1)	36.8 (5.1)	35.6 (4.0)
**Medications**[b]				
Sleep medication[c]	4 (16.0)	3 (12.0)	4 (14.8)	4 (18.2)
Antihypertensives	5 (20.0)	5 (20.0)	5 (18.5)	2 (9.1)
β-Blockers	0	0	0	0
Antidepressants	0	0	0	0
Anxyolitics	1 (4.0)	1 (4.0)	0	1 (4.5)
Statins	3 (12.0)	3 (12.0)	2 (7.4)	2 (9.1)
**Sleep**				
Onset, mean (SD), h:min	00:36 (00:48)	00:06 (00:54)	00:06 (00:48)	00:00 (00:54)
Offset, mean (SD), h:min	07:36 (00:42)	07:24 (01:06)	07:24 (00:48)	07:12 (00:48)
Period, mean (SD), h[d]	7.0 (0.8)	7.3 (1.0)	7.3 (0.6)	7.2 (0.6)
Total time, mean (SD), h[e]	6.0 (0.9)	6.2 (1.1)	6.3 (0.6)	6.2 (0.7)
Efficiency, mean (SD), %	85.1 (7.6)	84.0 (6.9)	86.0 (5.7)	86.2 (5.1)
Awakenings, mean (SD), No.	13.3 (3.2)	15.8 (3.8)	14.5 (3.2)	14.1 (2.3)
Wake after sleep onset, mean (SD), h	1.0 (0.5)	1.1 (0.4)	1.0 (0.5)	1.0 (0.3)
Sleep quality score, mean (SD)[f]	6.0 (2.5)	5.1 (2.6)	6.0 (2.7)	6.6 (2.8)
**Mood**				
Depression score, mean (SD)[g]	1.8 (1.9)	1.4 (1.8)	2.3 (2.8)	2.1 (2.7)
Minimal	22 (88.0)	23 (92.0)	22 (81.5)	19 (86.4)
Light	3 (12.0)	2 (8.0)	3 (11.1)	2 (9.1)
Moderate	0	0	2 (7.4)	1 (4.5)
Severe	0	0	0	0
State anxiety score, mean (SD)[h]	14.6 (9.6)	14.2 (10.6)	12.9 (8.6)	16.1 (9.3)
Low	20 (80.0)	18 (72.0)	22 (81.5)	17 (77.3)
Moderate	4 (16.0)	7 (28.0)	5 (18.5)	4 (18.2)
High	1 (4.0)	0	0	1 (4.5)
Trait anxiety score, mean (SD)[h]	15.8 (8.6)	15.8 (8.8)	16.3 (7.8)	18.8 (10.6)
Low	21 (84.0)	18 (72.0)	22 (81.5)	16 (72.7)
Moderate	3 (12.0)	7 (28.0)	4 (14.8)	5 (22.7)
High	1 (4.0)	0	1 (3.7)	1 (4.5)
Stress score, mean (SD)[i]	23.6 (8.8)	21.0 (8.5)	22.3 (8.5)	25.3 (6.3)
**Quality-of-life scores, mean (SD)**[j]				

© 2025 Clavero-Jimeno A et al. *JAMA Network Open*.

eTable 8. Baseline Characteristics of All Randomized Participants by Sex and Intervention Group (continued)

**Men[a]**	**UC (n = 25)**	**Early TRE (n = 25)**	**Late TRE (n = 27)**	**Self-selected TRE (n = 22)**
Physical functioning	89.6 (9.5)	93.6 (5.7)	88.1 (11.4)	92.3 (8.8)
Role limitations				
Due to physical health	89.0 (26.1)	94.0 (16.6)	89.8 (19.9)	86.4 (25.3)
Due to emotional problems	96.0 (14.7)	92.0 (24.1)	96.3 (14.1)	69.7 (43.5)
Vitality	56.0 (20.4)	59.2 (22.5)	49.2 (17.7)	49.8 (16.1)
Social functioning	73.9 (23.5)	74.9 (24.3)	72.7 (24.3)	67.0 (25.9)
Bodily pain	82.7 (16.1)	81.7 (20.8)	78.6 (18.5)	79.8 (18.8)
General health	65.6 (16.0)	66.4 (14.6)	62.4 (14.6)	66.4 (15.0)
**Women[a]**	**UC (n = 24)**	**Early TRE (n = 24)**	**Late TRE (n = 25)**	**Self–selected TRE (n = 25)**
Age, y				
Mean (SD)	45.7 (5.6)	46.5 (4.5)	48.2 (7.3)	44.8 (6.3)
30–39	2 (8.3)	3 (12.5)	4 (16.0)	4 (16.0)
40–49	16 (66.7)	15 (62.5)	8 (32.0)	16 (64.0)
50–60	6 (25.0)	6 (25.0)	13 (52.0)	5 (20.0)
**Anthropometry and body composition**				
Weight, mean (SD), kg	90.3 (11.4)	88.9 (11.7)	85.2 (11.1)	88.2 (12.7)
Height, mean (SD), cm	162.2 (5.1)	162.7 (5.4)	162.2 (6.0)	163.8 (5.3)
BMI, mean (SD)	34.3 (4.0)	33.5 (3.8)	32.3 (3.2)	32.8 (3.6)
Fat-free mass, mean (SD), kg	47.5 (6.9)	46.4 (5.4)	44.1 (4.9)	45.9 (5.8)
Fat mass				
Mean (SD), kg	42.0 (7.9)	41.4 (7.8)	40.5 (7.3)	41.7 (7.9)
Mean (SD), %	46.8 (5.2)	46.9 (4.2)	47.7 (4.1)	47.3 (4.0)
**Medications[b]**				
Sleep medication[c]	7 (29.2)	6 (25.0)	3 (12.0)	5 (20.0)
Antihypertensives	3 (12.5)	3 (12.5)	4 (16.0)	7 (28.0)
β-Blockers	1 (4.2)	0	1 (4.0)	0
Antidepressants	4 (16.7)	1 (4.2)	1 (4.0)	4 (16.0)
Anxyolitics	3 (12.5)	3 (12.5)	0	2 (8.0)
Statins	0	1 (4.2)	2 (8.0)	0
**Sleep**				
Onset, mean (SD), h:min	00:06 (01:00)	00:24 (00:36)	00:18 (00:48)	23:54 (00:48)
Offset, mean (SD), h:min	07:24 (00:54)	07:36 (00:48)	07:30 (00:48)	07:36 (00:48)
Period, mean (SD), h[d]	7.2 (1.0)	7.2 (0.9)	7.2 (0.7)	7.6 (0.8)
Total time, mean (SD), h[e]	6.4 (1.0)	6.4 (1.0)	6.4 (0.6)	6.6 (0.8)
Efficiency, mean (SD), %	88.4 (4.3)	88.8 (4.5)	89.2 (3.6)	86.6 (5.6)
Awakenings, mean (SD), No.	13.5 (3.7)	13.1 (3.0)	13.3 (2.9)	15.1 (3.7)
Wake after sleep onset, mean (SD), h	0.8 (0.3)	0.8 (0.3)	0.8 (0.3)	1.0 (0.4)
Sleep quality score, mean (SD)[f]	6.9 (3.5)	6.6 (2.8)	5.8 (2.8)	7.7 (3.3)
**Mood**				
Depression score, mean (SD)[g]	2.5 (2.8)	2.7 (2.4)	2.1 (2.2)	2.9 (2.6)
Minimal	17 (70.8)	17 (70.8)	20 (80.0)	16 (64.0)
Light	7 (29.2)	7 (29.2)	5 (20.0)	9 (36.0)
Moderate	0	0	0	0
Severe	0	0	0	0

© 2025 Clavero-Jimeno A et al. *JAMA Network Open*.

eTable 8. Baseline Characteristics of All Randomized Participants by Sex and Intervention Group (continued)

**Women**[a]	**UC (n = 24)**	**Early TRE (n = 24)**	**Late TRE (n = 25)**	**Self–selected TRE (n = 25)**
State anxiety score, mean (SD)[h]	16.7 (12.5)	20.0 (11.5)	15.6 (9.5)	20.2 (10.2)
Low	17 (70.8)	13 (54.2)	17 (68.0)	16 (64.0)
Moderate	6 (25.0)	10 (41.7)	8 (32.0)	9 (36.0)
High	1 (4.2)	1 (4.2)	0	0
Trait anxiety score, mean (SD)[h]	19.2 (11.7)	22.6 (10.2)	18.7 (8.4)	23.1 (11.0)
Low	13 (54.2)	13 (54.2)	13 (52.0)	12 (48.0)
Moderate	10 (41.7)	11 (45.8)	12 (48.0)	11 (44.0)
High	1 (4.2)	0	0	2 (8.0)
Stress score, mean (SD)[i]	24.4 (8.4)	25.4 (8.3)	24.7 (7.7)	26.7 (7.4)
**Quality-of-life scores, mean (SD)**[j]				
Physical functioning	87.3 (13.8)	89.8 (7.5)	87.0 (10.6)	84.0 (15.9)
Role limitations				
Due to physical health	80.2 (38.3)	86.4 (29.6)	84.0 (29.7)	73.0 (42.0)
Due to emotional problems	84.7 (31.1)	74.2 (38.4)	92.0 (17.4)	78.7 (35.8)
Vitality	52.7 (17.6)	47.0 (12.5)	48.0 (14.3)	47.2 (13.3)
Social functioning	65.2 (20.0)	66.0 (22.9)	72.6 (23.4)	69.2 (22.4)
Bodily pain	76.9 (17.5)	82.5 (15.0)	77.4 (18.8)	60.0 (25.6)
General health	67.1 (17.2)	65.0 (19.9)	60.4 (18.0)	57.2 (18.9)

Abbreviations: BMI, body mass index (calculated as weight in kilograms divided by height in 1 meters squared); TRE, time-restricted eating; UC, usual care.
[a] Data are presented as number (percentage) of participants unless otherwise indicated.
[b] Participants could take more than 1 medication.
[c] One man (4.0%) and 1 woman (4.2%) in the UC group, 3 women (12.5%) in the early TRE group, and 1 man (4.5%) in the self-selected TRE group took prescribed sleep medication, while the remaining men and women used over-the-counter options.
[d] Time from sleep onset to sleep offset.
[e] Amount of time classified as sleep within the sleep period.
[f] Assessed using the Pittsburgh Sleep Quality Index (score range, 0-21 points, with higher scores indicating worse sleep quality).
[g] Assessed using the Beck Depression Inventory Fast Screen (score range, 0-21 points, with higher scores reflecting more depressive symptoms). Depression severity is categorized based on the following scores: 0 to 3 (minimal), 4 to 8 (light), 9 to 12 (moderate), and 13 to 21 (severe).
[h] Assessed using the State-Trait Anxiety Inventory (total score range, 0-60 points for state anxiety and trait anxiety, with higher scores reflecting greater anxiety). State or trait anxiety severity is categorized based on the following scores: 0 to 20 (low), 21 to 40 (moderate), and 41 to 60 (high).
[i] Assessed using the Perceived Stress Scale (score range, 0-56 points, with higher scores indicating greater perceived stress).
[j] Assessed using the Rand 36-Item Short Form Health Survey (score range, 0-100 points, with higher scores reflecting better quality of life).

© 2025 Clavero-Jimeno A et al. *JAMA Network Open*.

eTable 9. Changes in Sleep, Mood, and Quality-of-Life End Points in the Time-Restricted Eating Groups Compared With the Usual Care Group After the 12-Week Intervention, Divided by Sex

Men			
**End point**	**Early TRE vs. UC**	**Late TRE vs. UC**	**Self-selected TRE vs. UC**
	**Difference, mean (95% CI)[a]**	**Difference, mean (95% CI)[a]**	**Difference, mean (95% CI)[a]**
**Sleep**			
Onset, h	0.0 (-0.5, 0.6)	0.2 (-0.3, 0.7)	0.1 (-0.4, 0.7)
Offset, h	0.0 (-0.7, 0.7)	-0.2 (-0.9, 0.4)	-0.2 (-0.9, 0.5)
Period, h[b]	-0.1 (-0.7, 0.6)	-0.4 (-1.1, 0.2)	-0.3 (-1.0, 0.3)
Total time, h[c]	0.1 (-0.5, 0.7)	-0.2 (-0.8, 0.3)	-0.2 (-0.8, 0.4)
Efficiency, %	1.7 (-1.3, 4.7)	1.9 (-1.1, 4.9)	1.3 (-1.7, 4.3)
Awakenings, No.	-0.9 (-2.6, 0.8)	-1.2 (-2.9, 0.4)	-1.4 (-3.2, 0.2)
Wake after sleep onset, h	-0.1 (-0.3, 0.1)	-0.2 (-0.4, 0.0)	-0.1 (-0.4, 0.1)
Sleep quality score[d]	0.7 (-0.9, 2.4)	-0.1 (-1.7, 1.5)	-0.2 (-1.9, 1.5)
**Mood scores**			
Depression[e]	0.7 (-0.8, 2.2)	-0.1 (-1.5, 1.3)	0.4 (-1.0, 1.9)
State anxiety[f]	-0.8 (-7.8, 6.2)	-2.8 (-9.5, 3.9)	-0.7 (-7.8, 6.4)
Trait anxiety[f]	0.4 (-4.7, 5.5)	-1.0 (-5.9, 3.9)	1.6 (-3.6, 6.8)
Stress[g]	3.4 (-1.8, 8.6)	1.8 (-3.2, 6.8)	0.2 (-5.0, 5.4)
**Quality-of-life scores[h]**			
Physical functioning	-0.3 (-7.7, 7.1)	4.2 (-3.1, 11.4)	2.4 (-5.1, 9.9)
Role limitations			
Due to physical health	3.4 (-18.7, 25.6)	12.2 (-9.5, 33.9)	4.6 (-18.0, 27.1)
Due to emotional problems	12.6 (-8.0, 33.2)	12.9 (-7.3, 33.1)	31.9 (10.9, 52.8) *
Vitality	1.0 (-8.9, 10.9)	4.7 (-5.1, 14.5)	5.7 (-4.3, 15.8)
Social functioning	-3.0 (-11.7, 5.6)	0.8 (-7.7, 9.3)	-1.0 (-9.8, 7.8)
Bodily pain	2.5 (-14.5, 19.6)	9.3 (-7.4, 25.9)	6.4 (-10.9, 23.8)
General health	1.5 (-8.8, 11.9)	6.2 (-4.0, 16.3)	-1.2 (-11.7, 9.3)

Women			
**End point**	**Early TRE vs. UC**	**Late TRE vs. UC**	**Self-selected TRE vs. UC**
	**Difference, mean (95% CI)[a]**	**Difference, mean (95% CI)[a]**	**Difference, mean (95% CI)[a]**
**Sleep**			
Onset, h	-0.3 (-0.8, 0.1)	-0.2 (-0.7, 0.3)	-0.1 (-0.6, 0.4)
Offset, h	0.1 (-0.5, 0.6)	-0.1 (-0.6, 0.5)	0.1 (-0.5, 0.6)
Period, h[b]	0.4 (-0.2, 1.0)	0.1 (-0.5, 0.8)	0.2 (-0.4, 0.9)
Total time, h[c]	0.4 (-0.1, 0.9)	0.2 (-0.3, 0.7)	0.1 (-0.4, 0.7)
Efficiency, %	0.8 (-2.3, 3.8)	0.0 (-3.1, 3.0)	-0.7 (-3.7, 2.4)
Awakenings, No.	1.2 (-0.8, 3.3)	0.6 (-1.5, 2.6)	1.0 (-1.1, 3.1)
Wake after sleep onset, h	0.0 (-0.2, 0.3)	0.0 (-0.2, 0.3)	0.1 (-0.2, 0.3)
Sleep quality score[d]	0.1 (-2.4, 2.6)	1.1 (-1.4, 3.5)	-0.4 (-2.8, 2.1)
**Mood scores**			
Depression[e]	-0.3 (-2.2, 1.5)	-0.1 (-1.9, 1.7)	-0.5 (-2.4, 1.4)
State anxiety[f]	-1.5 (-9.6, 6.7)	-0.6 (-8.6, 7.5)	-0.5 (-8.8, 7.9)
Trait anxiety[f]	0.3 (-5.5, 6.1)	-0.8 (-6.4, 4.9)	-1.0 (-6.9, 5.0)
Stress[g]	0.6 (-5.4, 6.5)	-1.6 (-7.4, 4.3)	-0.6 (-6.7, 5.5)

© 2025 Clavero-Jimeno A et al. *JAMA Network Open*.

eTable 9. Changes in Sleep, Mood, and Quality-of-Life End Points in the Time-Restricted Eating Groups Compared With the Usual Care Group After the 12-Week Intervention, Divided by Sex (continued)

Women			
**End point**	**Early TRE vs. UC**	**Late TRE vs. UC**	**Self-selected TRE vs. UC**
	**Difference, mean (95% CI)[a]**	**Difference, mean (95% CI)[a]**	**Difference, mean (95% CI)[a]**
**Quality-of-life scores[h]**			
Physical functioning	-0.2 (-9.6, 9.2)	2.0 (-7.0, 11.0)	1.1 (-8.3, 10.5)
Role limitations			
Due to physical health	-22.4 (-55.2, 10.4)	-6.6 (-38.3, 25.1)	-0.3 (-33.2, 32.6)
Due to emotional problems	1.7 (-30.1, 33.5)	1.5 (-29.2, 32.2)	2.7 (-29.1, 34.6)
Vitality	1.0 (-11.4, 13.3)	7.3 (-4.5, 19.1)	8.4 (-3.9, 20.8)
Social functioning	-6.1 (-20.2, 7.9)	-2.4 (-15.9, 11.0)	-7.4 (-21.5, 6.6)
Bodily pain	-9.0 (-27.4, 9.4)	-4.5 (-22.2, 13.1)	14.2 (-4.2, 32.6)
General health	5.3 (-6.4, 17.0)	7.6 (-3.6, 18.9)	7.2 (-4.5, 18.9)

Abbreviations: CI, confidence interval; TRE, time-restricted eating; UC, usual care.

[a] Asterisks (*) indicate statistically significant differences between each TRE group and the UC group as determined by post-hoc Tukey correction for multiple comparisons (*P* < .05). Data are presented as estimated mean differences and 95% CI between groups and were calculated by first computing the postintervention minus the preintervention values within each group; then, differences between the groups were computed as early TRE minus UC, late TRE minus UC, and self-selected TRE minus UC. Sample size: UC, n = 25 men and 24 women; early TRE, n = 25 men and 24 women; late TRE, n = 27 men and 25 women; self–selected TRE, n = 22 men and 25 women.

[b] Time from sleep onset to sleep offset.

[c] Amount of time classified as sleep within the sleep period.

[d] Assessed using the Pittsburgh Sleep Quality Index (score range, 0-21 points, with higher scores indicating worse sleep quality).

[e] Assessed using the Beck Depression Inventory Fast Screen (score range, 0-21 points, with higher scores reflecting more depressive symptoms).

[f] Assessed using the State-Trait Anxiety Inventory (total score range, 0-60 points for state anxiety and trait anxiety, with higher scores reflecting greater anxiety).

[g] Assessed using the Perceived Stress Scale (score range, 0-56 points, with higher scores indicating greater perceived stress).

[h] Assessed using the Rand 36-Item Short Form Health Survey (score range, 0-100 points, with higher scores reflecting better quality of life).

© 2025 Clavero-Jimeno A et al. *JAMA Network Open*.

eTable 10. Changes in Sleep, Mood, and Quality-of-Life End Points in the Time-Restricted Eating Groups Compared With Each Other After the 12-Week Intervention, Divided by Sex

Men			
**End point**	**Early TRE vs. late TRE**	**Early TRE vs. self–selected TRE**	**Late TRE vs. self–selected TRE**
	**Difference, mean (95% CI)[a]**	**Difference, mean (95% CI)[a]**	**Difference, mean (95% CI)[a]**
**Sleep**			
Onset, h	-0.2 (-0.7, 0.3)	-0.1 (-0.7, 0.4)	0.0 (-0.5, 0.6)
Offset, h	0.2 (-0.4, 0.9)	0.2 (-0.5, 0.9)	0.0 (-0.7, 0.7)
Period, h[b]	0.4 (-0.3, 1.0)	0.3 (-0.3, 0.9)	-0.1 (-0.7, 0.6)
Total time, h[c]	0.3 (-0.3, 0.9)	0.3 (-0.3, 0.9)	0.0 (-0.6, 0.6)
Efficiency, %	-0.2 (-3.2, 2.8)	0.4 (-2.6, 3.5)	0.6 (-2.4, 3.7)
Awakenings, No.	0.3 (-1.4, 2.0)	0.5 (-1.2, 2.3)	0.2 (-1.5, 1.9)
Wake after sleep onset, h	0.1 (-0.1, 0.3)	0.1 (-0.2, 0.3)	0.0 (-0.2, 0.2)
Sleep quality score[d]	0.8 (-0.8, 2.5)	0.9 (-0.8, 2.7)	0.1 (-1.6, 1.8)
**Mood scores**			
Depression[e]	0.8 (-0.6, 2.2)	0.2 (-1.2, 1.7)	-0.6 (-2.0, 0.8)
State anxiety[f]	2.0 (-4.7, 8.7)	-0.1 (-7.1, 6.9)	-2.1 (-8.9, 4.7)
Trait anxiety[f]	1.4 (-3.4, 6.3)	-1.2 (-6.3, 3.9)	-2.6 (-7.6, 2.3)
Stress[g]	1.6 (-3.3, 6.5)	3.2 (-2.0, 8.4)	1.6 (-3.4, 6.6)
**Quality-of-life scores[h]**			
Physical functioning	-4.5 (-11.7, 2.7)	-2.7 (-10.2, 4.7)	1.7 (-5.6, 9.0)
Role limitations			
Due to physical health	-8.8 (-30.3, 12.8)	-1.1 (-23.6, 21.3)	7.6 (-14.3, 29.6)
Due to emotional problems	-0.3 (-20.3, 19.7)	-19.3 (-40.1, 1.5)	-19.0 (-39.3, 1.4)
Vitality	-3.7 (-13.4, 6.0)	-4.8 (-14.7, 5.2)	-1.1 (-10.9, 8.7)
Social functioning	-3.8 (-12.2, 4.6)	-2.1 (-10.8, 6.6)	1.7 (-6.8, 10.3)
Bodily pain	-6.7 (-23.2, 9.8)	-3.9 (-21.1, 13.3)	2.8 (-14.0, 19.7)
General health	-4.6 (-14.7, 5.4)	2.7 (-7.7, 13.1)	7.4 (-2.8, 17.5)

Women			
**End point**	**Early TRE vs. late TRE**	**Early TRE vs. self–selected TRE**	**Late TRE vs. self–selected TRE**
	**Difference, mean (95% CI)[a]**	**Difference, mean (95% CI)[a]**	**Difference, mean (95% CI)[a]**
**Sleep**			
Onset, h	-0.1 (-0.6, 0.4)	-0.2 (-0.7, 0.3)	-0.1 (-0.6, 0.4)
Offset, h	0.1 (-0.4, 0.6)	0.0 (-0.6, 0.5)	-0.1 (-0.7, 0.4)
Period, h[b]	0.2 (-0.4, 0.8)	0.2 (-0.5, 0.8)	-0.1 (-0.7, 0.6)
Total time, h[c]	0.2 (-0.3, 0.7)	0.2 (-0.2, 0.8)	0.0 (-0.5, 0.5)
Efficiency, %	0.8 (-2.2, 3.8)	1.4 (-1.6, 4.4)	0.6 (-2.4, 3.6)
Awakenings, No.	0.7 (-1.3, 2.7)	0.2 (-1.8, 2.2)	-0.5 (-2.5, 1.6)
Wake after sleep onset, h	0.0 (-0.2, 0.2)	-0.1 (-0.3, 0.2)	-0.1 (-0.3, 0.2)
Sleep quality score[d]	-1.0 (-3.4, 1.4)	0.5 (-2.0, 2.9)	1.4 (-0.9, 3.8)
**Mood scores**			
Depression[e]	-0.2 (-2.0, 1.5)	0.1 (-1.7, 2.0)	0.4 (-1.4, 2.2)
State anxiety[f]	-0.9 (-8.7, 6.9)	-1.0 (-9.2, 7.2)	-0.1 (-8.1, 7.9)
Trait anxiety[f]	1.1 (-4.4, 6.6)	1.3 (-4.5, 7.1)	0.2 (-5.4, 5.9)

© 2025 Clavero-Jimeno A et al. *JAMA Network Open*.

eTable 10. Changes in Sleep, Mood, and Quality-of-Life End Points in the Time-Restricted Eating Groups Compared With Each Other After the 12-Week Intervention, Divided by Sex (continued)

Women			
**End point**	**Early TRE vs. late TRE**	**Early TRE vs. self–selected TRE**	**Late TRE vs. self–selected TRE**
	**Difference, mean (95% CI)[a]**	**Difference, mean (95% CI)[a]**	**Difference, mean (95% CI)[a]**
Stress[g]	2.1 (-3.6, 7.8)	1.1 (-4.8, 7.1)	-1.0 (-6.8, 4.8)
**Quality-of-life scores[h]**			
Physical functioning	-2.2 (-11.2, 6.7)	-1.3 (-10.7, 8.1)	0.9 (-8.1, 9.9)
Role limitations			
Due to physical health	-15.8 (-47.3, 15.7)	-22.1 (-54.8, 10.6)	-6.3 (-37.9, 25.3)
Due to emotional problems	0.2 (-30.3, 30.7)	-1.0 (-32.7, 30.7)	-1.2 (-31.9, 29.4)
Vitality	-6.3 (-18.1, 5.5)	-7.5 (-19.8, 4.9)	-1.1 (-13.0, 10.7)
Social functioning	-3.7 (-17.1, 9.7)	1.3 (-12.7, 15.3)	5.0 (-8.4, 18.4)
Bodily pain	-4.4 (-22.0, 13.1)	-23.2 (-41.5, -4.8) *	-18.7 (-36.3, -1.1) *
General health	-2.3 (-13.5, 8.9)	-1.9 (-13.5, 9.8)	0.5 (-10.7, 11.7)

Abbreviations: CI, confidence interval; TRE, time-restricted eating.
[a] Asterisks (*) indicate statistically significant differences between TRE groups as determined by post-hoc Tukey correction for multiple comparisons (*P* < .05). Data are presented as estimated mean differences and 95% CI between groups and were calculated by first computing the postintervention minus the preintervention values within each group; then, differences between the groups were computed as early TRE minus late TRE, early TRE minus self-selected TRE, and late TRE minus self-selected TRE. Sample size: early TRE, n = 25 men and 24 women; late TRE, n = 27 men and 25 women; self–selected TRE, n = 22 men and 25 women.
[b] Time from sleep onset to sleep offset.
[c] Amount of time classified as sleep within the sleep period.
[d] Assessed using the Pittsburgh Sleep Quality Index (score range, 0-21 points, with higher scores indicating worse sleep quality).
[e] Assessed using the Beck Depression Inventory Fast Screen (score range, 0-21 points, with higher scores reflecting more depressive symptoms).
[f] Assessed using the State-Trait Anxiety Inventory (total score range, 0-60 points for state anxiety and trait anxiety, with higher scores reflecting greater anxiety).
[g] Assessed using the Perceived Stress Scale (score range, 0-56 points, with higher scores indicating greater perceived stress).
[h] Assessed using the Rand 36-Item Short Form Health Survey (score range, 0-100 points, with higher scores reflecting better quality of life).

© 2025 Clavero-Jimeno A et al. *JAMA Network Open*.

eFigure 3. Changes in Sleep Outcomes Across All Groups After the 12-Week Intervention, Divided by Sex

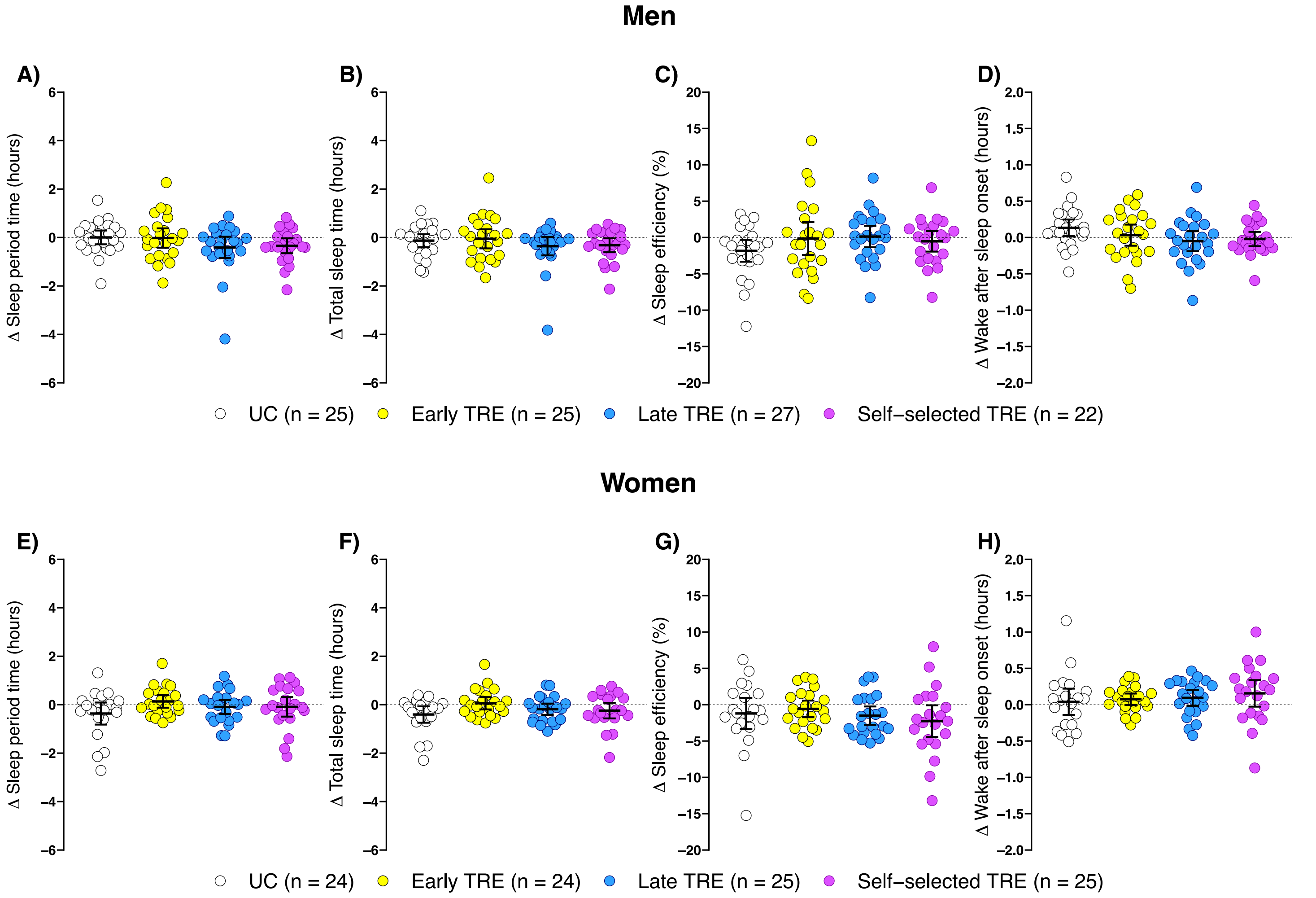

Changes in sleep period time (A and E), total sleep time (B and F), sleep efficiency (C and G) and wake after sleep onset (D and H) as measured by accelerometry over 14 days both at baseline and in the last 2 weeks of the 12-week intervention in men and women among the usual care (UC), early time–restricted eating (TRE), late TRE, and self–selected TRE groups. Changes were calculated as postintervention minus preintervention values. No statistically significant differences in changes in sleep outcomes were detected in men and women across all groups after the intervention. Circles represent individual participants' measures; horizontal bars, raw means; whiskers, 95% CIs.

© 2025 Clavero-Jimeno A et al. *JAMA Network Open*.

eFigure 4. Changes in Mood Outcomes Across All Groups After the 12-Week Intervention, Divided by Sex

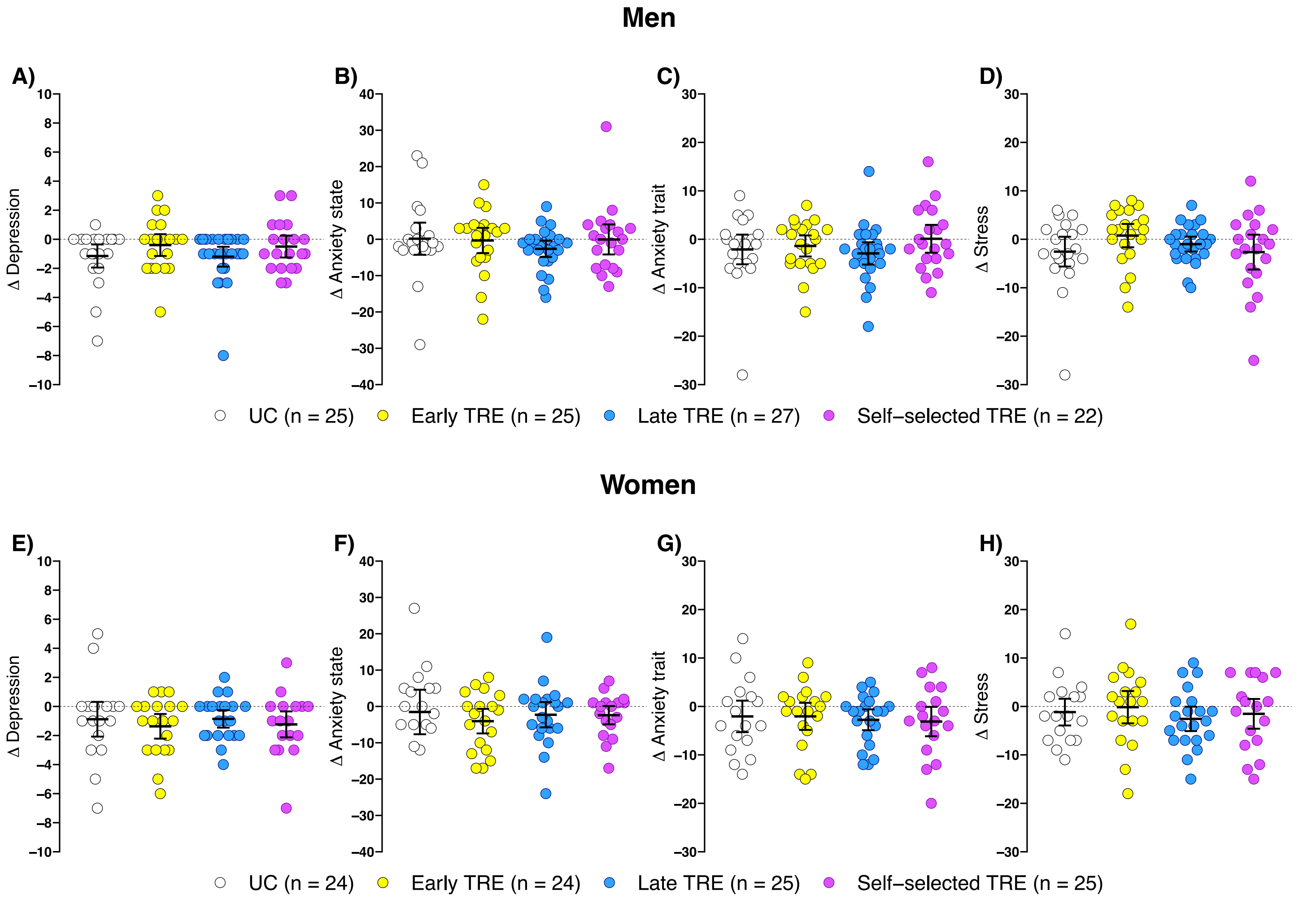

Changes in depression (A and E), state anxiety (B and F), trait anxiety (C and G) and stress (D and H) in men and women among the usual care (UC), early time–restricted eating (TRE), late TRE, and self–selected TRE groups. Changes were calculated as postintervention minus preintervention values. No statistically significant differences in changes in mood outcomes were detected in men and women across all groups after the intervention. Depression was assessed using the Beck Depression Inventory Fast Screen (score range, 0-21 points, with higher scores reflecting more symptoms). State anxiety and trait anxiety were assessed using the State-Trait Anxiety Inventory (score range, 0-60 points, with higher scores reflecting greater anxiety). Stress was assessed using the Perceived Stress Scale (score range, 0-56 points, with higher scores indicating more stress symptoms). Circles represent individual participants' scores; horizontal bars, raw means; whiskers, 95% CIs.

© 2025 Clavero-Jimeno A et al. *JAMA Network Open*.

eFigure 5. Changes in General Health Across All Groups After the 12-Week Intervention, Divided by Sex

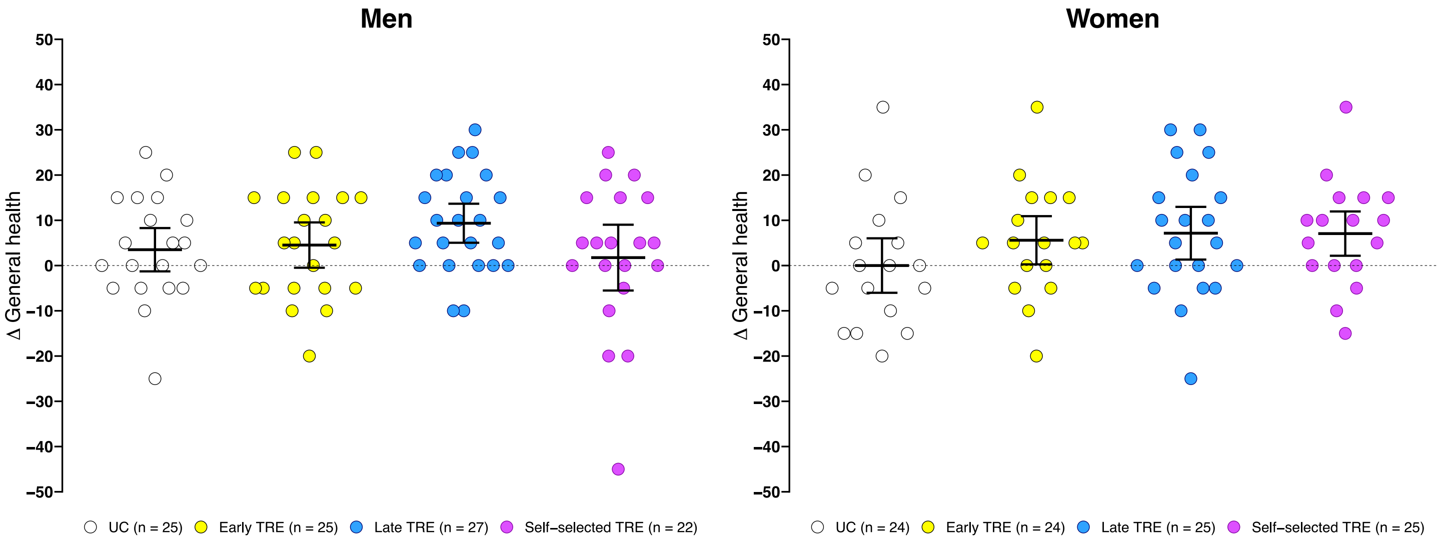

Changes were calculated as the difference between postintervention minus preintervention values. No statistically significant differences in changes in general health were detected across all groups after the intervention. General health was assessed using the Rand 36-Item Short Form Health Survey (score range, 0-100 points, with higher scores reflecting better quality of life). TRE indicates time-restricted eating; UC, usual care. Circles represent individual participants' scores; horizontal bars, raw means; whiskers, 95% CIs.

© 2025 Clavero-Jimeno A et al. *JAMA Network Open*.

eTable 11. Sleep, Mood, and Quality-of-Life End Points at Baseline and After the 12-Week Intervention in Each Intervention Group in Men

End point	UC (n = 25)	Early TRE (n = 25)	Late TRE (n = 27)	Self-selected TRE (n = 22)
	**Mean (95% CI)[a]**	**Mean (95% CI)[a]**	**Mean (95% CI)[a]**	**Mean (95% CI)[a]**
**Sleep**				
Onset				
Preintervention, h:min	00:36 (00:18, 00:54)	00:06 (23:48, 00:24)	00:06 (23:48, 00:24)	00:00 (23:42, 00:18)
Postintervention, h:min	00:30 (00:12, 00:48)	00:00 (23:42, 00:18)	00:06 (23:48, 00:24)	00:00 (23:42, 00:24)
Change, h	-0.1 (-0.4, 0.2)	-0.1 (-0.4, 0.2)	0.0 (-0.2, 0.4)	0.0 (-0.3, 0.3)
Offset				
Preintervention, h:min	07:36 (07:18, 07:54)	07:24 (07:00, 07:42)	07:24 (07:06, 07:42)	07:12 (06:48, 07:30)
Postintervention, h:min	07:30 (07:12, 07:48)	07:18 (07:54, 07:36)	07:00 (07:42, 07:24)	06:54 (06:30, 07:12)
Change, h	-0.1 (-0.5, 0.3)	-0.1 (-0.5, 0.3)	-0.4 (-0.7, 0.0)	-0.3 (-0.7, 0.1)
Period, h[b]				
Preintervention	7.0 (6.7, 7.3)	7.3 (7.0, 7.6)	7.3 (7.0, 7.6)	7.2 (6.8, 7.5)
Postintervention	7.0 (6.7, 7.3)	7.3 (6.9, 7.6)	6.9 (6.6, 7.2)	6.8 (6.5, 7.2)
Change	0.0 (-0.3, 0.3)	0.0 (-0.4, 0.3)	-0.4 (-0.7, -0.1) *	-0.3 (-0.7, 0.0)
Total time, h[c]				
Preintervention	6.0 (5.6, 6.3)	6.2 (5.8, 6.5)	6.3 (5.9, 6.6)	6.2 (5.8, 6.6)
Postintervention	5.8 (5.5, 6.2)	6.1 (5.8, 6.5)	5.9 (5.6, 6.2)	5.9 (5.5, 6.2)
Change	-0.1 (-0.4, 0.2)	-0.1 (-0.4, 0.3)	-0.4 (-0.7, -0.1) *	-0.3 (-0.7, 0.0)
Efficiency, %				
Preintervention	85.2 (82.6, 87.8)	84.0 (81.4, 86.6)	85.7 (83.2, 88.2)	86.2 (83.5, 89.0)
Postintervention	83.4 (80.8, 86.0)	83.9 (81.3, 86.5)	85.8 (83.3, 88.3)	85.7 (83.0, 88.5)
Change	-1.8 (-3.4, -0.2) *	-0.1 (-1.7, 1.5)	0.1 (-1.5, 1.7)	-0.5 (-2.2, 1.1)
Awakenings, No.				
Preintervention	13.3 (12.0, 14.5)	15.8 (14.5, 17.0)	14.6 (13.4, 15.8)	14.1 (12.8, 15.5)
Postintervention	13.3 (12.1, 14.5)	14.9 (13.6, 16.1)	13.4 (12.2, 14.6)	12.7 (11.4, 14.0)
Change	0.0 (-0.9, 0.9)	-0.9 (-1.8, 0.0)	-1.2 (-2.1, -0.3) *	-1.4 (-2.4, -0.5) *
Wake after sleep onset, h				
Preintervention	1.0 (0.8, 1.2)	1.1 (1.0, 1.3)	1.1 (0.9, 1.2)	1.0 (0.8, 1.2)
Postintervention	1.2 (1.0, 1.3)	1.2 (1.0, 1.3)	1.0 (0.8, 1.2)	1.0 (0.8, 1.1)
Change	0.1 (0.0, 0.2)	0.0 (-0.1, 0.1)	-0.1 (-0.2, 0.1)	0.0 (-0.1, 0.1)
Sleep quality score[d]				
Preintervention	6.0 (4.9, 7.0)	5.1 (4.1, 6.2)	6.0 (5.0, 7.0)	6.6 (5.5, 7.7)
Postintervention	5.4 (4.3, 6.5)	5.3 (4.2, 6.4)	5.4 (4.4, 6.4)	5.8 (4.7, 7.0)
Change	-0.6 (-1.5, 0.3)	0.2 (-0.7, 1.1)	-0.7 (-1.5, 0.2)	-0.8 (-1.7, 0.2)
**Mood**				
Depression[e]				
Preintervention	1.8 (0.9, 2.6)	1.4 (0.6, 2.3)	2.3 (1.5, 3.1)	2.1 (1.2, 3)
Postintervention	0.7 (-0.2, 1.6)	1.0 (0.1, 1.9)	1.1 (0.3, 1.9)	1.5 (0.6, 2.4)
Change	-1.1 (-1.9, -0.3) *	-0.4 (-1.2, 0.4)	-1.2 (-1.9, -0.5) *	-0.6 (-1.4, 0.2)
State anxiety[f]				
Preintervention	14.6 (11.0, 18.3)	14.2 (10.5, 17.9)	12.9 (9.3, 16.4)	16.1 (12.2, 20.0)
Postintervention	14.5 (10.6, 18.5)	13.3 (9.4, 17.2)	9.9 (6.3, 13.6)	15.3 (11.2, 19.3)
Change	-0.1 (-3.9, 3.7)	-0.9 (-4.6, 2.8)	-2.9 (-6.3, 0.5)	-0.8 (-4.6, 3.0)
Trait anxiety[f]				
Preintervention	15.8 (12.4, 19.3)	15.8 (12.4, 19.2)	16.3 (13.0, 19.6)	18.8 (15.1, 22.4)
Postintervention	13.9 (10.3, 17.5)	14.3 (10.7, 17.8)	13.4 (10.0, 16.7)	18.5 (14.7, 22.2)
Change	-1.9 (-4.7, 0.8)	-1.5 (-4.2, 1.2)	-2.9 (-5.4, -0.5) *	-0.3 (-3.1, 2.5)

© 2025 Clavero-Jimeno A et al. *JAMA Network Open*.

eTable 11. Sleep, Mood, and Quality-of-Life End Points at Baseline and After the 12-Week Intervention in Each Intervention Group in Men (continued)

End point	UC (n = 25)	Early TRE (n = 25)	Late TRE (n = 27)	Self-selected TRE (n = 22)
	**Mean (95% CI)[a]**	**Mean (95% CI)[a]**	**Mean (95% CI)[a]**	**Mean (95% CI)[a]**
Stress[g]				
Preintervention	23.6 (20.3, 27.0)	21.0 (17.6, 24.3)	22.3 (19.1, 25.5)	25.3 (21.7, 28.8)
Postintervention	20.7 (17.2, 24.2)	21.4 (17.9, 24.9)	21.2 (17.9, 24.4)	22.5 (18.9, 26.1)
Change	-3.0 (-5.8, -0.2) *	0.4 (-2.3, 3.2)	-1.2 (-3.7, 1.4)	-2.8 (-5.6, 0.0)
**Quality-of-life scores[h]**				
Physical functioning				
Preintervention	89.6 (86.3, 92.9)	93.6 (90.3, 96.9)	88.1 (85, 91.3)	92.3 (88.7, 95.8)
Postintervention	92.8 (89.1, 96.4)	96.4 (92.9, 100.0)	95.5 (92.1, 98.9)	97.9 (94.2, 101.5)
Change	3.2 (-0.8, 7.2)	2.8 (-1.1, 6.8)	7.3 (3.6, 11.1) *	5.6 (1.5, 9.7) *
Role limitations				
Due to physical health				
Preintervention	89.0 (80.3, 97.7)	94.0 (85.3, 102.7)	89.8 (81.5, 98.2)	86.4 (77.1, 95.6)
Postintervention	85.8 (76.1, 95.5)	94.3 (84.8, 103.7)	98.8 (89.8, 107.9)	87.8 (78.0, 97.5)
Change	-3.2 (-15.1, 8.8)	0.3 (-11.5, 12.1)	9.0 (-2.3, 20.3)	1.4 (-10.9, 13.7)
Due to emotional problems				
Preintervention	96 (86.9, 105.1)	92 (82.9, 101.1)	96.3 (87.5, 105.1)	69.7 (60, 79.4)
Postintervention	87 (76.9, 97.1)	95.6 (85.7, 105.5)	100.2 (90.8, 109.7)	92.6 (82.4, 102.8)
Change	-9 (-20.1, 2.2)	3.6 (-7.3, 14.6)	3.9 (-6.5, 14.4)	22.9 (11.5, 34.3) *
Vitality				
Preintervention	56.0 (48.4, 63.6)	59.2 (51.6, 66.8)	49.8 (42.4, 57.2)	49.8 (41.6, 57.9)
Postintervention	57.8 (49.8, 65.8)	62.0 (54.1, 69.9)	56.3 (48.7, 63.9)	57.3 (49.0, 65.6)
Change	1.8 (-3.6, 7.2)	2.8 (-2.5, 8)	6.5 (1.4, 11.6) *	7.5 (2.1, 12.9) *
Social functioning				
Preintervention	73.9 (64.2, 83.6)	74.9 (65.2, 84.6)	72.7 (63.4, 82)	67.0 (56.7, 77.4)
Postintervention	75.6 (65.7, 85.5)	73.5 (63.7, 83.4)	75.1 (65.7, 84.6)	67.8 (57.4, 78.2)
Change	1.7 (-3.0, 6.4)	-1.4 (-6.0, 3.2)	2.5 (-2.0, 6.9)	0.7 (-4.0, 5.4)
Bodily pain				
Preintervention	82.7 (75.2, 90.2)	81.7 (74.2, 89.2)	78.6 (71.4, 85.8)	79.8 (71.8, 87.8)
Postintervention	79.2 (70.9, 87.5)	80.7 (72.6, 88.9)	84.4 (76.6, 92.1)	82.7 (74.3, 91.0)
Change	-3.5 (-12.7, 5.7)	-1.0 (-10.0, 8.1)	5.7 (-2.9, 14.4)	2.9 (-6.5, 12.3)
General health				
Preintervention	65.6 (59.7, 71.5)	66.4 (60.5, 72.3)	62.4 (56.7, 68.1)	66.4 (60.0, 72.7)
Postintervention	68.8 (62.4, 75.2)	71.1 (64.8, 77.4)	71.7 (65.7, 77.8)	68.3 (61.8, 74.9)
Change	3.2 (-2.4, 8.8)	4.7 (-0.8, 10.2)	9.3 (4.1, 14.6) *	2.0 (-3.7, 7.6)

Abbreviations: CI, confidence interval; TRE, time-restricted eating; UC, usual care.
[a] Asterisks (*) indicate statistically significant within-group differences as determined by 2-sided t-test ($P < .05$). No adjustments were made for multiple comparisons within groups. Changes were calculated as postintervention minus preintervention values.
[b] Time from sleep onset to sleep offset.
[c] Amount of time classified as sleep within the sleep period.
[d] Assessed using the Pittsburgh Sleep Quality Index (score range, 0-21 points, with higher scores indicating worse sleep quality).
[e] Assessed using the Beck Depression Inventory Fast Screen (score range, 0-21 points, with higher scores reflecting more depressive symptoms).
[f] Assessed using the State-Trait Anxiety Inventory (total score range, 0-60 points for state anxiety and trait anxiety, with higher scores reflecting greater anxiety).
[g] Assessed using the Perceived Stress Scale (score range, 0-56 points, with higher scores indicating greater perceived stress).
[h] Assessed using the Rand 36-Item Short Form Health Survey (score range, 0-100 points, with higher scores reflecting better quality of life).

© 2025 Clavero-Jimeno A et al. *JAMA Network Open*.

eTable 12. Sleep, Mood, and Quality-of-Life End Points at Baseline and After the 12-Week Intervention in Each Intervention Group in Women

**End point**	**UC (n = 24)**	**Early TRE (n = 24)**	**Late TRE (n = 25)**	**Self-selected TRE (n = 25)**
	**Mean (95% CI)[a]**	**Mean (95% CI)[a]**	**Mean (95% CI)[a]**	**Mean (95% CI)[a]**
**Sleep**				
Onset				
Preintervention, h:min	00:06 (23:48, 00:30)	00:24 (00:00, 00:42)	00:18 (00:00, 00:36)	23:54 (23:36, 00:18)
Postintervention, h:min	00:18 (00:00, 00:42)	00:12 (23:54, 00:36)	00:18 (23:54, 00:36)	00:00 (23:36, 00:18)
Change, h	0.2 (-0.1, 0.5)	-0.2 (-0.4, 0.1)	0.0 (-0.3, 0.2)	0.1 (-0.2, 0.3)
Offset				
Preintervention, h:min	07:24 (07:00, 07:42)	07:36 (07:12, 07:54)	07:30 (07:12, 07:54)	07:36 (07:18, 07:54)
Postintervention, h:min	07:12 (06:54, 07:36)	07:30 (07:06, 07:54)	07:24 (07:00, 07:42)	07:36 (07:12, 07:54)
Change, h	-0.2 (-0.4, 0.2)	-0.1 (-0.3, 0.2)	-0.1 (-0.5, 0.1)	0.0 (-0.3, 0.3)
Period, h[b]				
Preintervention	7.2 (6.9, 7.6)	7.2 (6.8, 7.5)	7.2 (6.9, 7.6)	7.6 (7.3, 8.0)
Postintervention	7.0 (6.6, 7.3)	7.3 (6.9, 7.7)	7.1 (6.7, 7.5)	7.6 (7.2, 8.0)
Change	-0.3 (-0.6, 0.1)	0.1 (-0.2, 0.4)	-0.1 (-0.5, 0.2)	-0.1 (-0.4, 0.3)
Total time, h[c]				
Preintervention	6.4 (6.0, 6.8)	6.4 (6.0, 6.8)	6.4 (6.1, 6.8)	6.6 (6.3, 7.0)
Postintervention	6.0 (5.7, 6.4)	6.4 (6.1, 6.8)	6.2 (5.9, 6.6)	6.4 (6.0, 6.8)
Change	-0.4 (-0.7, -0.1) *	0.0 (-0.2, 0.3)	-0.2 (-0.5, 0.1)	-0.2 (-0.5, 0.1)
Efficiency, %				
Preintervention	88.4 (86.5, 90.4)	88.8 (86.8, 90.7)	89.2 (87.3, 91.1)	86.6 (84.7, 88.5)
Postintervention	87.0 (85.0, 89.1)	88.2 (86.2, 90.2)	87.8 (85.8, 89.7)	84.5 (82.5, 86.6)
Change	-1.4 (-3.0, 0.3)	-0.6 (-2.2, 1.0)	-1.4 (-3.0, 0.2)	-2.1 (-3.7, -0.4) *
Awakenings, No.				
Preintervention	13.5 (12.2, 14.8)	13.1 (11.8, 14.4)	13.2 (11.9, 14.6)	15.1 (13.8, 16.4)
Postintervention	12.2 (10.8, 13.6)	13.0 (11.6, 14.4)	12.5 (11.1, 13.8)	14.8 (13.4, 16.1)
Change	-1.3 (-2.5, -0.2) *	-0.1 (-1.2, 1.0)	-0.8 (-1.9, 0.3)	-0.3 (-1.4, 0.8)
Wake after sleep onset, h				
Preintervention	0.8 (0.7, 1.0)	0.8 (0.7, 0.9)	0.8 (0.6, 0.9)	1.0 (0.9, 1.2)
Postintervention	0.9 (0.8, 1.1)	0.9 (0.7, 1.0)	0.9 (0.7, 1.0)	1.2 (1.0, 1.3)
Change	0.1 (-0.1, 0.2)	0.1 (-0.1, 0.2)	0.1 (-0.1, 0.2)	0.1 (0.0, 0.3) *
Sleep quality score[d]				
Preintervention	6.9 (5.7, 8.2)	6.7 (5.4, 8.0)	5.8 (4.6, 7.0)	7.7 (6.5, 8.9)
Postintervention	6.1 (4.7, 7.4)	6.0 (4.6, 7.3)	6.0 (4.7, 7.3)	6.5 (5.2, 7.8)
Change	-0.8 (-2.2, 0.5)	-0.8 (-2.1, 0.6)	0.2 (-1.0, 1.5)	-1.2 (-2.5, 0.1)
**Mood**				
Depression[e]				
Preintervention	2.5 (1.5, 3.4)	2.7 (1.7, 3.7)	2.1 (1.1, 3)	2.9 (1.9, 3.8)
Postintervention	1.7 (0.6, 2.8)	1.6 (0.5, 2.6)	1.2 (0.2, 2.2)	1.6 (0.5, 2.7)
Change	-0.8 (-1.8, 0.2)	-1.1 (-2.1, -0.2) *	-0.9 (-1.8, 0.0)	-1.3 (-2.3, -0.3) *
State anxiety[f]				
Preintervention	16.7 (12.5, 20.9)	20.0 (15.9, 24.2)	15.6 (11.5, 19.7)	20.2 (16.1, 24.3)
Postintervention	14.8 (10.0, 19.5)	16.6 (12.1, 21.2)	13.1 (8.8, 17.5)	17.9 (13.1, 22.6)
Change	-1.9 (-6.4, 2.6)	-3.4 (-7.7, 0.9)	-2.5 (-6.6, 1.6)	-2.4 (-6.9, 2.1)
Trait anxiety[f]				
Preintervention	19.3 (15.1, 23.4)	22.6 (18.5, 26.8)	18.7 (14.7, 22.8)	23.1 (19, 27.1)
Postintervention	17.2 (12.8, 21.6)	20.9 (16.6, 25.2)	15.9 (11.7, 20.1)	20.0 (15.6, 24.4)
Change	-2.0 (-5.2, 1.1)	-1.7 (-4.7, 1.3)	-2.8 (-5.7, 0.1)	-3.0 (-6.2, 0.1)

© 2025 Clavero-Jimeno A et al. *JAMA Network Open*.

eTable 12. Sleep, Mood, and Quality-of-Life End Points at Baseline and After the 12-Week Intervention in Each Intervention Group in Women (continued)

End point	UC (n = 24)	Early TRE (n = 24)	Late TRE (n = 25)	Self-selected TRE (n = 25)
	Mean (95% CI)[a]	Mean (95% CI)[a]	Mean (95% CI)[a]	Mean (95% CI)[a]
Stress[g]				
Preintervention	24.4 (21.2, 27.7)	25.4 (22.2, 28.7)	24.7 (21.5, 27.9)	26.7 (23.5, 29.9)
Postintervention	23.2 (19.5, 26.8)	24.7 (21.2, 28.2)	21.9 (18.5, 25.3)	24.9 (21.2, 28.5)
Change	-1.3 (-4.5, 2.0)	-0.7 (-3.8, 2.4)	-2.8 (-5.8, 0.2)	-1.8 (-5.1, 1.4)
**Quality-of-life scores[h]**				
Physical functioning				
Preintervention	87.3 (82.4, 92.2)	90.1 (85, 95.2)	87 (82.2, 91.8)	84 (79.2, 88.8)
Postintervention	89.1 (83.6, 94.7)	91.7 (86.4, 97.1)	90.9 (85.8, 96)	86.9 (81.4, 92.4)
Change	1.9 (-3.2, 6.9)	1.6 (-3.4, 6.6)	3.9 (-0.7, 8.5)	2.9 (-2.1, 8.0)
Role limitations				
Due to physical health				
Preintervention	80.2 (65.8, 94.6)	87.4 (72.5, 102.3)	84 (69.9, 98.1)	73.0 (58.9, 87.1)
Postintervention	89 (72.3, 105.6)	73.8 (57.9, 89.7)	86.2 (71, 101.3)	81.5 (64.9, 98.1)
Change	8.8 (-8.9, 26.5)	-13.6 (-31.1, 3.9)	2.2 (-14.1, 18.4)	8.5 (-9.1, 26.1)
Due to emotional problems				
Preintervention	84.7 (71.7, 97.8)	73.5 (59.9, 87.1)	92 (79.2, 104.8)	78.7 (65.9, 91.4)
Postintervention	83.8 (68.5, 99.1)	74.3 (59.8, 88.8)	92.6 (78.7, 106.4)	80.5 (65.2, 95.7)
Change	-0.9 (-18, 16.2)	0.8 (-16.2, 17.7)	0.6 (-15.2, 16.4)	1.8 (-15.2, 18.9)
Vitality				
Preintervention	52.7 (46.1, 59.3)	47.6 (40.8, 54.4)	48 (41.5, 54.5)	47.2 (40.7, 53.7)
Postintervention	54.2 (46.8, 61.6)	50.1 (43, 57.2)	56.8 (50, 63.6)	57.1 (49.8, 64.5)
Change	1.5 (-5.1, 8.1)	2.5 (-4.1, 9.1)	8.8 (2.8, 14.9) *	9.9 (3.3, 16.6) *
Social functioning				
Preintervention	65.2 (55.9, 74.5)	66.9 (57.5, 76.4)	72.6 (63.5, 81.7)	69.2 (60.1, 78.3)
Postintervention	66.1 (56.1, 76.2)	61.7 (52, 71.5)	71.1 (61.7, 80.6)	62.7 (52.8, 72.6)
Change	0.9 (-6.6, 8.5)	-5.2 (-12.7, 2.3)	-1.5 (-8.3, 5.3)	-6.5 (-14, 1.0)
Bodily pain				
Preintervention	76.9 (68.0, 85.8)	83.2 (74.0, 92.4)	77.4 (68.7, 86.1)	60.0 (51.3, 68.7)
Postintervention	77.8 (67.7, 87.9)	75.1 (65.4, 84.9)	73.8 (64.5, 83.1)	75.1 (65, 85.2)
Change	0.9 (-9, 10.8)	-8.1 (-17.9, 1.7)	-3.6 (-12.7, 5.4)	15.1 (5.2, 24.9) *
General health				
Preintervention	67.1 (59.8, 74.4)	64.5 (57.1, 72.0)	60.4 (53.2, 67.6)	57.2 (50.0, 64.4)
Postintervention	66.9 (58.9, 74.9)	69.7 (61.9, 77.4)	67.9 (60.4, 75.3)	64.2 (56.3, 72.1)
Change	-0.2 (-6.5, 6.1)	5.2 (-1.1, 11.4)	7.5 (1.8, 13.2) *	7.0 (0.7, 13.3) *

Abbreviations: CI, confidence interval; TRE, time-restricted eating; UC, usual care.

[a] Asterisks (*) indicate statistically significant within-group differences as determined by 2-sided t-test (*P* < .05). No adjustments were made for multiple comparisons within groups. Changes were calculated as postintervention minus preintervention values.

[b] Time from sleep onset to sleep offset.

[c] Amount of time classified as sleep within the sleep period.

[d] Assessed using the Pittsburgh Sleep Quality Index (score range, 0-21 points, with higher scores indicating worse sleep quality).

[e] Assessed using the Beck Depression Inventory Fast Screen (score range, 0-21 points, with higher scores reflecting more depressive symptoms).

[f] Assessed using the State-Trait Anxiety Inventory (total score range, 0-60 points for state anxiety and trait anxiety, with higher scores reflecting greater anxiety).

[g] Assessed using the Perceived Stress Scale (score range, 0-56 points, with higher scores indicating greater perceived stress).

[h] Assessed using the Rand 36-Item Short Form Health Survey (score range, 0-100 points, with higher scores reflecting better quality of life).

© 2025 Clavero-Jimeno A et al. *JAMA Network Open*.

eTable 13. Changes in Sleep End Points in the Time-Restricted Eating Groups Compared With the Usual Care Group After the 12-Week Intervention Considering the Daylight Time in the Analysis, Divided by Sex

**Men**			
**End point**	**Early TRE vs. UC**	**Late TRE vs. UC**	**Self-selected TRE vs. UC**
	**Difference, mean (95% CI)[a]**	**Difference, mean (95% CI)[a]**	**Difference, mean (95% CI)[a]**
**Sleep**			
Onset, h	0.0 (-0.5, 0.6)	0.2 (-0.3, 0.7)	0.1 (-0.4, 0.7)
Offset, h	0.0 (-0.7, 0.7)	-0.3 (-0.9, 0.4)	-0.2 (-0.9, 0.5)
Period, h[b]	-0.1 (-0.7, 0.6)	-0.4 (-1.1, 0.2)	-0.3 (-1.0, 0.3)
Total time, h[c]	0.1 (-0.5, 0.6)	-0.3 (-0.8, 0.3)	-0.2 (-0.8, 0.4)
Efficiency, %	1.7 (-1.3, 4.7)	1.9 (-1.1, 4.8)	1.4 (-1.7, 4.4)
Awakenings, No.	-0.9 (-2.6, 0.8)	-1.2 (-2.9, 0.4)	-1.5 (-3.2, 0.2)
Wake after sleep onset, h	-0.1 (-0.3, 0.1)	-0.2 (-0.4, 0.0)	-0.2 (-0.4, 0.1)
Sleep quality score[d]	0.6 (-1.1, 2.3)	-0.1 (-1.8, 1.5)	-0.4 (-2.1, 1.3)
**Women**			
**End point**	**Early TRE vs. UC**	**Late TRE vs. UC**	**Self-selected TRE vs. UC**
	**Difference, mean (95% CI)[a]**	**Difference, mean (95% CI)[a]**	**Difference, mean (95% CI)[a]**
**Sleep**			
Onset, h	-0.3 (-0.8, 0.1)	-0.2 (-0.7, 0.3)	-0.1 (-0.6, 0.4)
Offset, h	0.1 (-0.5, 0.6)	-0.1 (-0.6, 0.5)	0.1 (-0.4, 0.7)
Period, h[b]	0.4 (-0.2, 1.0)	0.1 (-0.5, 0.8)	0.2 (-0.4, 0.9)
Total time, h[c]	0.4 (-0.1, 0.9)	0.2 (-0.3, 0.7)	0.2 (-0.4, 0.7)
Efficiency, %	0.8 (-2.2, 3.7)	0.0 (-3.0, 3.0)	-0.8 (-3.8, 2.2)
Awakenings, No.	1.2 (-0.8, 3.3)	0.6 (-1.5, 2.6)	1.0 (-1.1, 3.1)
Wake after sleep onset, h	0.0 (-0.2, 0.3)	0.0 (-0.2, 0.3)	0.1 (-0.2, 0.3)
Sleep quality score[d]	0.4 (-2.1, 2.9)	1.4 (-1.1, 3.8)	-0.1 (-2.6, 2.4)

Abbreviations: CI, confidence interval; TRE, time-restricted eating.

[a] Data are presented as estimated mean differences and 95% CI between groups and were calculated by first computing the postintervention minus the preintervention values within each group; then, differences between the groups were computed as early TRE minus late TRE, early TRE minus self-selected TRE, and late TRE minus self-selected TRE. We derived a standardized score which had its minimum value at the Winter Solstice (i.e., -1), indicating the lowest daylight time in the year; and its maximum value at the Summer Solstice (i.e., +1), indicating the longest daylight time in the year. Sample size: UC, n = 25 men and 24 women; early TRE, n = 25 men and 24 women; late TRE, n = 27 men and 25 women; self–selected TRE, n = 22 men and 25 women. No statistically significant differences were detected in changes in sleep outcomes in the TRE groups compared to the UC group considering the daylight time in the analysis in men and women.

[b] Time from sleep onset to sleep offset.

[c] Amount of time classified as sleep within the sleep period.

[d] Assessed using the Pittsburgh Sleep Quality Index (score range, 0-21 points, with higher scores indicating worse sleep quality).

© 2025 Clavero-Jimeno A et al. *JAMA Network Open*.

eTable 14. Changes in Sleep End Points in the Time-Restricted Eating Groups Compared With Each Other After the 12-Week Intervention Considering the Daylight Time in the Analysis, Divided by Sex

Men			
**End point**	**Early TRE vs. late TRE**	**Early TRE vs. self–selected TRE**	**Late TRE vs. self–selected TRE**
	**Difference, mean (95% CI)[a]**	**Difference, mean (95% CI)[a]**	**Difference, mean (95% CI)[a]**
**Sleep**			
Onset, h	-0.2 (-0.7, 0.4)	-0.1 (-0.7, 0.4)	0.1 (-0.5, 0.6)
Offset, h	0.2 (-0.4, 0.9)	0.2 (-0.5, 0.9)	-0.1 (-0.7, 0.6)
Period, h[b]	0.4 (-0.2, 1.0)	0.3 (-0.4, 0.9)	-0.1 (-0.7, 0.5)
Total time, h[c]	0.3 (-0.3, 0.9)	0.2 (-0.3, 0.8)	-0.1 (-0.7, 0.5)
Efficiency, %	-0.2 (-3.2, 2.8)	0.3 (-2.7, 3.4)	0.5 (-2.5, 3.6)
Awakenings, No.	0.3 (-1.4, 2.0)	0.6 (-1.1, 2.3)	0.3 (-1.4, 2.0)
Wake after sleep onset, h	0.1 (-0.1, 0.3)	0.1 (-0.2, 0.3)	0.0 (-0.2, 0.2)
Sleep quality score[d]	0.8 (-0.9, 2.4)	1.0 (-0.7, 2.7)	0.2 (-1.4, 1.9)

Women			
**End point**	**Early TRE vs. late TRE**	**Early TRE vs. self–selected TRE**	**Late TRE vs. self–selected TRE**
	**Difference, mean (95% CI)[a]**	**Difference, mean (95% CI)[a]**	**Difference, mean (95% CI)[a]**
**Sleep**			
Onset, h	-0.1 (-0.6, 0.4)	-0.2 (-0.7, 0.3)	-0.1 (-0.6, 0.4)
Offset, h	0.1 (-0.4, 0.7)	0.0 (-0.6, 0.5)	-0.2 (-0.7, 0.4)
Period, h[b]	0.2 (-0.4, 0.8)	0.1 (-0.5, 0.8)	-0.1 (-0.7, 0.5)
Total time, h[c]	0.2 (-0.2, 0.7)	0.2 (-0.3, 0.8)	0.0 (-0.5, 0.5)
Efficiency, %	0.8 (-2.2, 3.7)	1.6 (-1.4, 4.5)	0.8 (-2.1, 3.8)
Awakenings, No.	0.7 (-1.3, 2.7)	0.2 (-1.8, 2.3)	-0.5 (-2.5, 1.6)
Wake after sleep onset, h	0.0 (-0.2, 0.2)	-0.1 (-0.3, 0.2)	-0.1 (-0.3, 0.2)
Sleep quality score[d]	-1.0 (-3.4, 1.4)	0.5 (-1.9, 2.9)	1.5 (-0.9, 3.8)

Abbreviations: CI, confidence interval; TRE, time-restricted eating.
[a] Data are presented as estimated mean differences and 95% CI between groups and were calculated by first computing the postintervention minus the preintervention values within each group; then, differences between the groups were computed as early TRE minus late TRE, early TRE minus self-selected TRE, and late TRE minus self-selected TRE. We derived a standardized score which had its minimum value at the Winter Solstice (i.e., -1), indicating the lowest daylight time in the year; and its maximum value at the Summer Solstice (i.e., +1), indicating the longest daylight time in the year. Sample size: early TRE, n = 25 men and 24 women; late TRE, n = 27 men and 25 women; self–selected TRE, n = 22 men and 25 women. No statistically significant differences were detected in changes in sleep outcomes between the TRE groups considering the daylight time in the analysis in men and women.
[b] Time from sleep onset to sleep offset.
[c] Amount of time classified as sleep within the sleep period.
[d] Assessed using the Pittsburgh Sleep Quality Index (score range, 0-21 points, with higher scores indicating worse sleep quality).

© 2025 Clavero-Jimeno A et al. *JAMA Network Open*.

eTable 15. Sleep End Points at Baseline and After the 12-Week Intervention Considering the Daylight Time in the Analysis in Men

End point	UC (n = 25)	Early TRE (n = 25)	Late TRE (n = 27)	Self-selected TRE (n = 22)
	**Mean (95% CI)[a]**	**Mean (95% CI)[a]**	**Mean (95% CI)[a]**	**Mean (95% CI)[a]**
**Sleep**				
Onset				
Preintervention, h:min	00:36 (00:18, 00:54)	00:06 (23:48, 00:24)	00:06 (23:48, 00:24)	00:00 (23:42, 00:18)
Postintervention, h:min	00:30 (00:12, 00:48)	00:00 (23:42, 00:18)	00:06 (23:48, 00:24)	00:00 (23:42, 00:24)
Change, h	-0.1 (-0.4, 0.2)	-0.1 (-0.4, 0.2)	0.1 (-0.2, 0.4)	0.0 (-0.3, 0.3)
Offset				
Preintervention, h:min	07:36 (07:12, 07:54)	07:18 (07:00, 07:42)	07:18 (07:00, 07:42)	07:06 (06:48, 07:30)
Postintervention, h:min	07:30 (07:12, 07:54)	07:18 (06:54, 07:36)	07:06 (06:42, 07:24)	06:54 (06:36, 07:18)
Change, h	0.0 (-0.4, 0.3)	-0.1 (-0.4, 0.3)	-0.3 (-0.7, 0.1)	-0.2 (-0.6, 0.2)
Period, h[b]				
Preintervention	6.9 (6.6, 7.3)	7.2 (6.9, 7.6)	7.3 (7.0, 7.6)	7.1 (6.8, 7.5)
Postintervention	7.1 (6.7, 7.4)	7.3 (7.0, 7.6)	6.9 (6.6, 7.2)	6.9 (6.6, 7.2)
Change	0.1 (-0.2, 0.5)	0.1 (-0.3, 0.4)	-0.3 (-0.7, 0.0)	-0.2 (-0.6, 0.1)
Total time, h[c]				
Preintervention	5.9 (5.6, 6.3)	6.1 (5.8, 6.5)	6.2 (5.9, 6.5)	6.2 (5.8, 6.5)
Postintervention	5.9 (5.6, 6.2)	6.2 (5.8, 6.5)	5.9 (5.6, 6.3)	6.0 (5.6, 6.3)
Change	0.0 (-0.3, 0.3)	0.0 (-0.3, 0.4)	-0.3 (-0.6, 0.0)	-0.2 (-0.5, 0.1)
Efficiency, %				
Preintervention	85.1 (82.5, 87.7)	83.8 (81.2, 86.4)	85.5 (83.0, 88.0)	86.1 (83.3, 88.8)
Postintervention	83.6 (81.0, 86.2)	84.0 (81.4, 86.6)	85.9 (83.4, 88.4)	85.9 (83.2, 88.7)
Change	-1.5 (-3.1, 0.2)	0.2 (-1.5, 1.9)	0.4 (-1.3, 2.1)	-0.1 (-1.9, 1.6)
Awakenings, No.				
Preintervention	13.3 (12.1, 14.6)	15.8 (14.6, 17.1)	14.7 (13.4, 15.9)	14.2 (12.9, 15.5)
Postintervention	13.2 (12.0, 14.5)	14.8 (13.6, 16.1)	13.3 (12.1, 14.6)	12.6 (11.3, 14.0)
Change	-0.1 (-1.0, 0.8)	-1.0 (-1.9, -0.1)	-1.3 (-2.3, -0.4) *	-1.6 (-2.6, -0.6) *
Wake after sleep onset, h				
Preintervention	1.0 (0.8, 1.2)	1.1 (1.0, 1.3)	1.1 (0.9, 1.2)	1.0 (0.8, 1.2)
Postintervention	1.1 (1.0, 1.3)	1.2 (1.0, 1.3)	1.0 (0.8, 1.2)	0.9 (0.8, 1.1)
Change	0.1 (0.0, 0.2) *	0.0 (-0.1, 0.1)	-0.1 (-0.2, 0.1)	0.0 (-0.2, 0.1)
Sleep quality score[d]				
Preintervention	5.9 (4.9, 7.0)	5.2 (4.2, 6.3)	6.2 (5.2, 7.3)	6.7 (5.6, 7.8)
Postintervention	5.3 (4.2, 6.4)	5.2 (4.2, 6.3)	5.5 (4.4, 6.5)	5.7 (4.5, 6.8)
Change	-0.6 (-1.5, 0.3)	0.0 (-0.9, 0.9)	-0.8 (-1.7, 0.1)	-1.0 (-2.0, -0.1)

Abbreviations: CI, confidence interval; TRE, time-restricted eating; UC, usual care.
[a] Asterisks (*) indicate statistically significant within-group differences as determined by 2-sided t-test ($P < .05$). No adjustments were made for multiple comparisons within groups. Changes were calculated as postintervention minus preintervention values. We derived a standardized score which had its minimum value at the Winter Solstice (i.e., -1), indicating the lowest daylight time in the year; and its maximum value at the Summer Solstice (i.e., +1), indicating the longest daylight time in the year.
[b] Time from sleep onset to sleep offset.
[c] Amount of time classified as sleep within the sleep period.
[d] Assessed using the Pittsburgh Sleep Quality Index (score range, 0-21 points, with higher scores indicating worse sleep quality).

© 2025 Clavero-Jimeno A et al. *JAMA Network Open*.

eTable 16. Sleep End Points at Baseline and After the 12-Week Intervention Considering the Daylight Time in the Analysis in Women

End point	UC (n = 24)	Early TRE (n = 24)	Late TRE (n = 25)	Self-selected TRE (n = 25)
	**Mean (95% CI)[a]**	**Mean (95% CI)[a]**	**Mean (95% CI)[a]**	**Mean (95% CI)[a]**
**Sleep**				
Onset				
Preintervention, h:min	00:06 (23:48, 00:30)	00:24 (00:00, 00:42)	00:18 (00:00, 00:36)	23:54 (23:36, 00:18)
Postintervention, h:min	00:18 (23:54, 00:42)	00:12 (23:54, 00:36)	00:12 (23:54, 00:36)	00:00 (23:36, 00:18)
Change, h	0.2 (-0.1, 0.5)	-0.2 (-0.4, 0.1)	-0.1 (-0.3, 0.2)	0.0 (-0.2, 0.3)
Offset				
Preintervention, h:min	07:18 (07:00, 07:42)	07:30 (07:12, 07:54)	07:30 (07:12, 07:54)	07:36 (07:12, 07:54)
Postintervention, h:min	07:18 (06:54, 07:36)	07:30 (07:12, 07:54)	07:24 (07:00, 07:48)	07:36 (07:12, 08:00)
Change, h	-0.1 (-0.4, 0.2)	0.0 (-0.3, 0.3)	-0.1 (-0.4, 0.2)	0.1 (-0.3, 0.3)
Period, h[b]				
Preintervention	7.2 (6.8, 7.6)	7.2 (6.8, 7.5)	7.2 (6.8, 7.5)	7.6 (7.2, 8.0)
Postintervention	7.0 (6.6, 7.4)	7.3 (7.0, 7.7)	7.1 (6.8, 7.5)	7.6 (7.3, 8.0)
Change	-0.2 (-0.6, 0.1)	0.2 (-0.2, 0.5)	-0.1 (-0.4, 0.3)	0.0 (-0.3, 0.4)
Total time, h[c]				
Preintervention	6.4 (6.0, 6.8)	6.4 (6.0, 6.7)	6.4 (6.1, 6.8)	6.6 (6.3, 7.0)
Postintervention	6.0 (5.7, 6.4)	6.4 (6.1, 6.8)	6.3 (5.9, 6.6)	6.4 (6.1, 6.8)
Change	-0.3 (-0.6, -0.1)	0.1 (-0.2, 0.3)	-0.2 (-0.5, 0.1)	-0.2 (-0.5, 0.1)
Efficiency, %				
Preintervention	88.7 (86.7, 90.6)	89.0 (87.1, 91.0)	89.4 (87.5, 91.3)	86.9 (85.0, 88.8)
Postintervention	86.8 (84.7, 88.8)	87.8 (85.8, 89.8)	87.5 (85.5, 89.5)	84.2 (82.2, 86.2)
Change	-1.9 (-3.6, -0.2)	-1.2 (-2.8, 0.5)	-1.9 (-3.5, -0.3)	-2.7 (-4.4, -1.1)
Awakenings, No.				
Preintervention	13.5 (12.2, 14.9)	13.1 (11.8, 14.5)	13.3 (11.9, 14.6)	15.1 (13.8, 16.4)
Postintervention	12.1 (10.7, 13.5)	13.0 (11.6, 14.3)	12.4 (11.1, 13.8)	14.7 (13.3, 16.1)
Change	-1.4 (-2.5, -0.2)	-0.1 (-1.3, 1.0)	-0.8 (-1.9, 0.3)	-0.4 (-1.6, 0.8)
Wake after sleep onset, h				
Preintervention	0.8 (0.7, 1.0)	0.8 (0.6, 0.9)	0.8 (0.6, 0.9)	1.0 (0.8, 1.1)
Postintervention	0.9 (0.8, 1.1)	0.9 (0.8, 1.0)	0.9 (0.7, 1.0)	1.2 (1.0, 1.3)
Change	0.1 (0.0, 0.2)	0.1 (0.0, 0.3)	0.1 (0.0, 0.3)	0.2 (0.1, 0.3) *
Sleep quality score[d]				
Preintervention	6.9 (5.7, 8.2)	6.7 (5.5, 8.0)	5.8 (4.6, 7.0)	7.7 (6.5, 8.9)
Postintervention	5.8 (4.3, 7.2)	6.0 (4.6, 7.3)	6.0 (4.7, 7.3)	6.4 (5.1, 7.8)
Change	-1.2 (-2.5, 0.2)	-0.8 (-2.1, 0.6)	0.2 (-1.1, 1.5)	-1.3 (-2.6, 0.1)

Abbreviations: CI, confidence interval; TRE, time-restricted eating; UC, usual care.

[a] Asterisks (*) indicate statistically significant within-group differences as determined by 2-sided t-test (*P* < .05). No adjustments were made for multiple comparisons within groups. Changes were calculated as postintervention minus preintervention values. We derived a standardized score which had its minimum value at the Winter Solstice (i.e., -1), indicating the lowest daylight time in the year; and its maximum value at the Summer Solstice (i.e., +1), indicating the longest daylight time in the year.

[b] Time from sleep onset to sleep offset.

[c] Amount of time classified as sleep within the sleep period.

[d] Assessed using the Pittsburgh Sleep Quality Index (score range, 0-21 points, with higher scores indicating worse sleep quality).

© 2025 Clavero-Jimeno A et al. *JAMA Network Open*.

eReferences

1. da Silva VS, Vieira MFS. International society for the advancement of kinanthropometry (Isak) global: International accreditation scheme of the competent anthropometrist. *Revista Brasileira de Cineantropometria e Desempenho Humano*. 2020;22:1-6. doi:10.1590/1980-0037.2020v22e70517
2. Migueles JH, Rowlands A V., Huber F, Sabia S, van Hees VT. GGIR: A Research Community–Driven Open Source R Package for Generating Physical Activity and Sleep Outcomes From Multi-Day Raw Accelerometer Data. *J Meas Phys Behav*. 2019;2(3):188-196. doi:10.1123/jmpb.2018-0063
3. van Hees VT, Renström F, Wright A, et al. Estimation of daily energy expenditure in pregnant and non-pregnant women using a wrist-worn tri-axial accelerometer. *PLoS One*. 2011;6(7):e22922. doi:10.1371/journal.pone.0022922
4. van Hees VT, Sabia S, Jones SE, et al. Estimating sleep parameters using an accelerometer without sleep diary. *Sci Rep*. 2018;8(1):12975. doi:10.1038/s41598-018-31266-z
5. Buysse DJ, Reynolds CF, Monk TH, Berman SR, Kupfer DJ. The Pittsburgh Sleep Quality Index: a new instrument for psychiatric practice and research. *Psychiatry Res*. 1989;28(2):193-213. doi:10.1016/0165-1781(89)90047-4
6. Beck AT, Steer RA, Carbin MG. Psychometric properties of the Beck Depression Inventory: Twenty-five years of evaluation. *Clin Psychol Rev*. 1988;8(1):77-100. doi:10.1016/0272-7358(88)90050-5
7. Skapinakis P. Spielberger State-Trait Anxiety Inventory. In: *Encyclopedia of Quality of Life and Well-Being Research*. Springer Netherlands; 2014:6261-6264. doi:10.1007/978-94-007-0753-5_2825
8. Cohen S, Kamarck T, Mermelstein R. A global measure of perceived stress. *J Health Soc Behav*. 1983;24(4):385-396. http://www.ncbi.nlm.nih.gov/pubmed/6668417
9. Hays RD, Morales LS. The RAND-36 measure of health-related quality of life. *Ann Med*. 2001;33(5):350-357. doi:10.3109/07853890109002089
10. Mattingly SM, Grover T, Martinez GJ, et al. The effects of seasons and weather on sleep patterns measured through longitudinal multimodal sensing. *npj Digital Medicine 2021 4:1*. 2021;4(1):1-15. doi:10.1038/s41746-021-00435-2

© 2025 Clavero-Jimeno A et al. *JAMA Network Open*.
