# Supplementary material for: Time-Restricted Eating and Sleep, Mood, and Quality of Life in Adults With Overweight or Obesity: A Secondary Analysis of a Randomized Clinical Trial
Source: JAMA Netw Open. 2025 Jun 25;8(6):e2517268. doi: 10.1001/jamanetworkopen.2025.17268 (PMC12199060; doi:10.1001/jamanetworkopen.2025.17268)
Supplement: Supplement 4. — Data Sharing Statement [file jamanetwopen-e2517268-s004.pdf]

Data Sharing Statement

Data
Additional Information: - Name of the trial registry: Efficacy and Feasibility of Time-restricted Eating on Cardiometabolic Health in Adults With Overweight/Obesity (EXTREME) - Registry's URL: https://clinicaltrials.gov/study/NCT05310721?cond=extreme%20and%20time-restricted%20eating&rank=1#more-information - Trial registration number: NCT05310721
Data available: No

Additional Information
Explanation for why data not available: Due to privacy concerns, the datasets used in this study are not publicly available; however, researchers can request access to specific individual-level data for academic use only, within 36 months following the publication date, after de-identification. Proposals should be directed to corresponding authors. Individual requests for anonymized data will be considered afterwards on a case-by-case basis.
